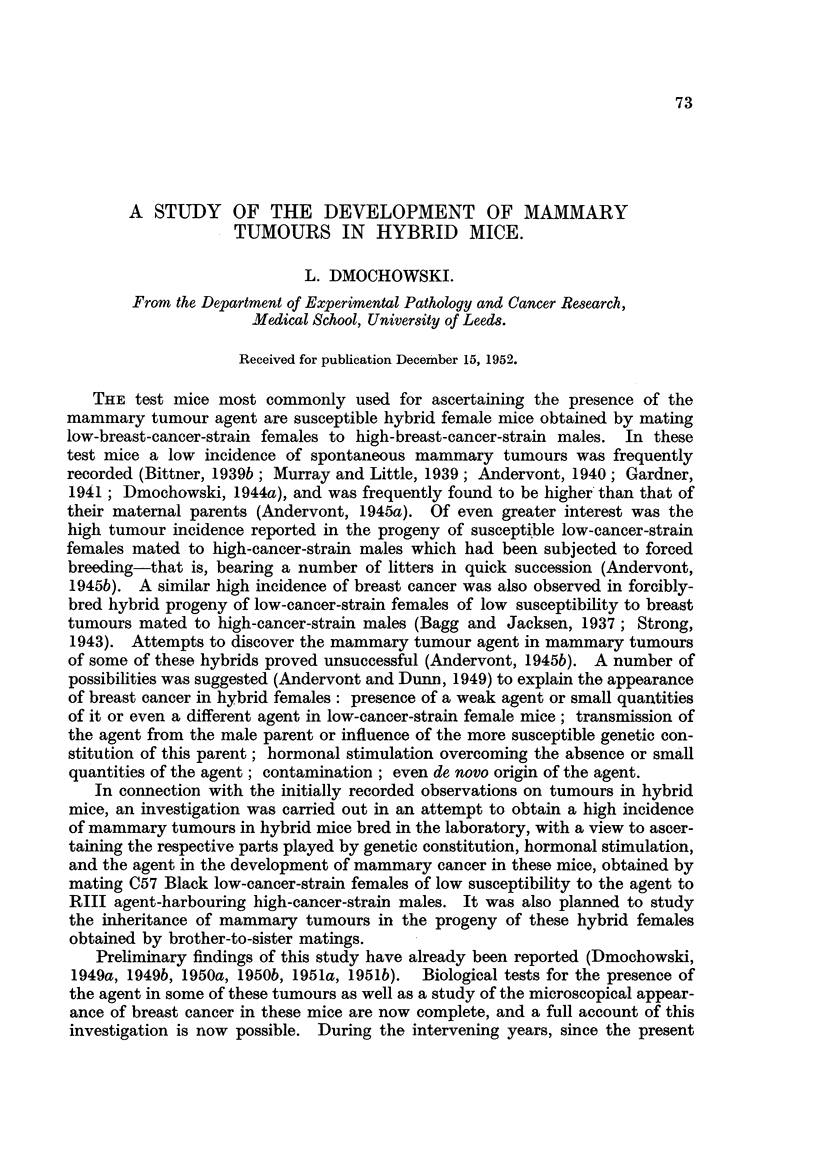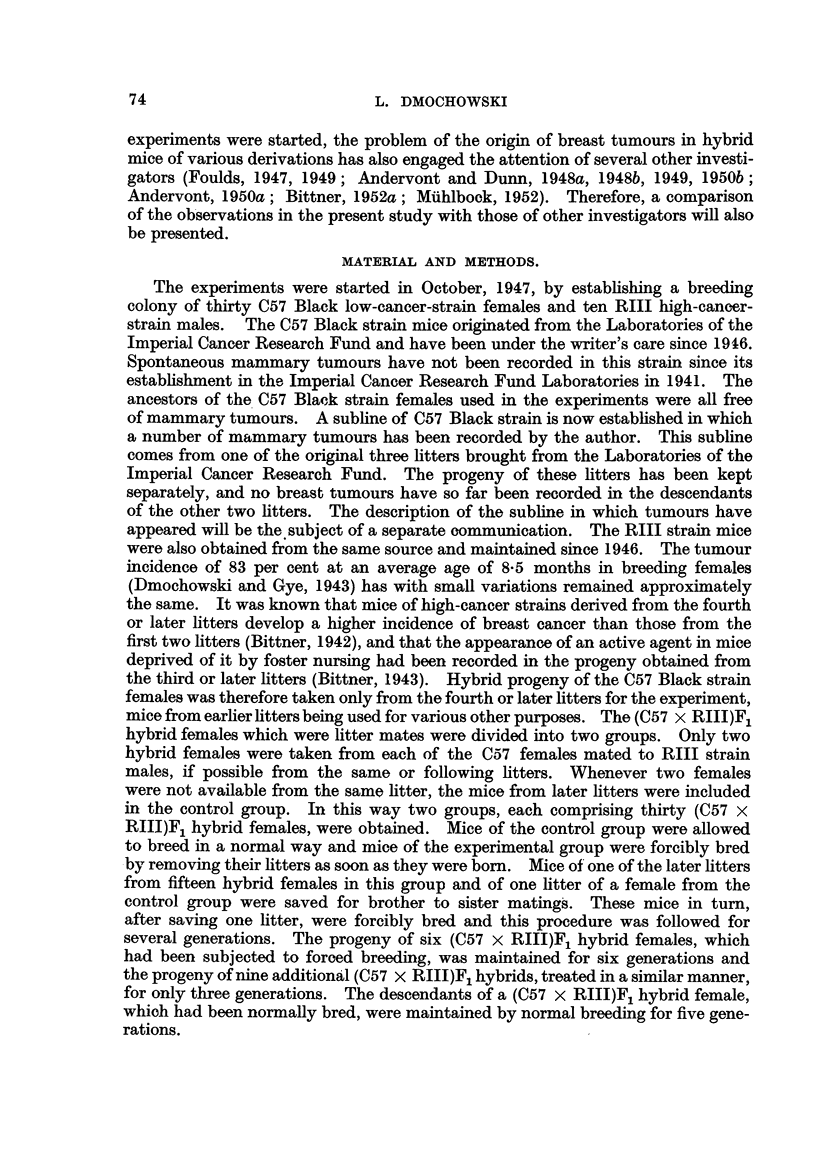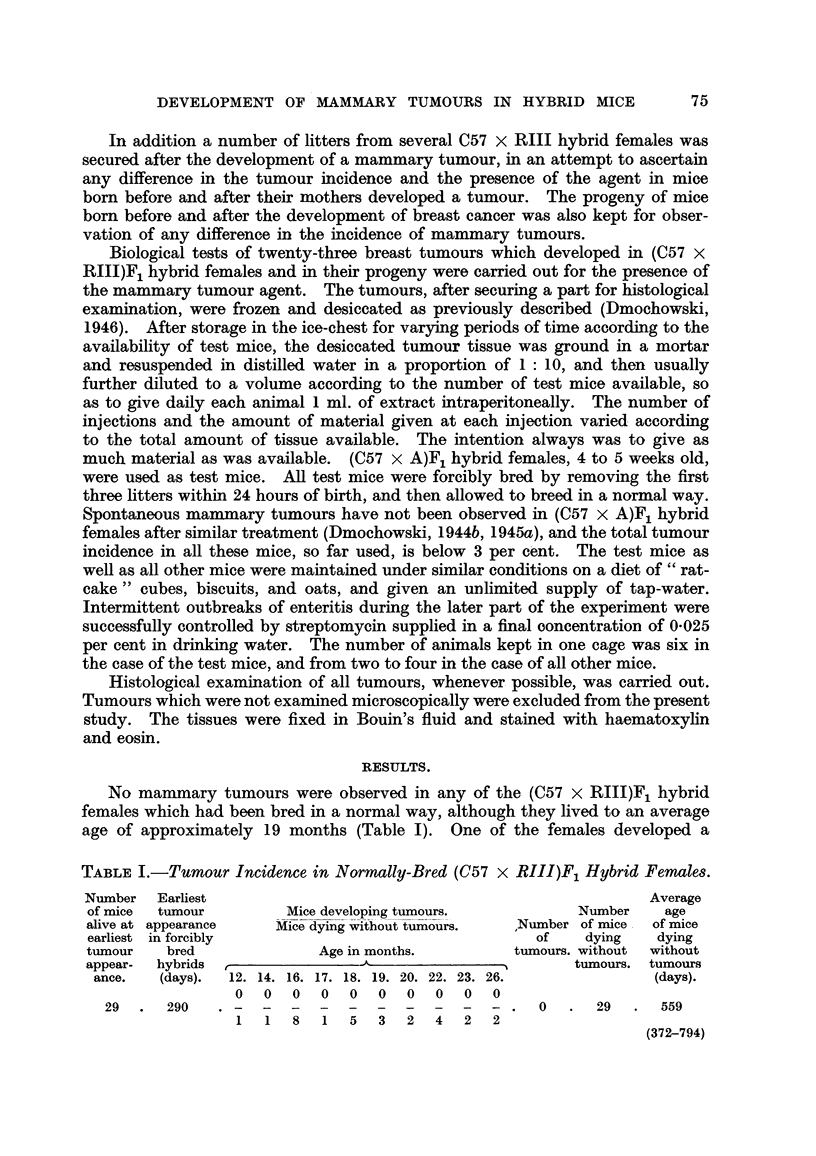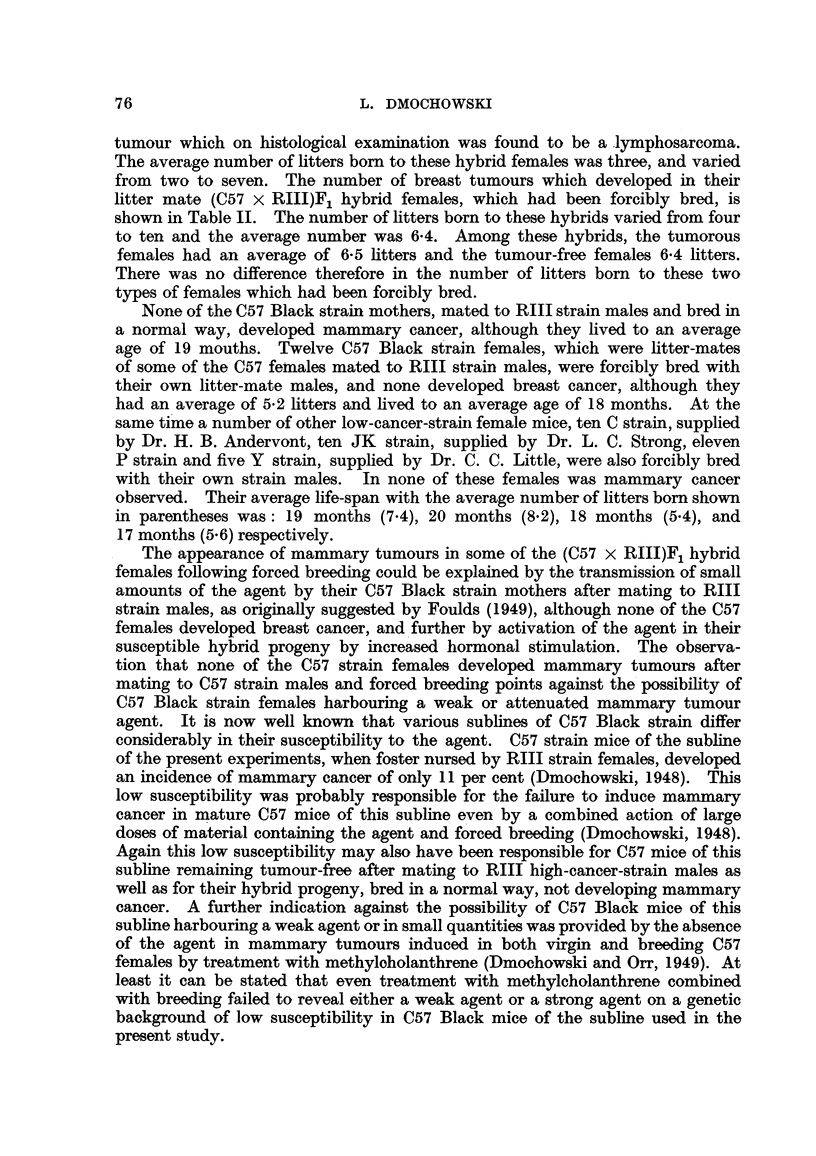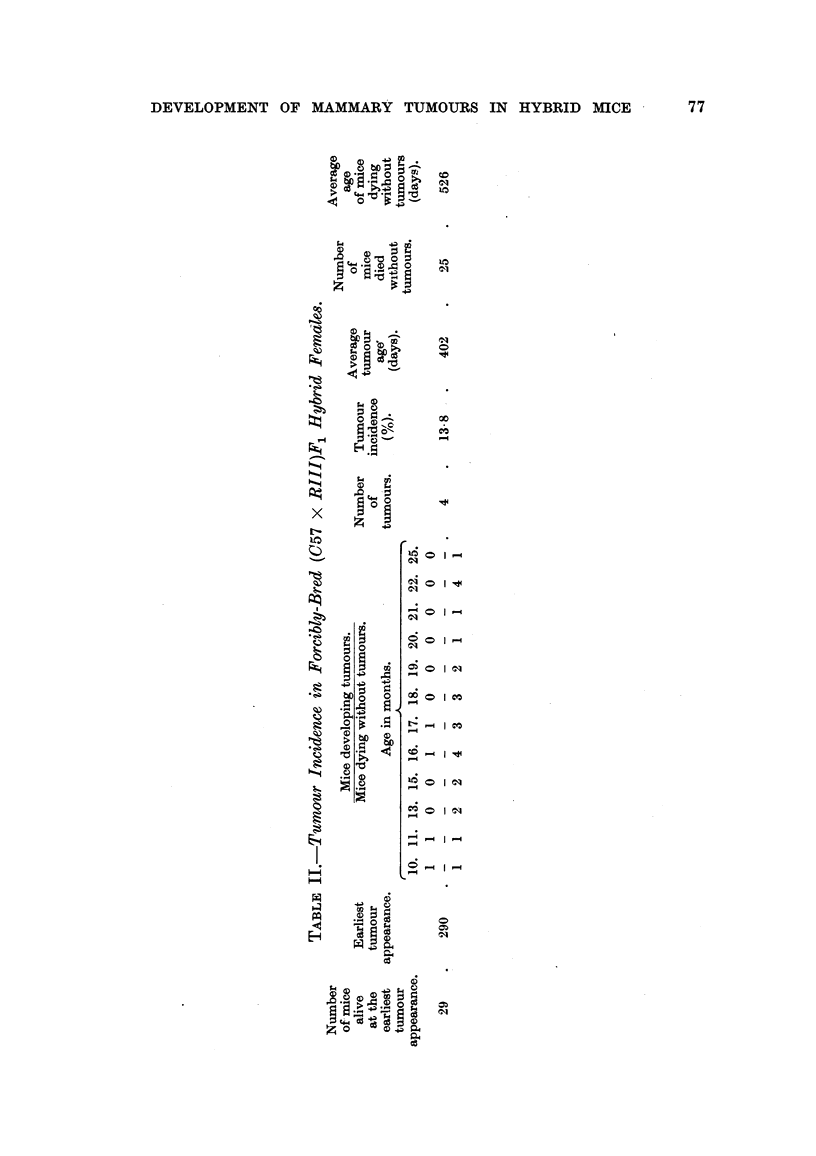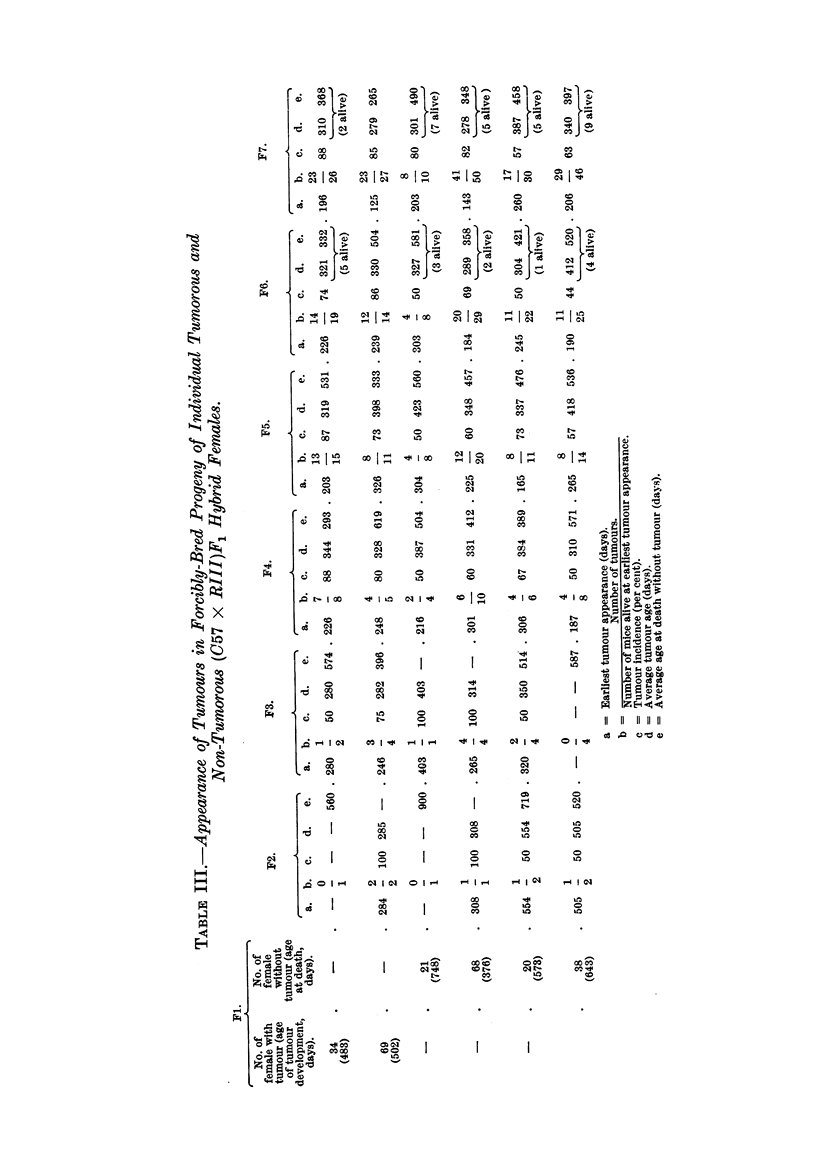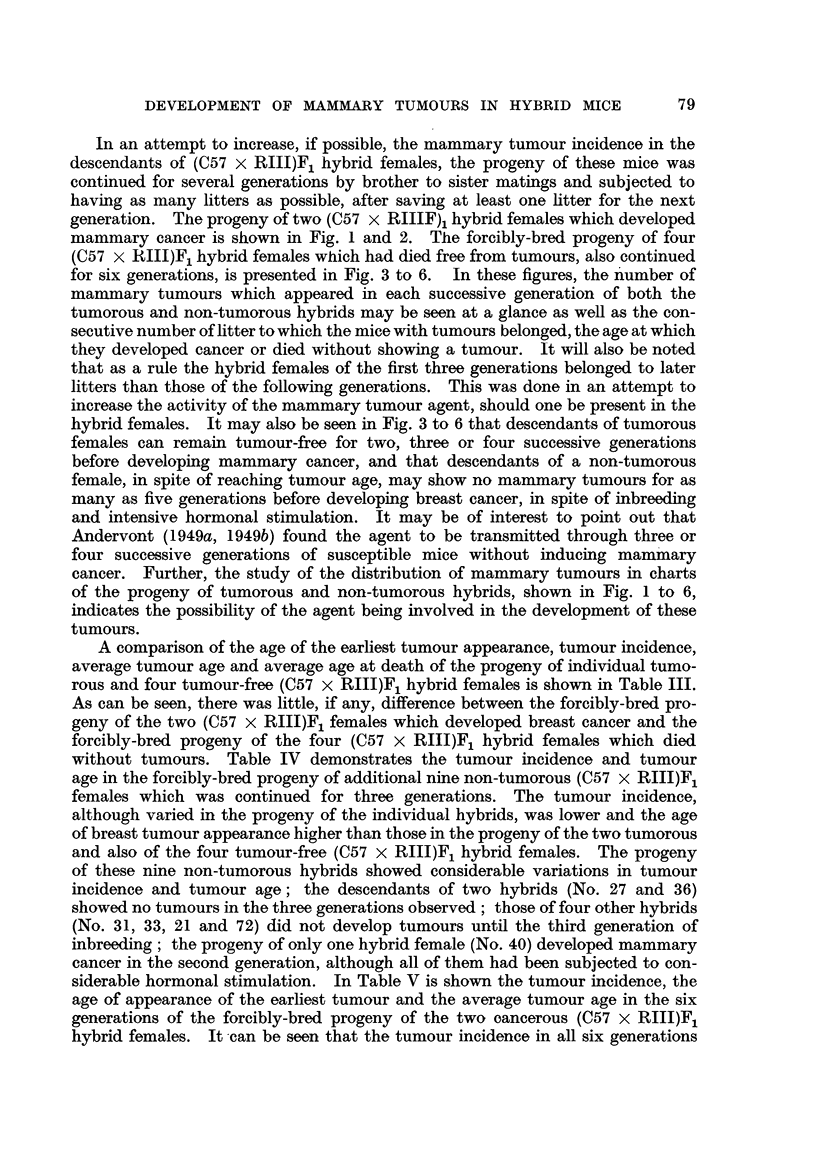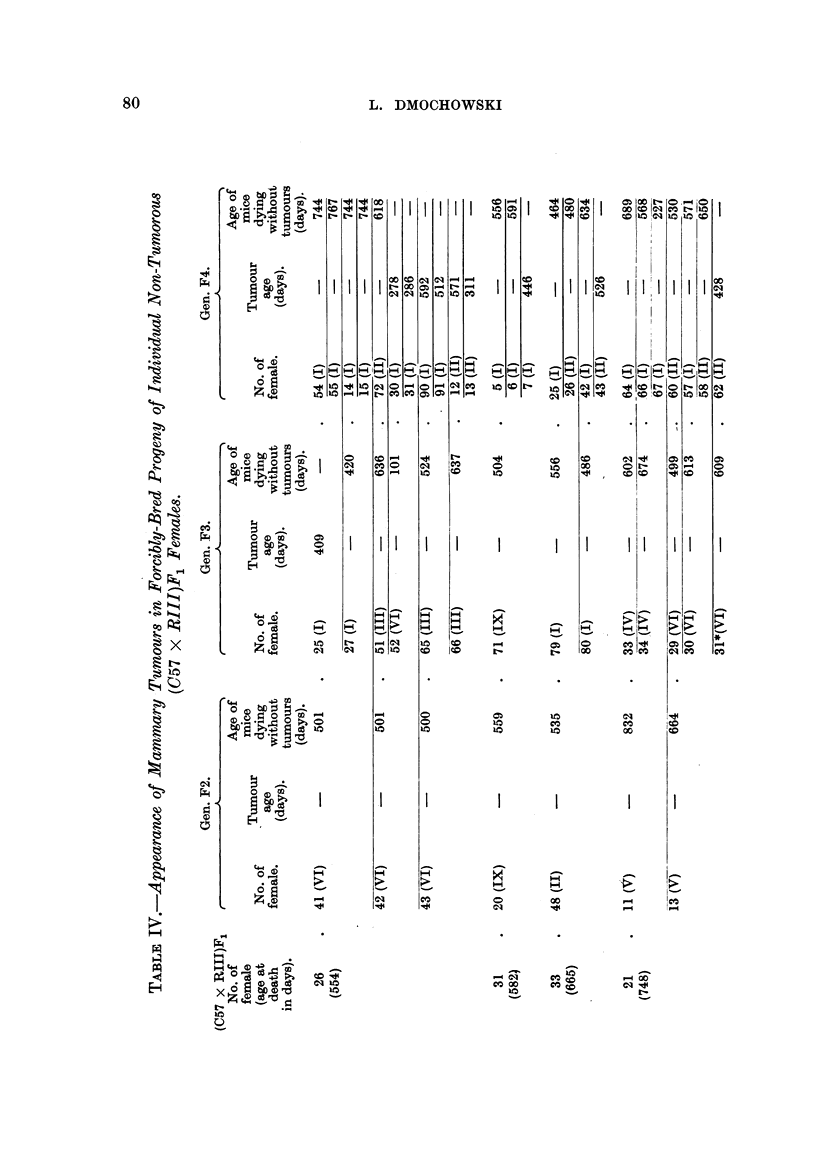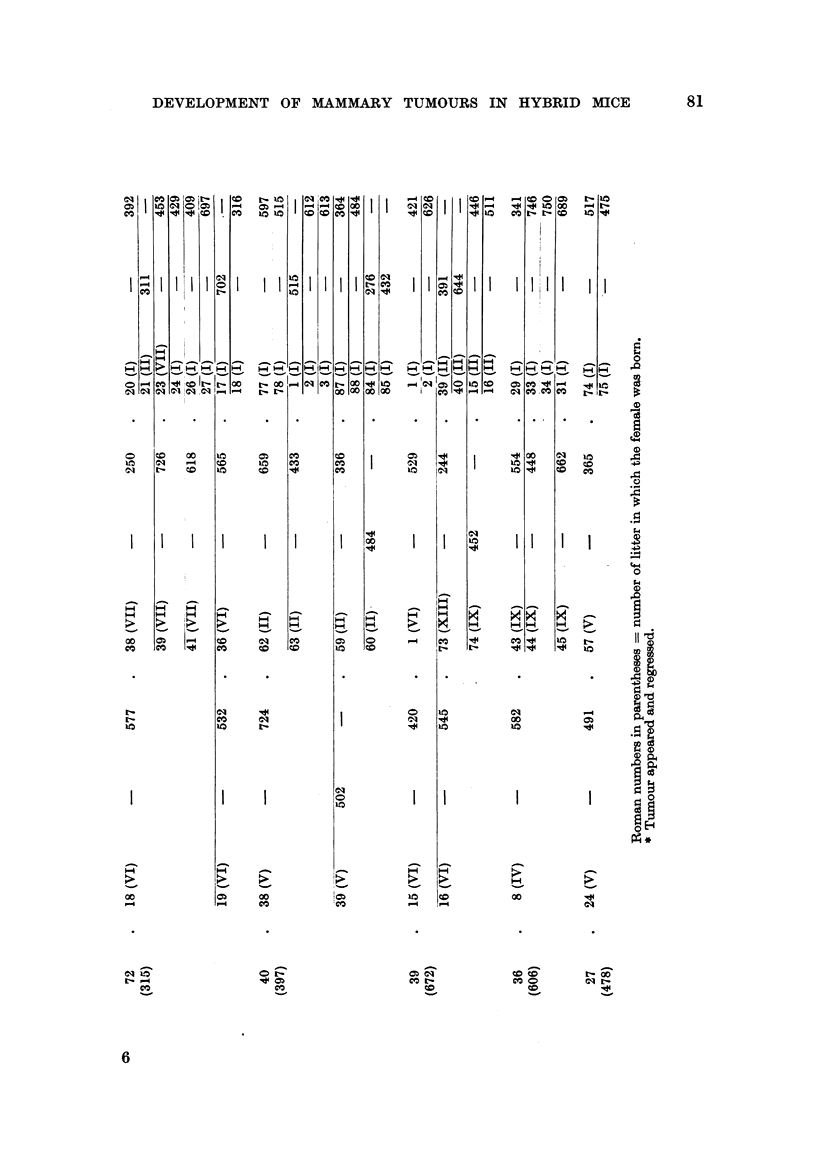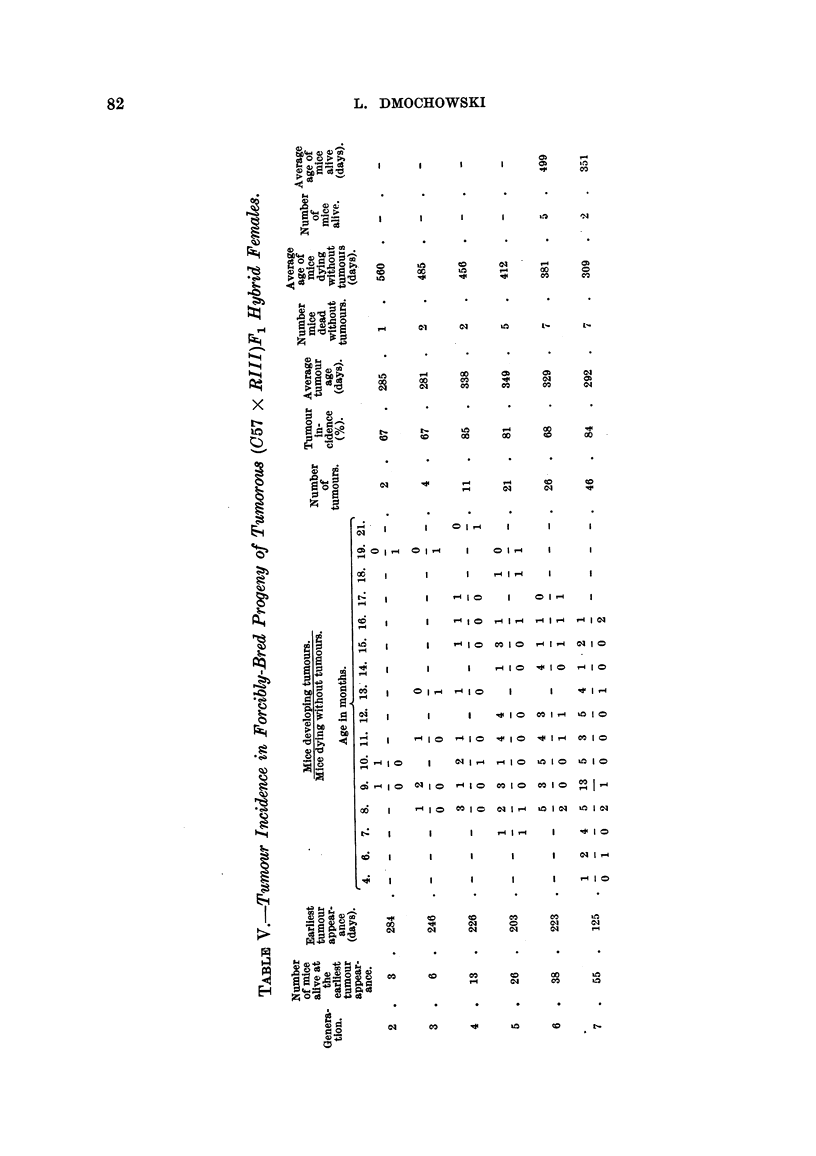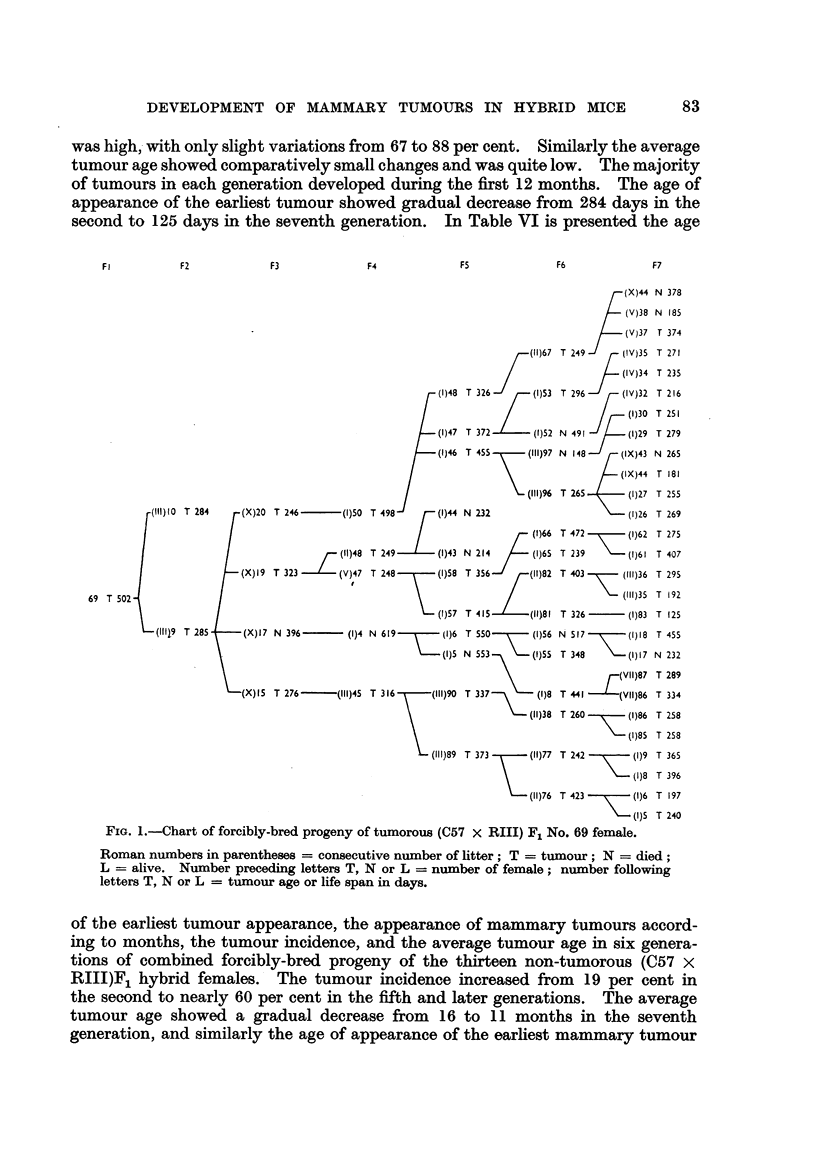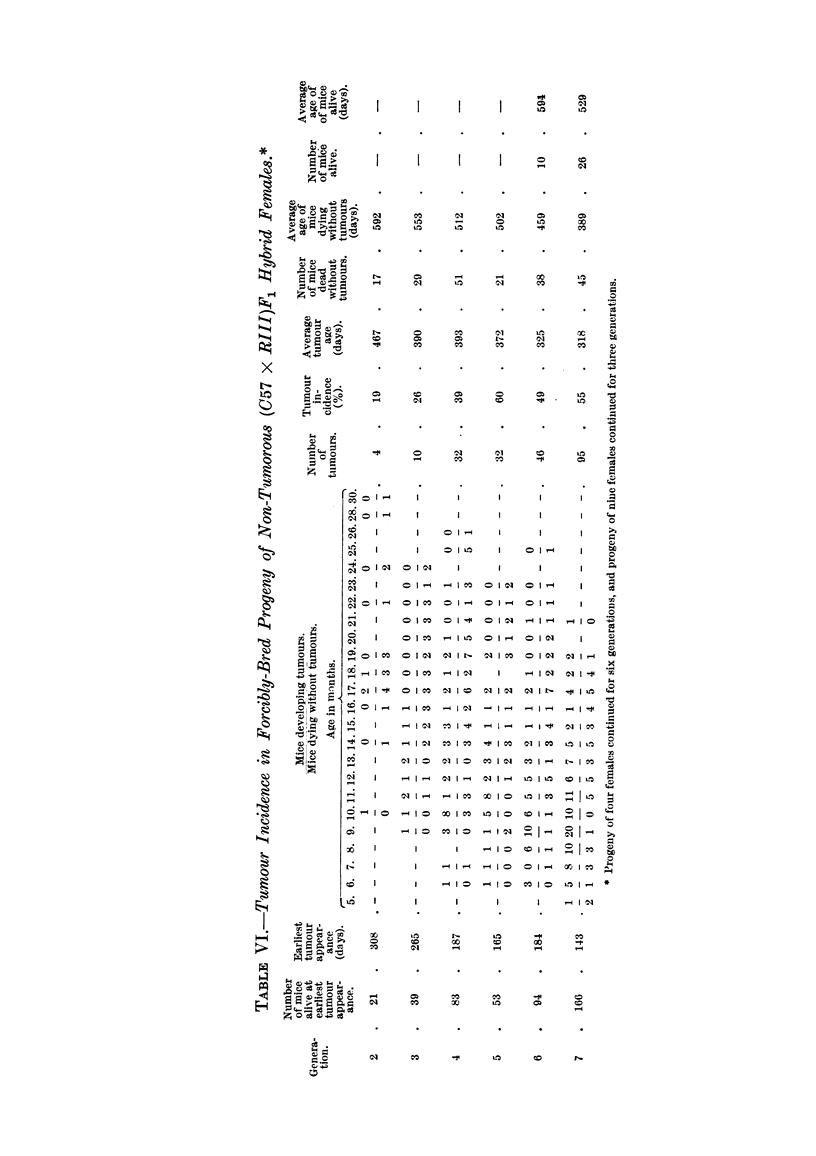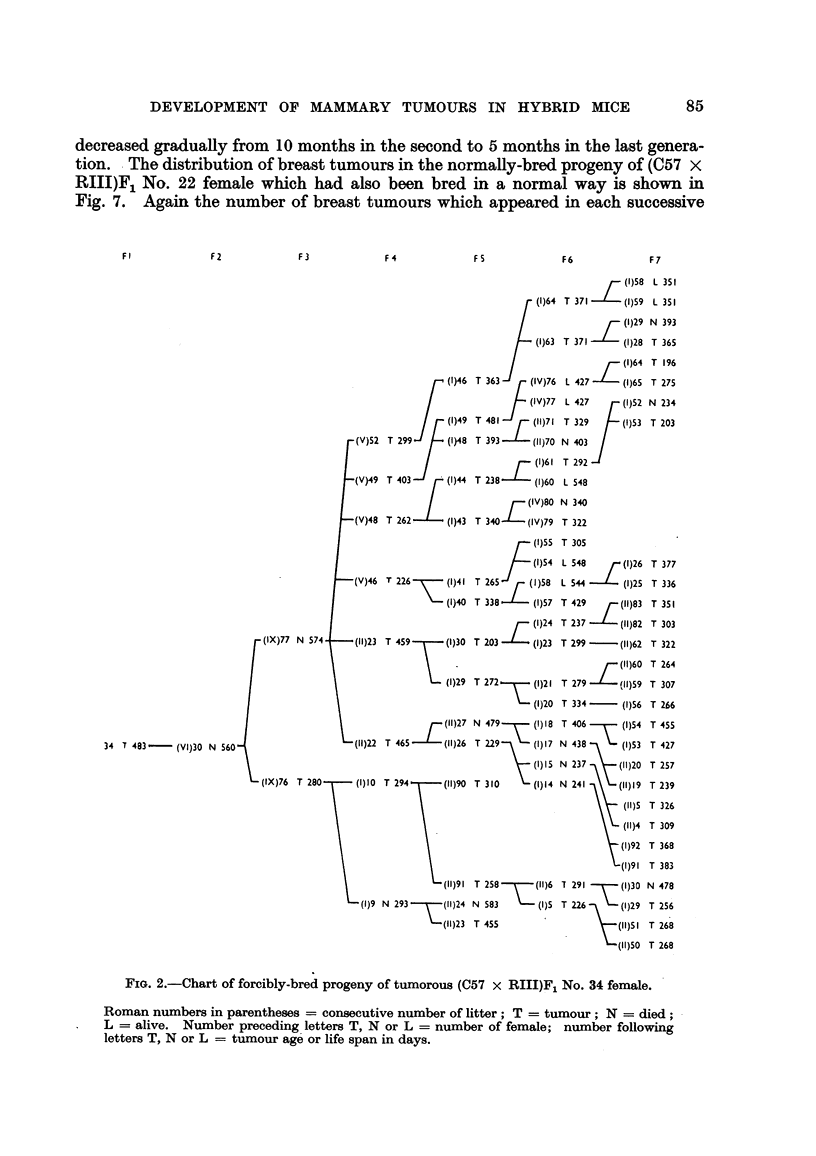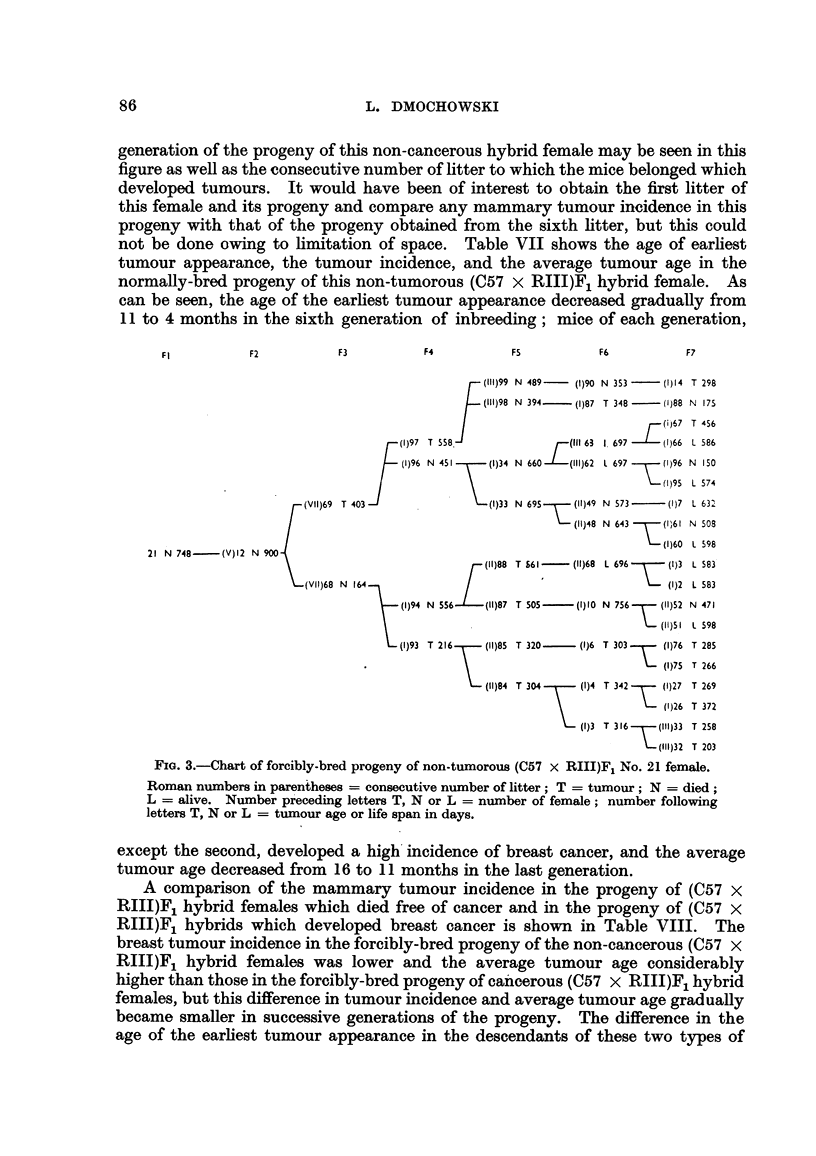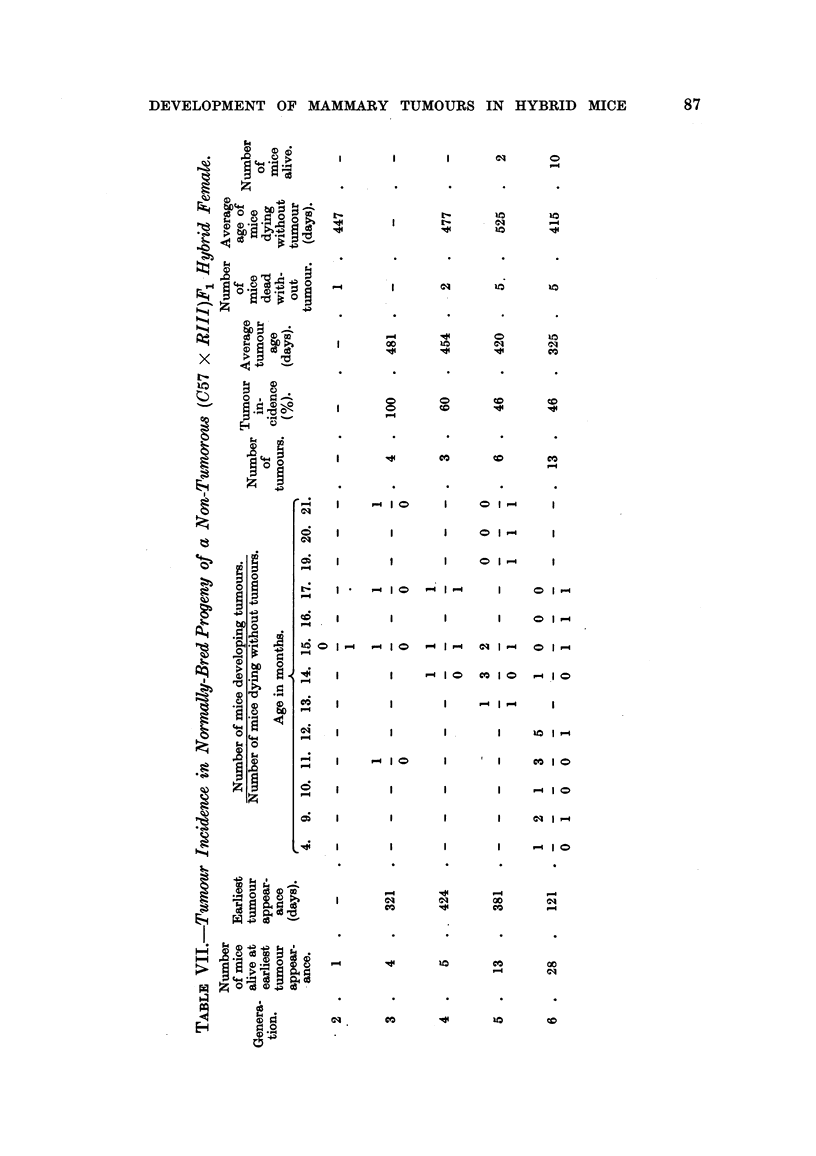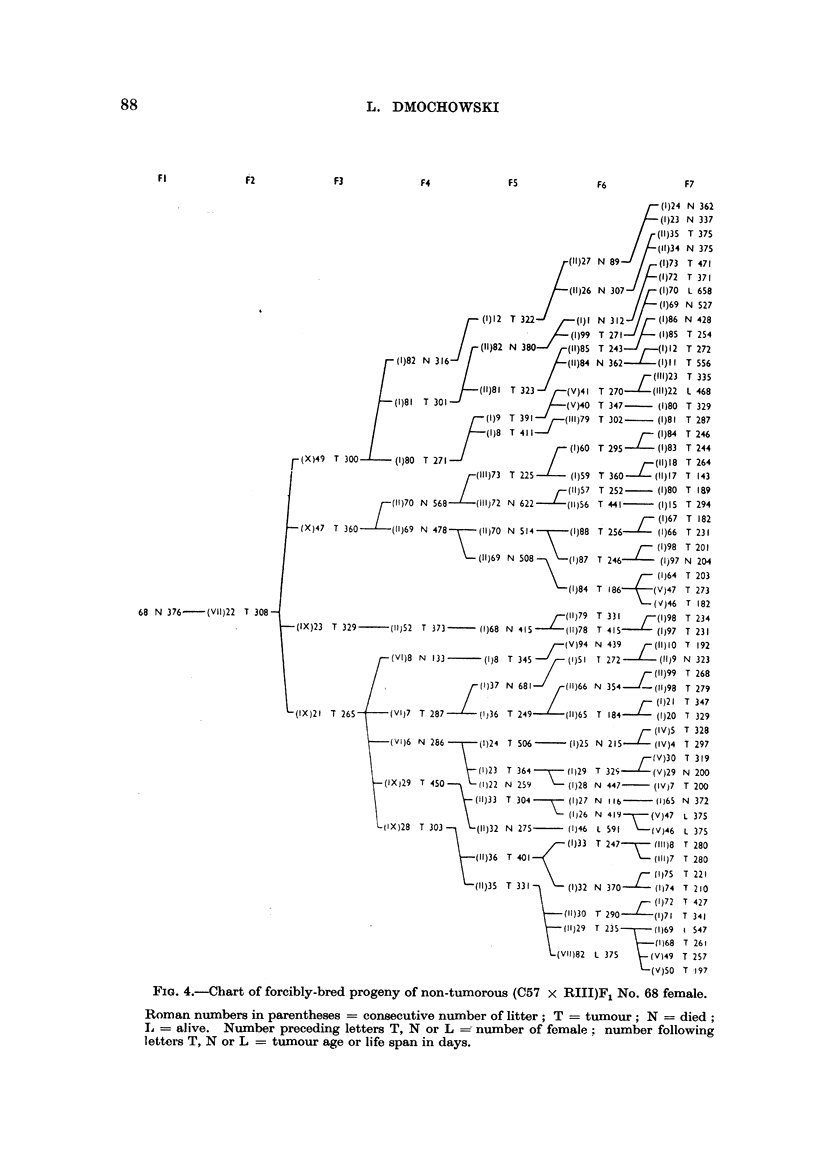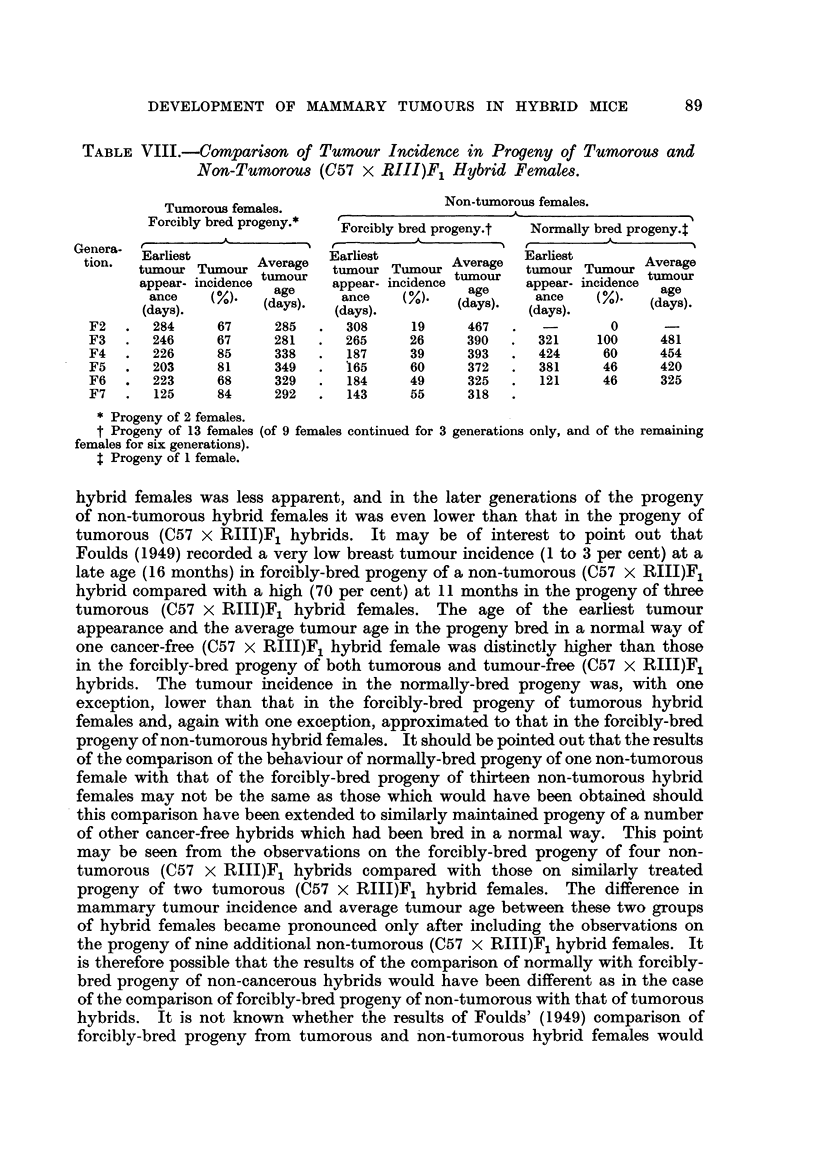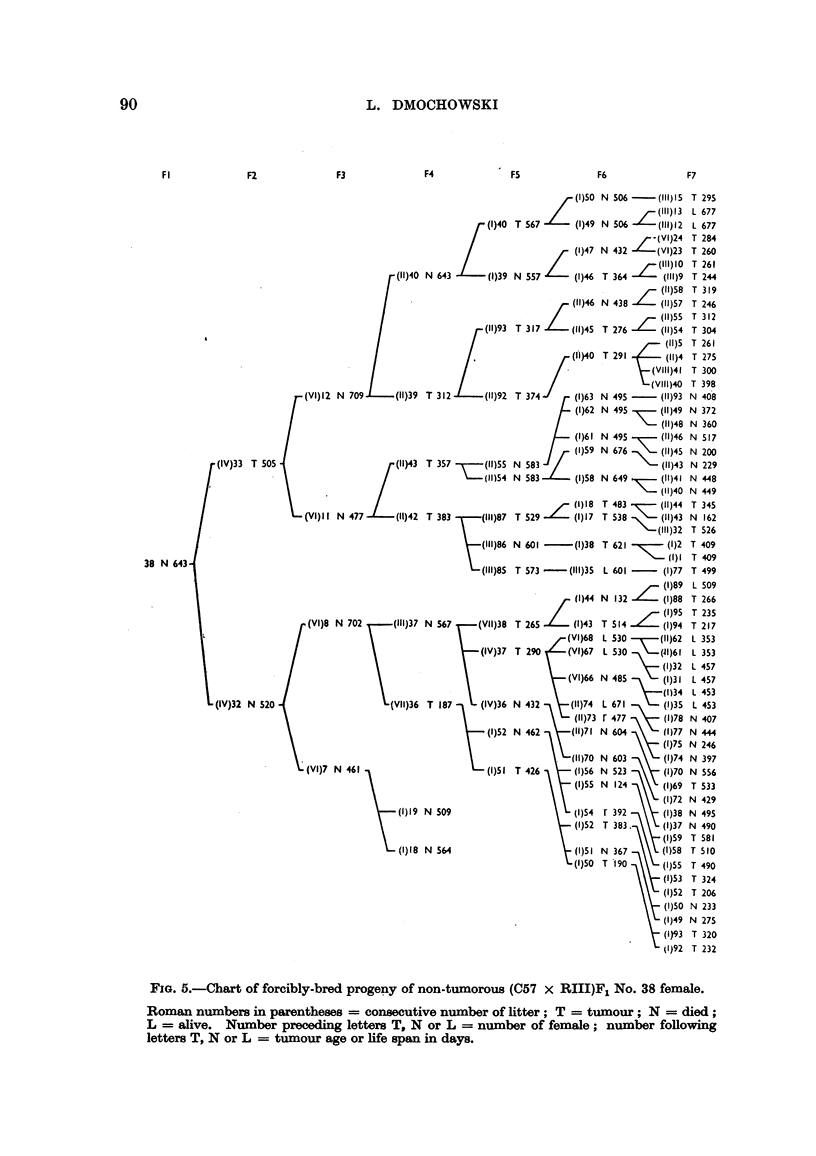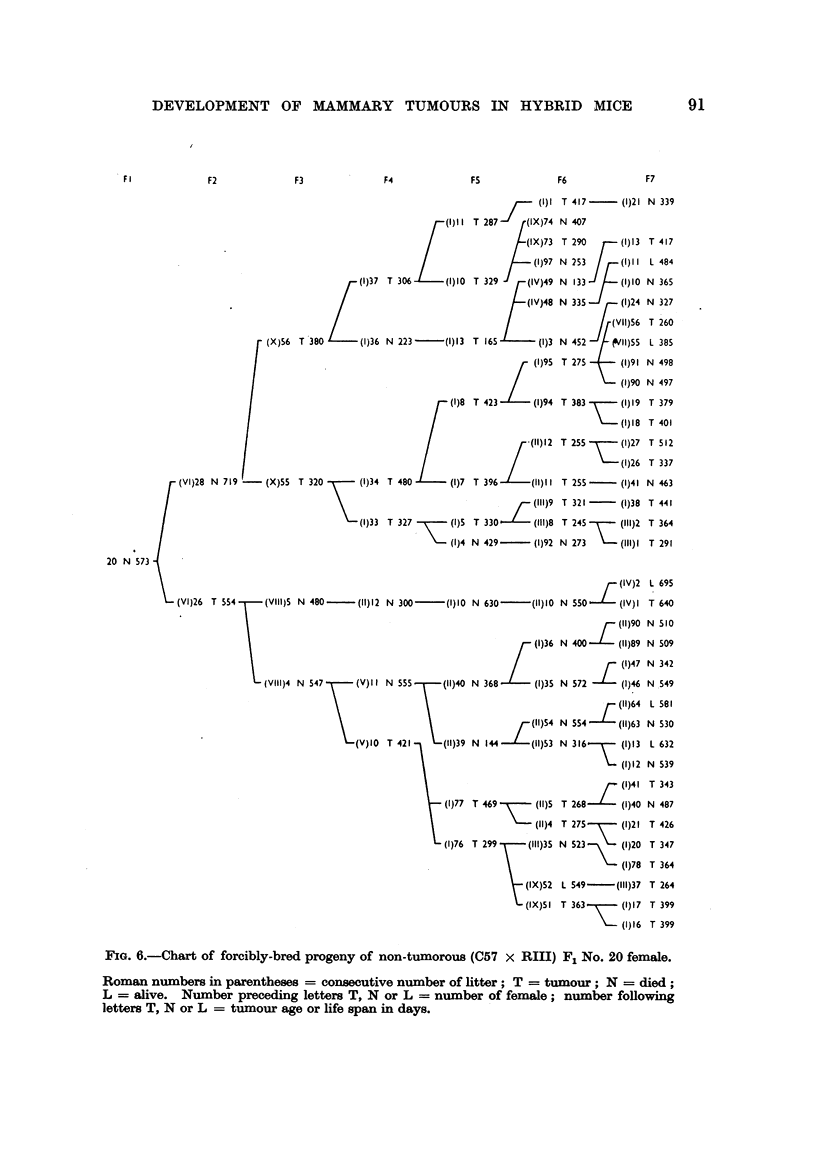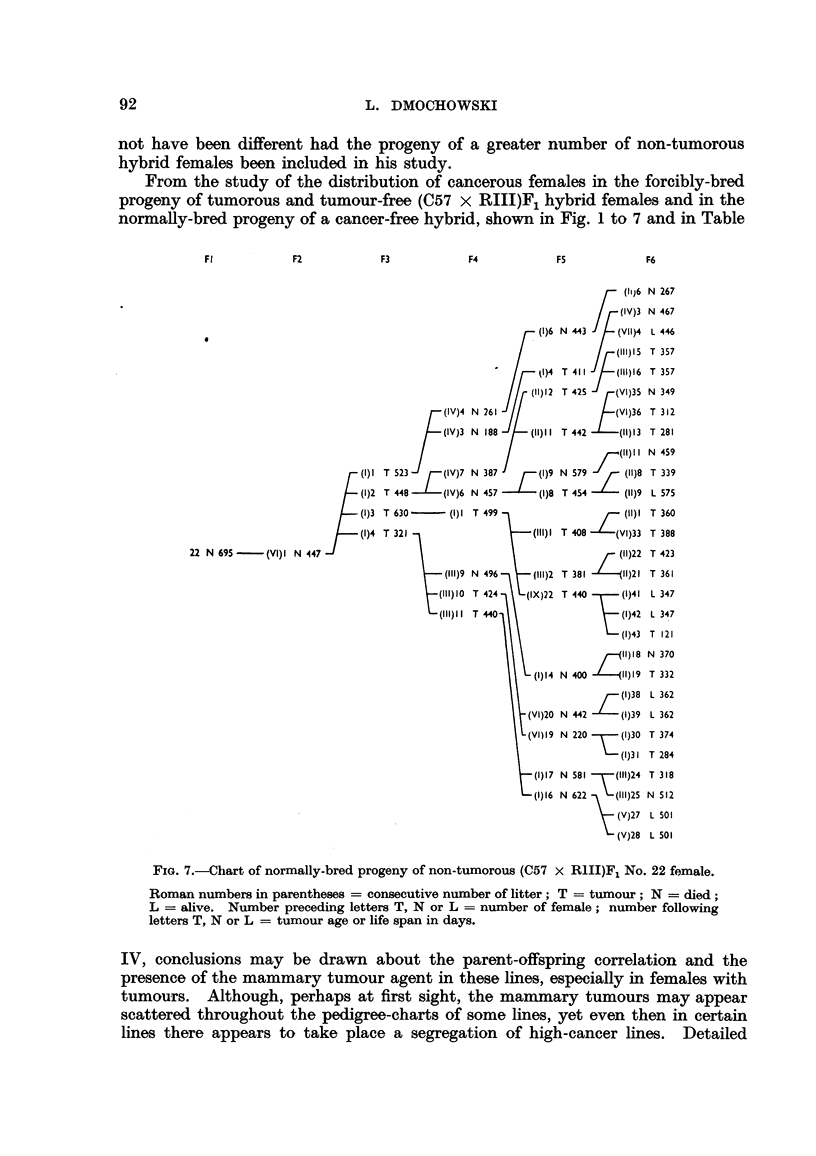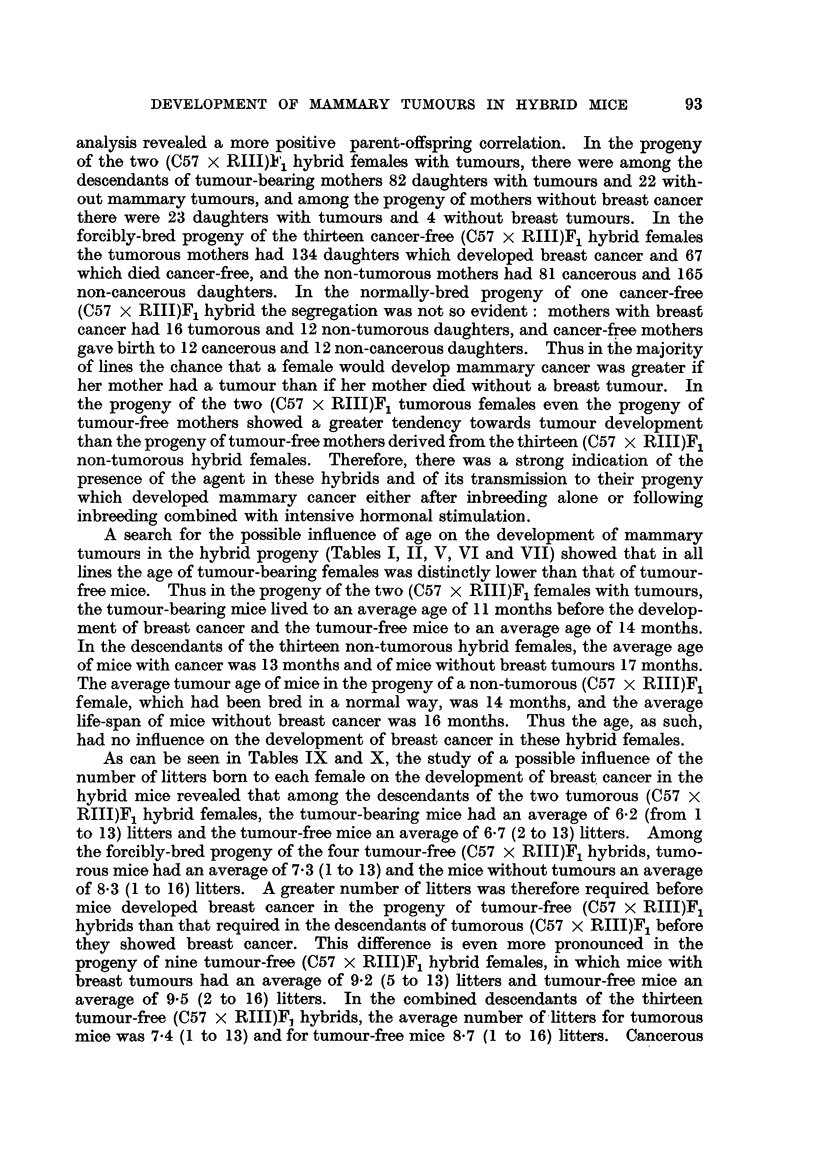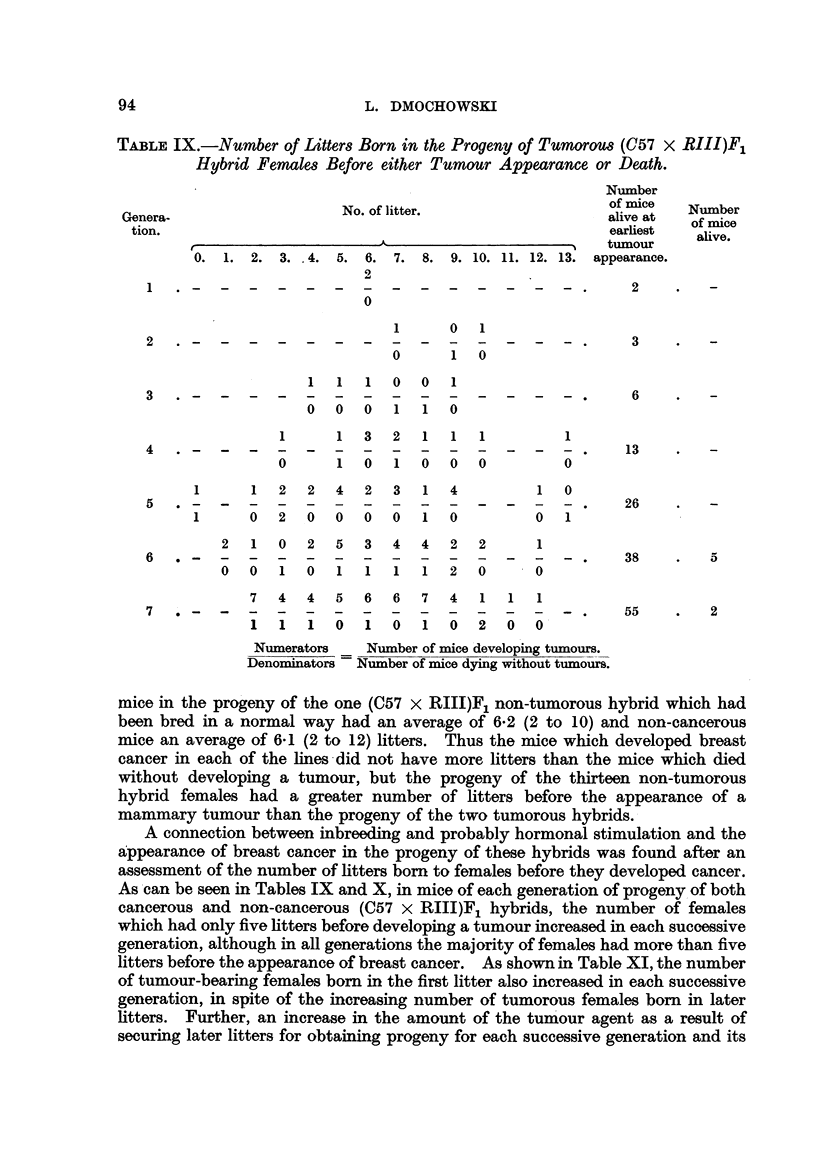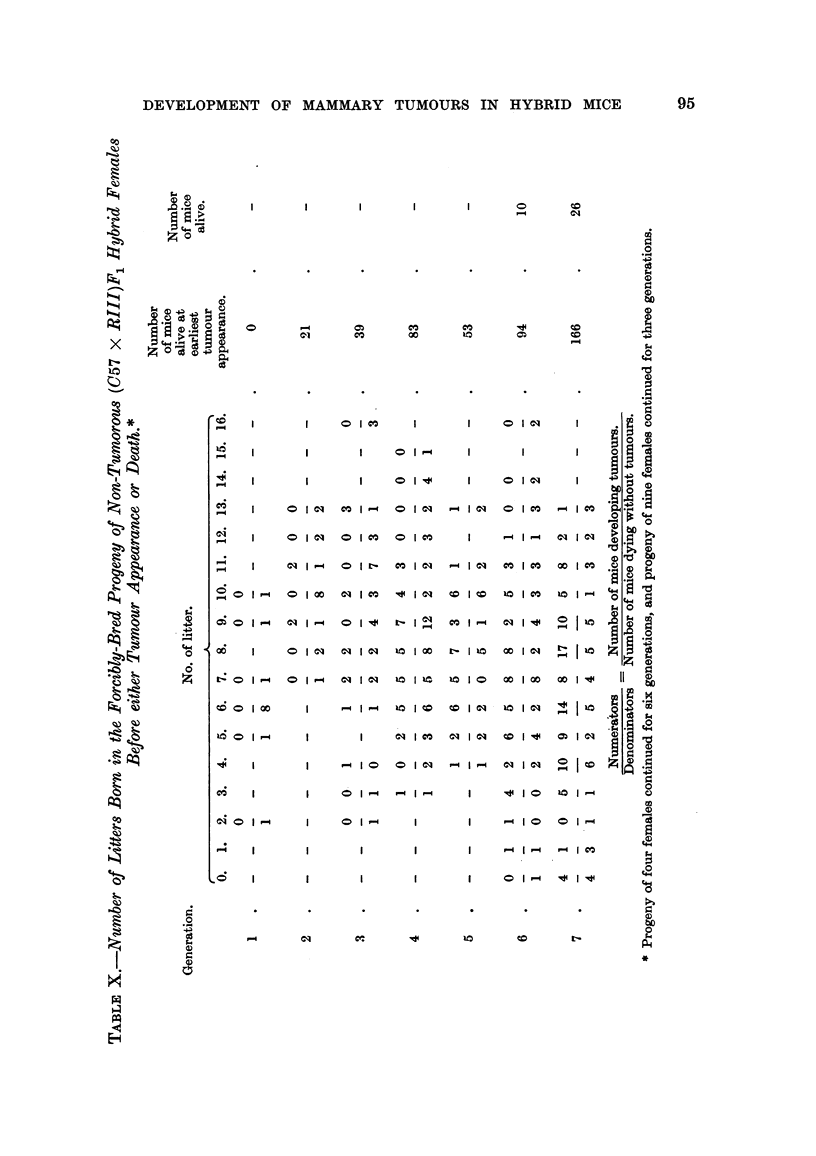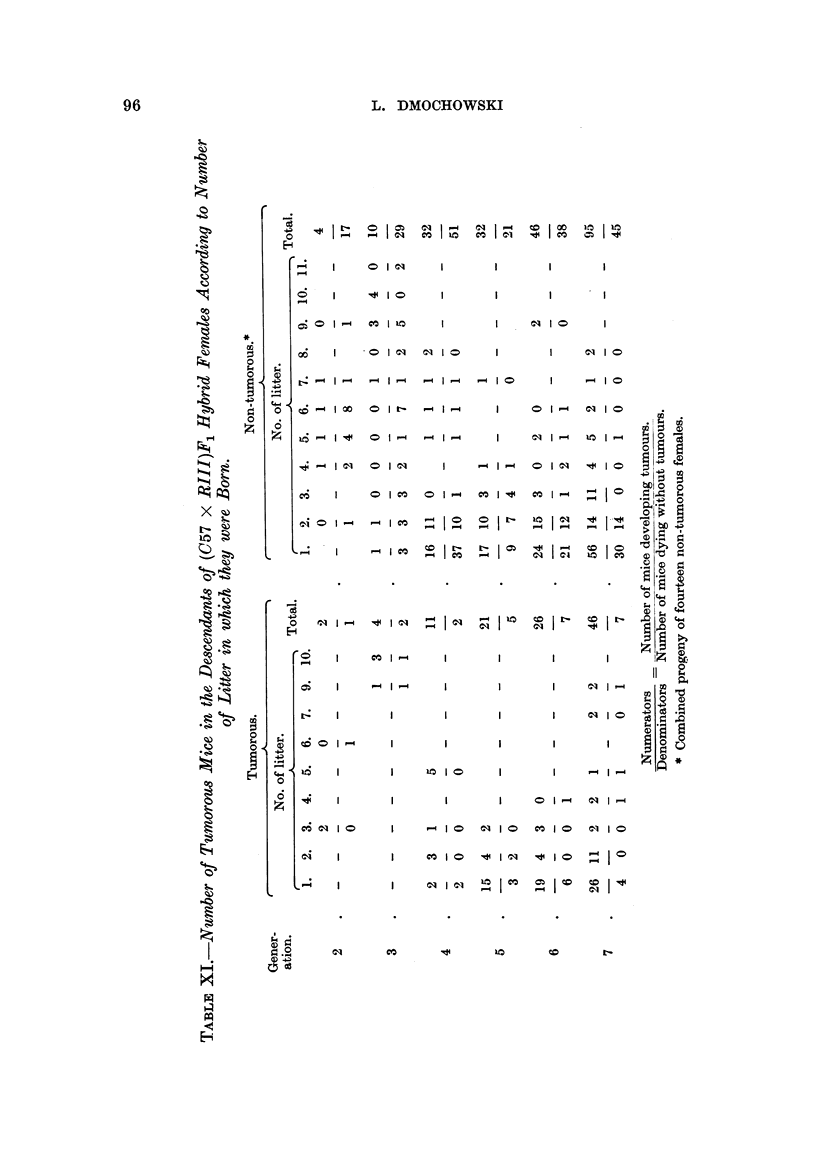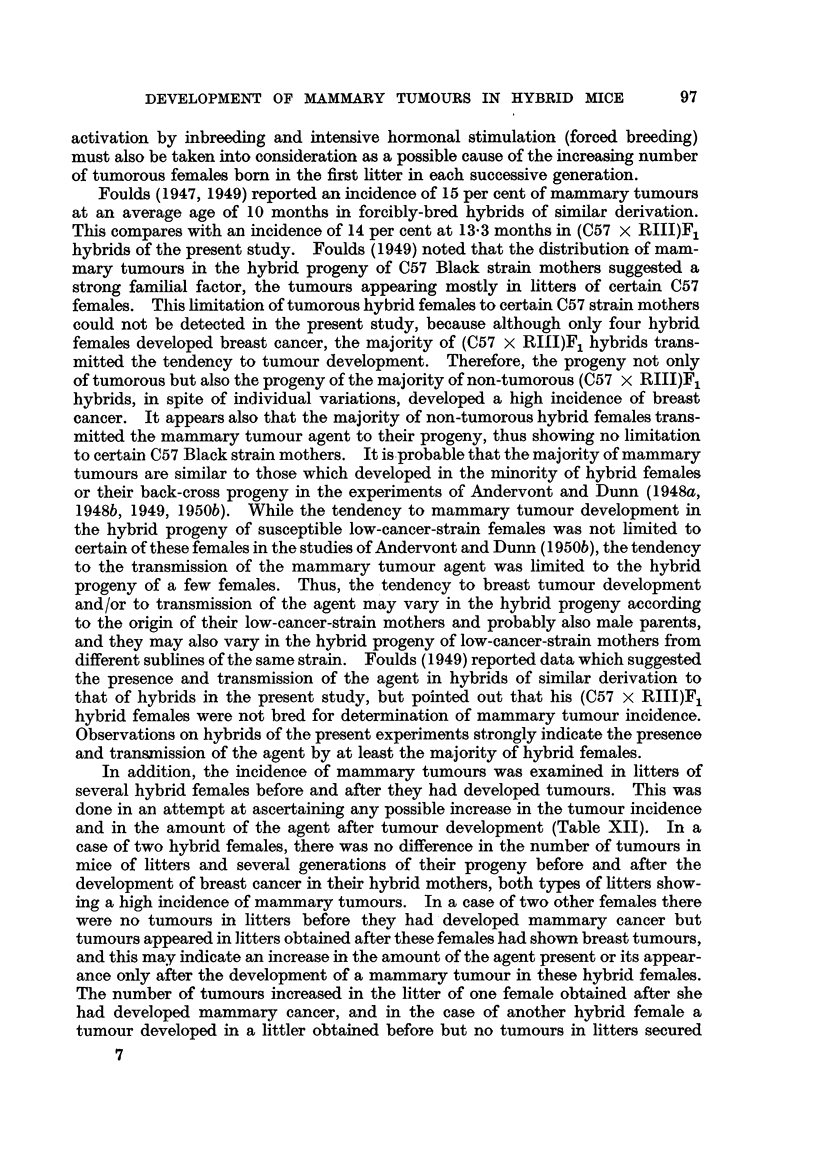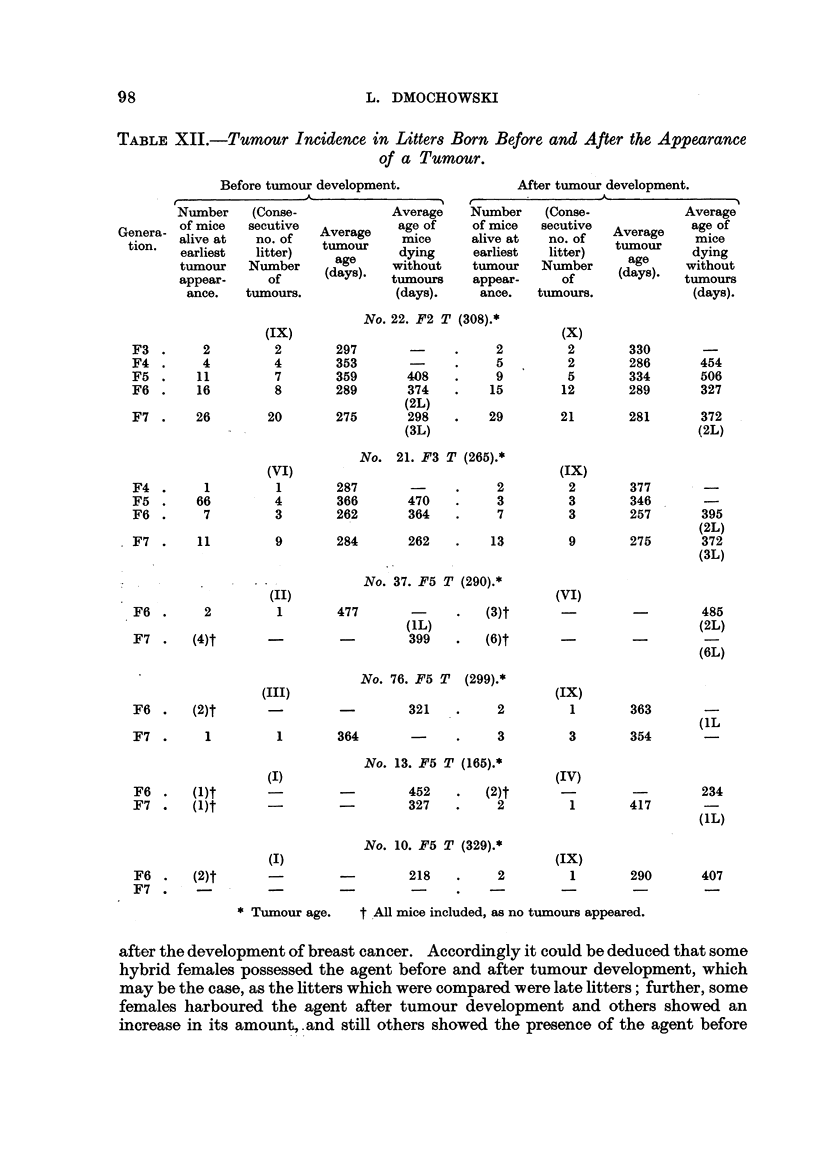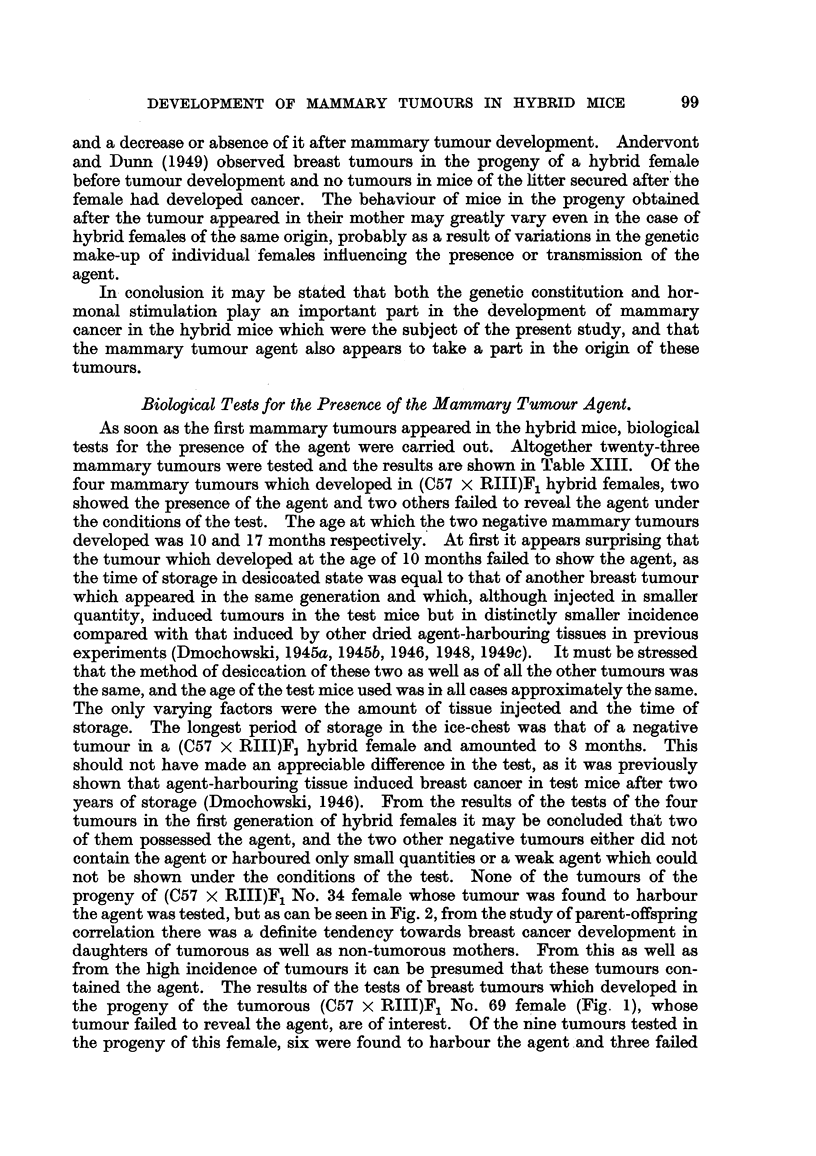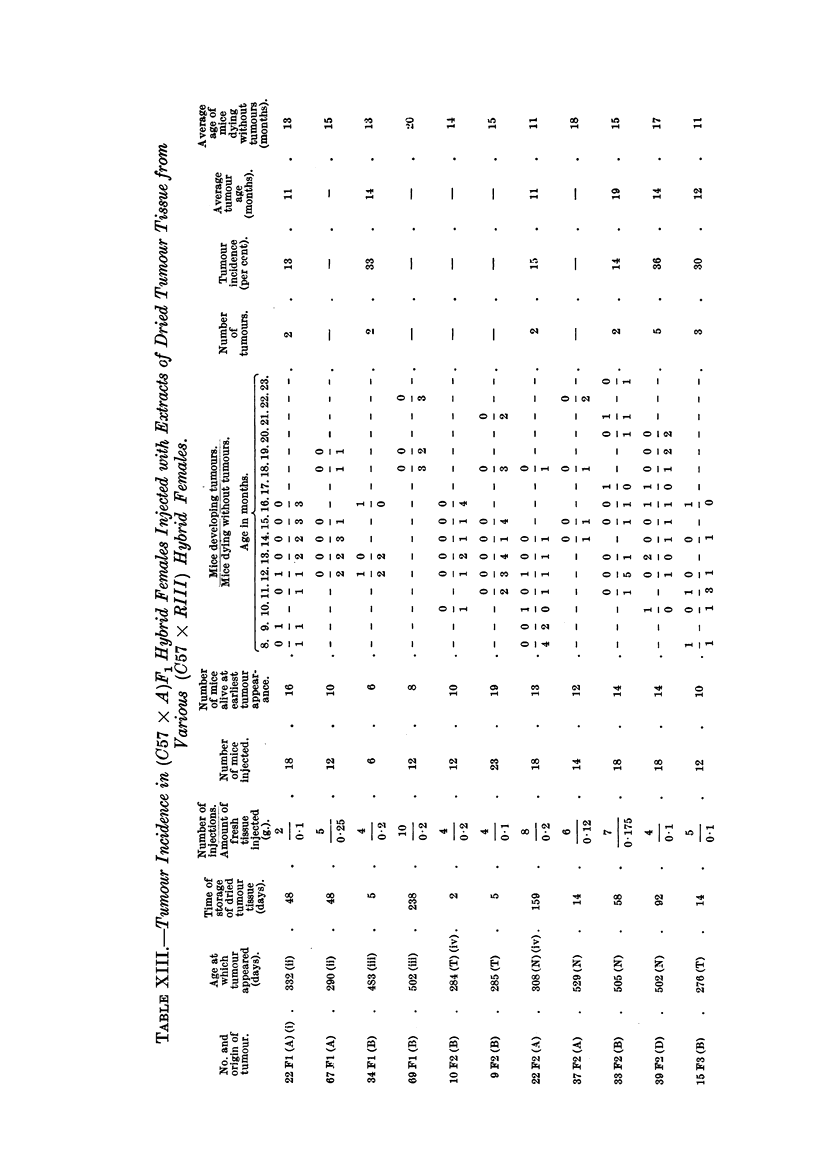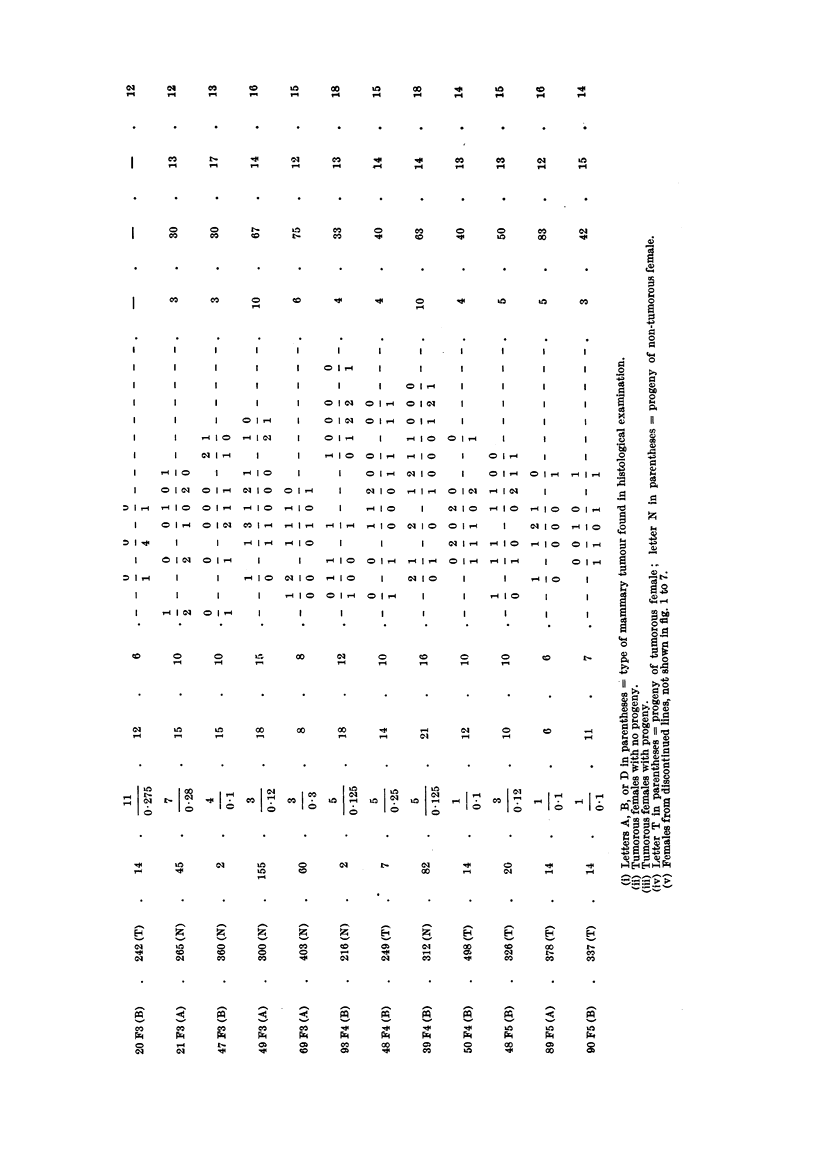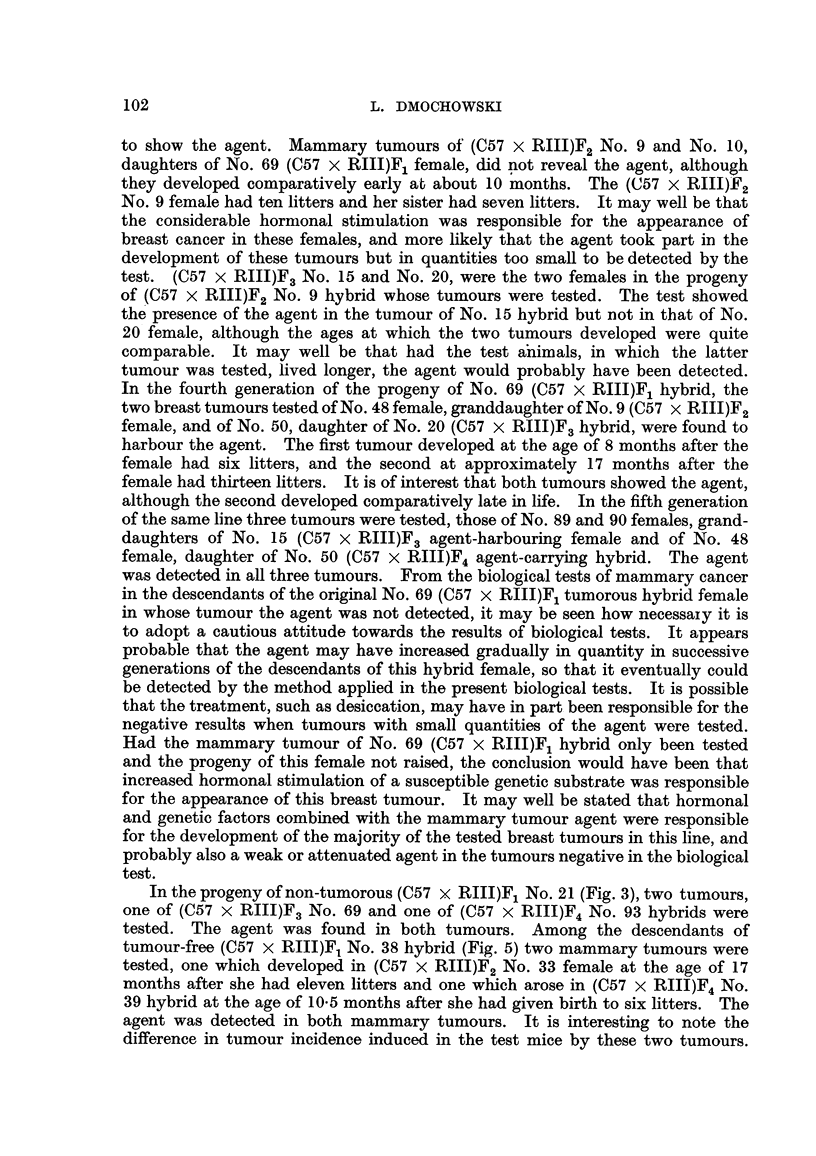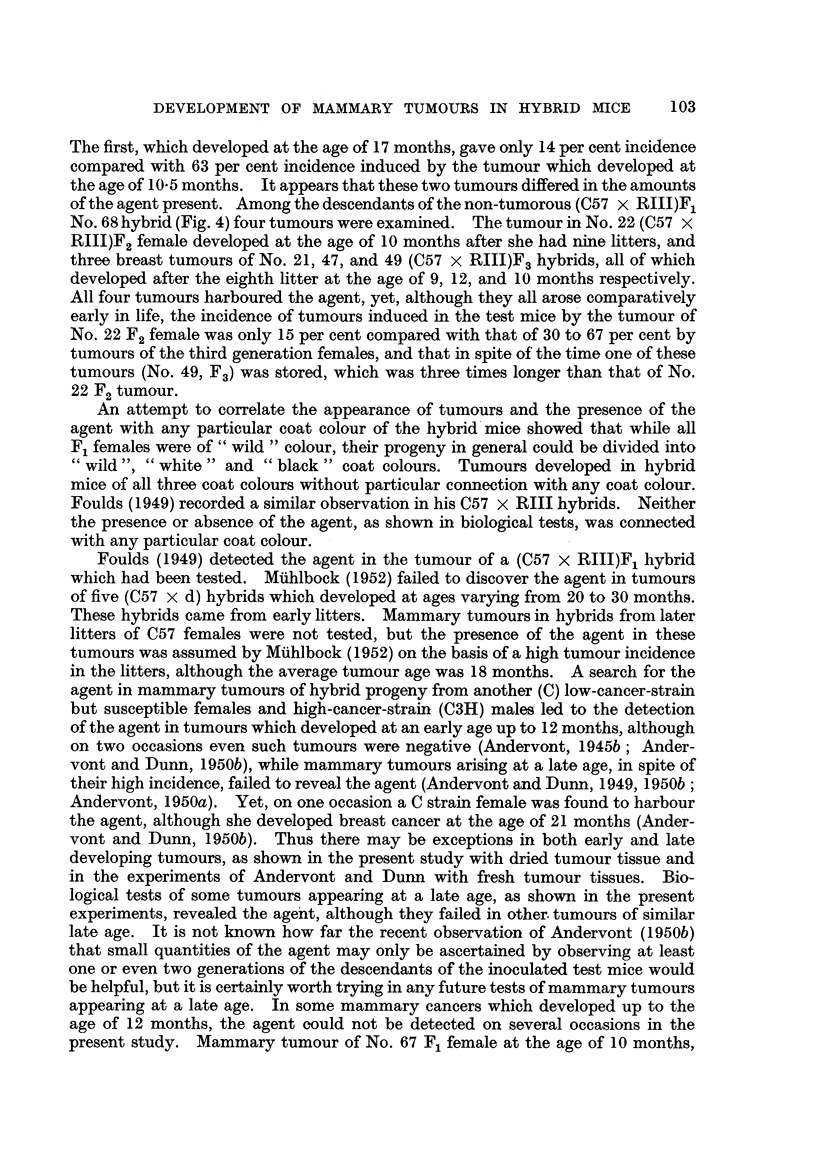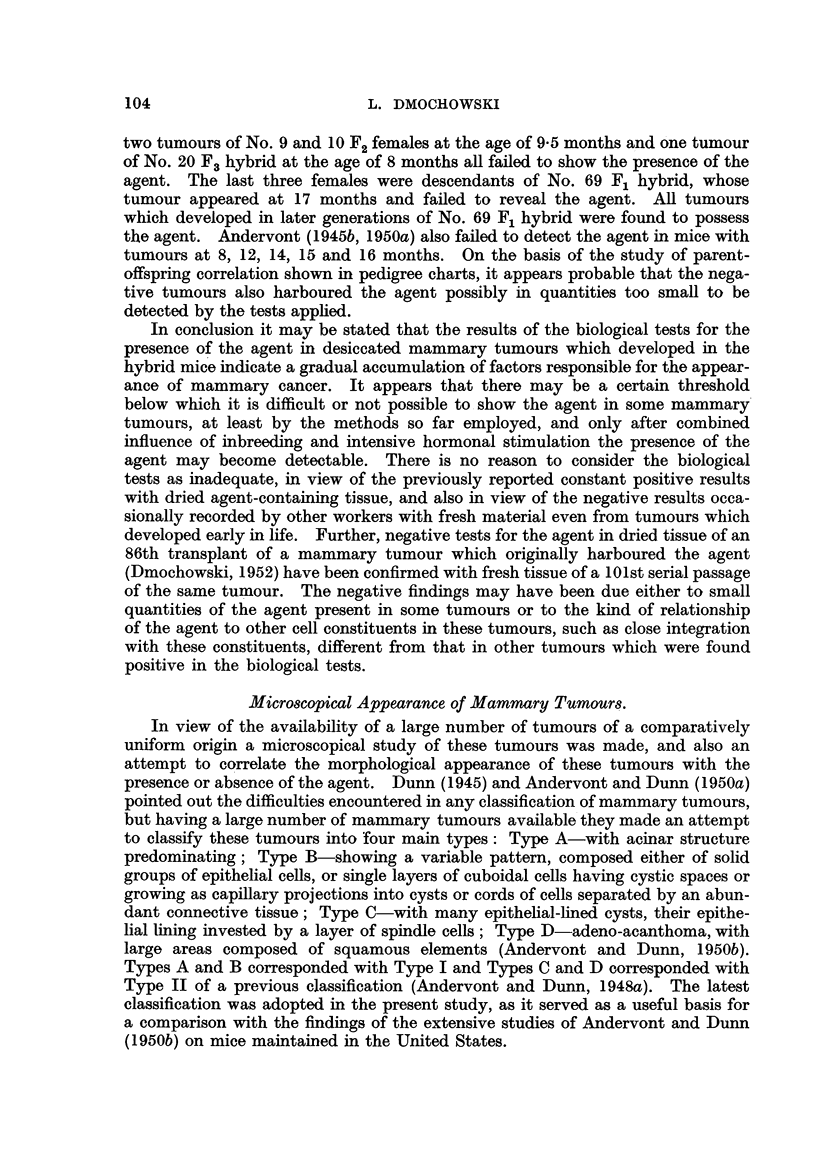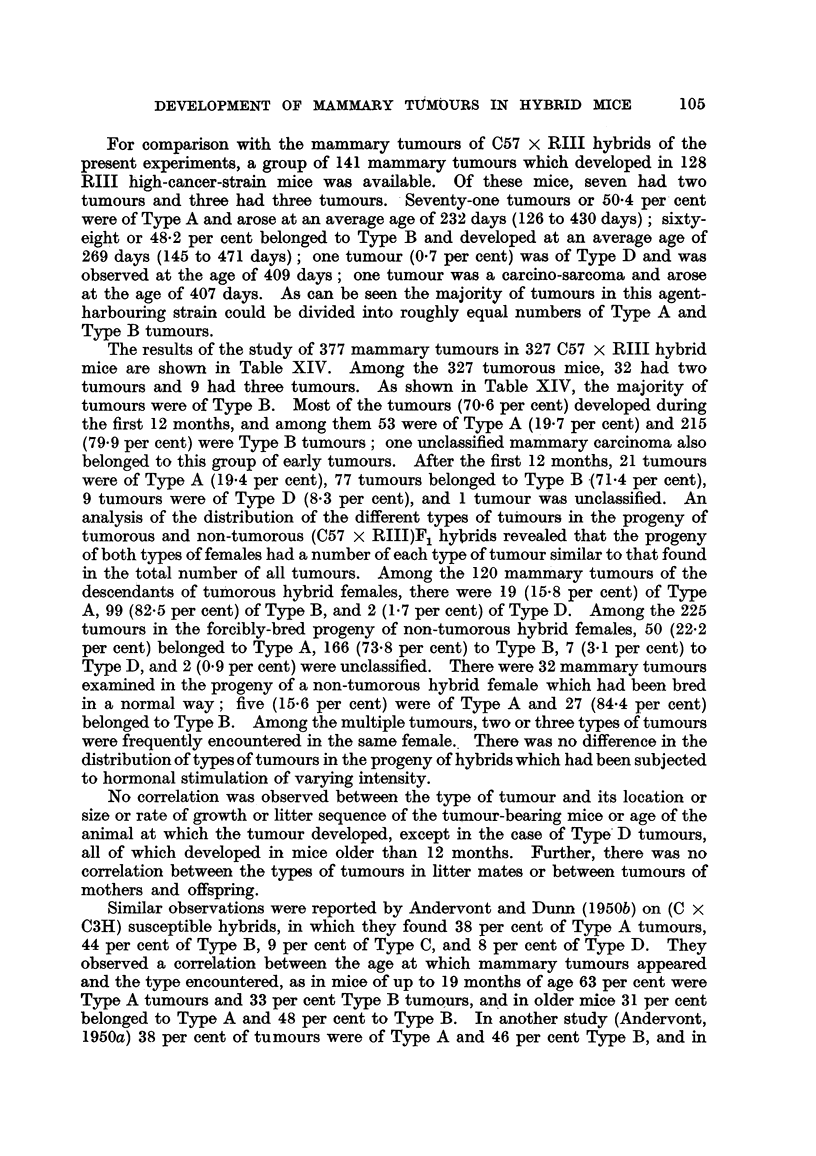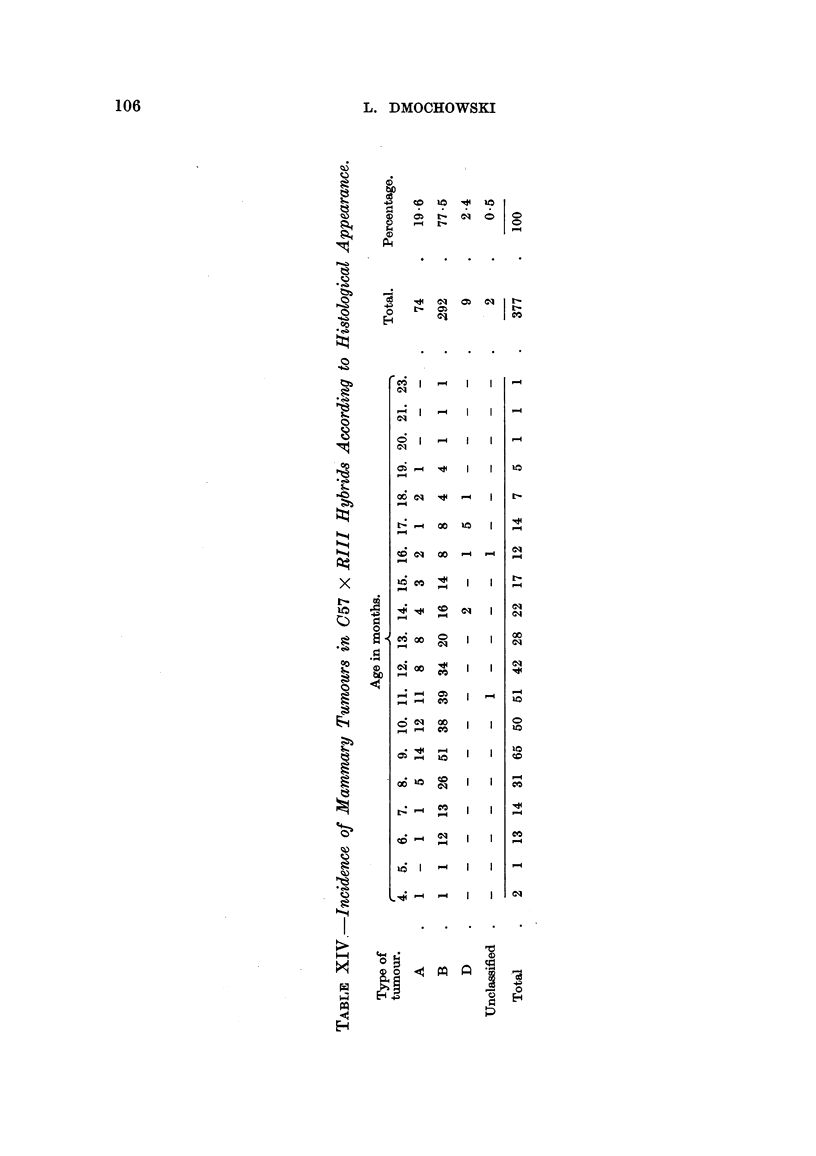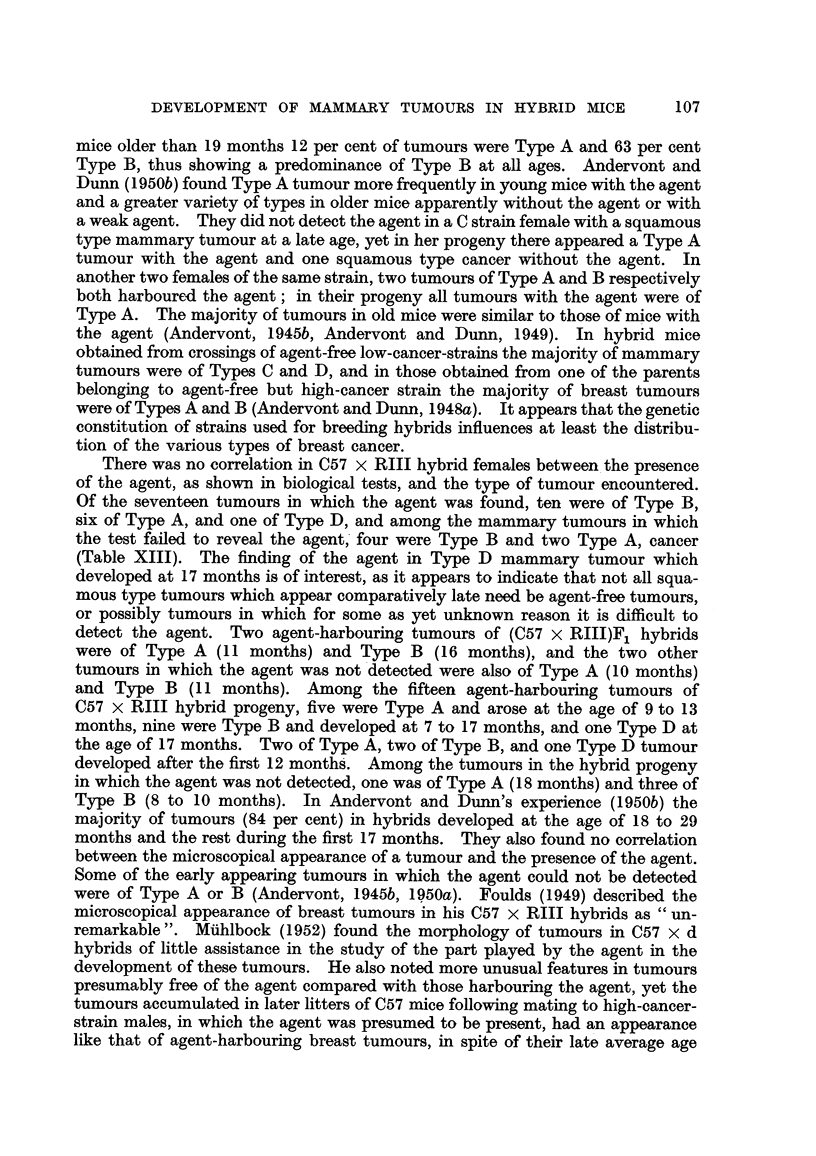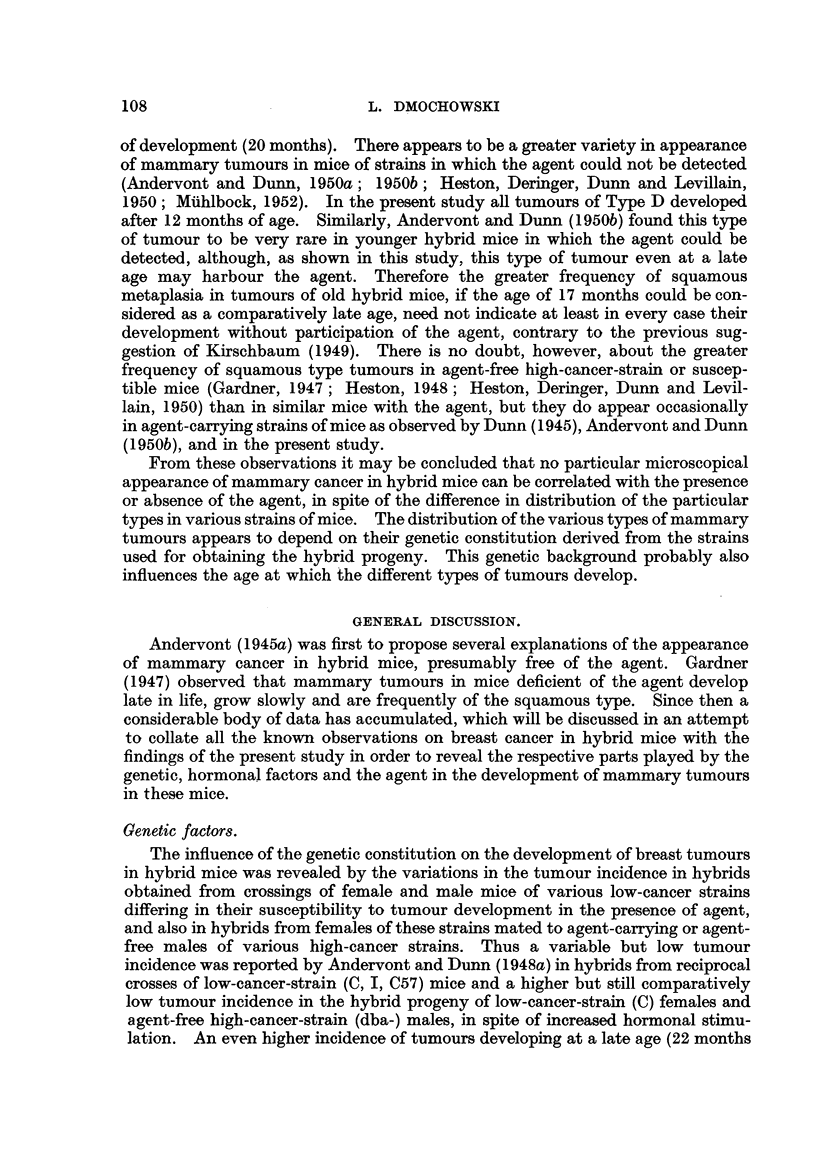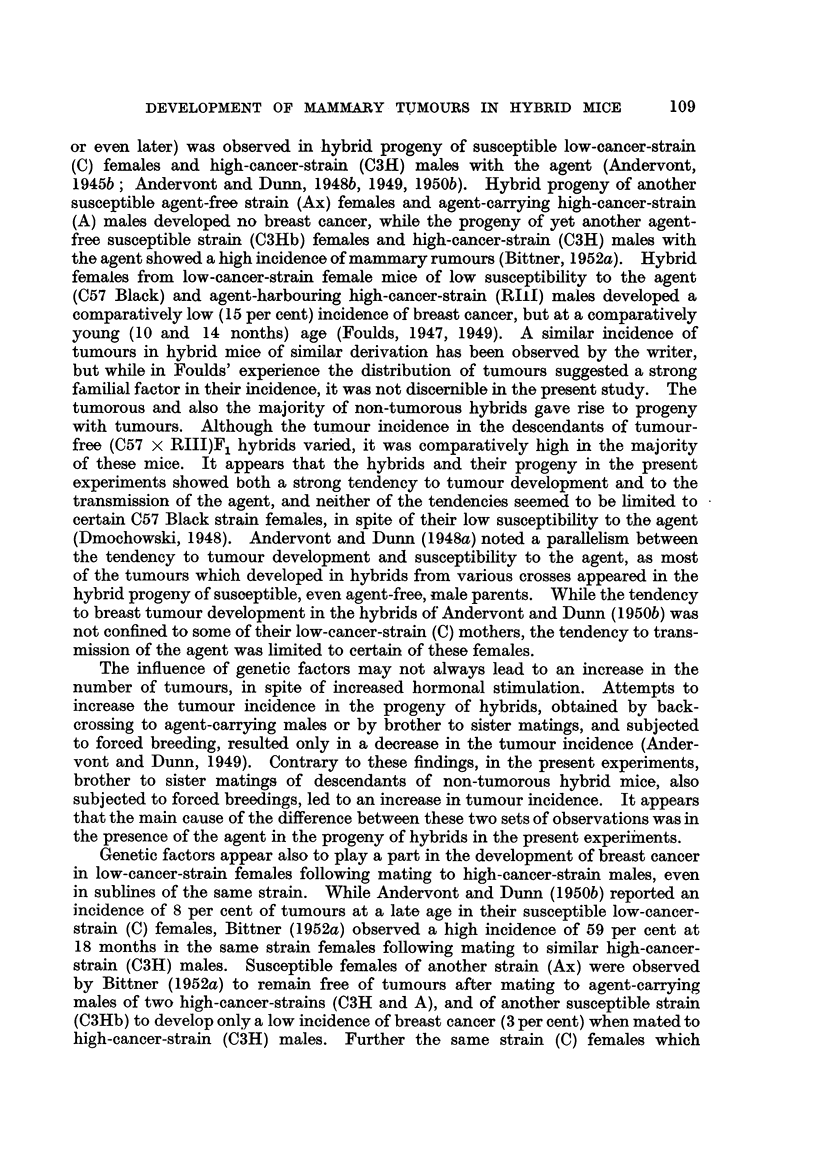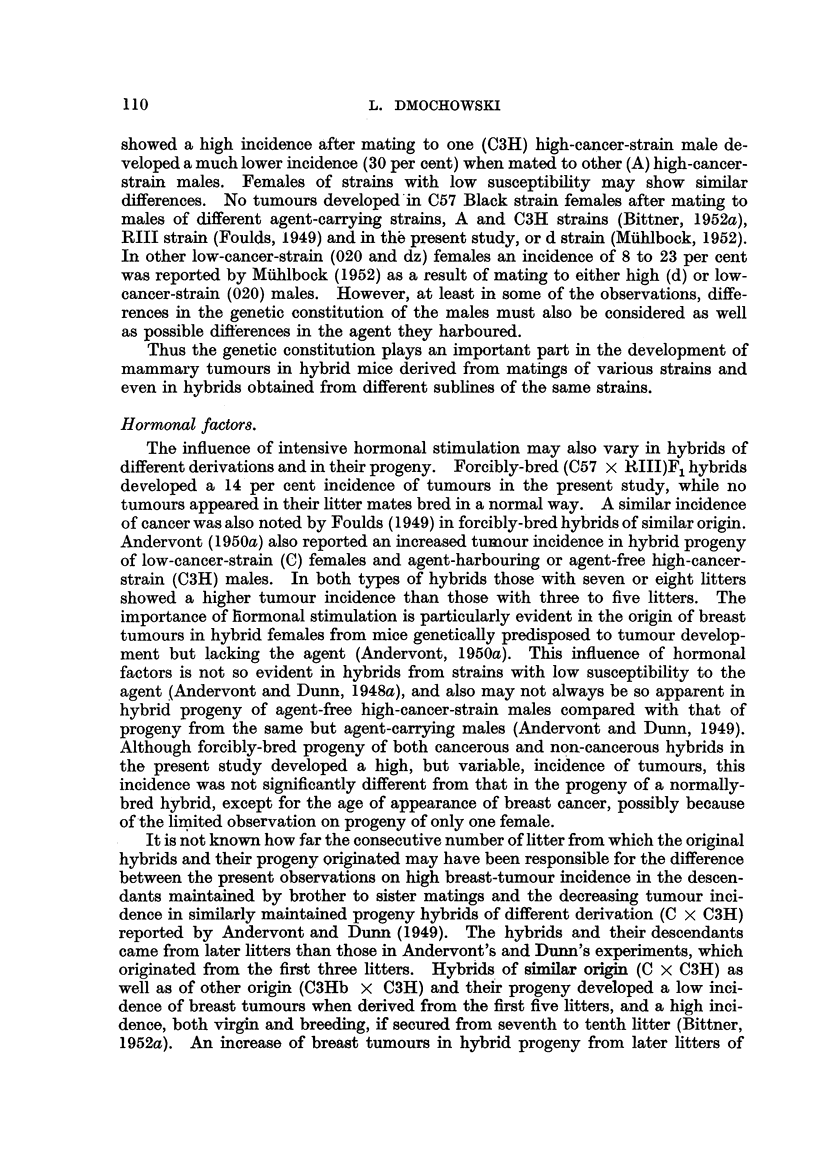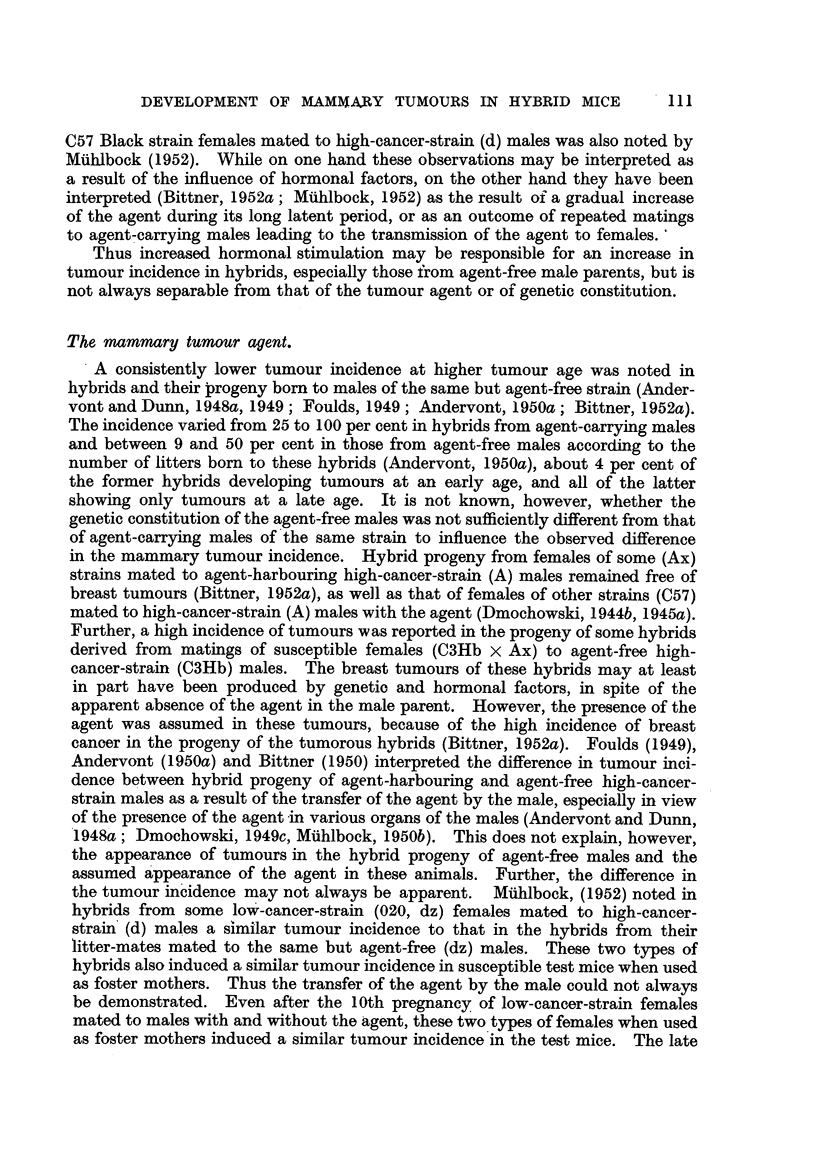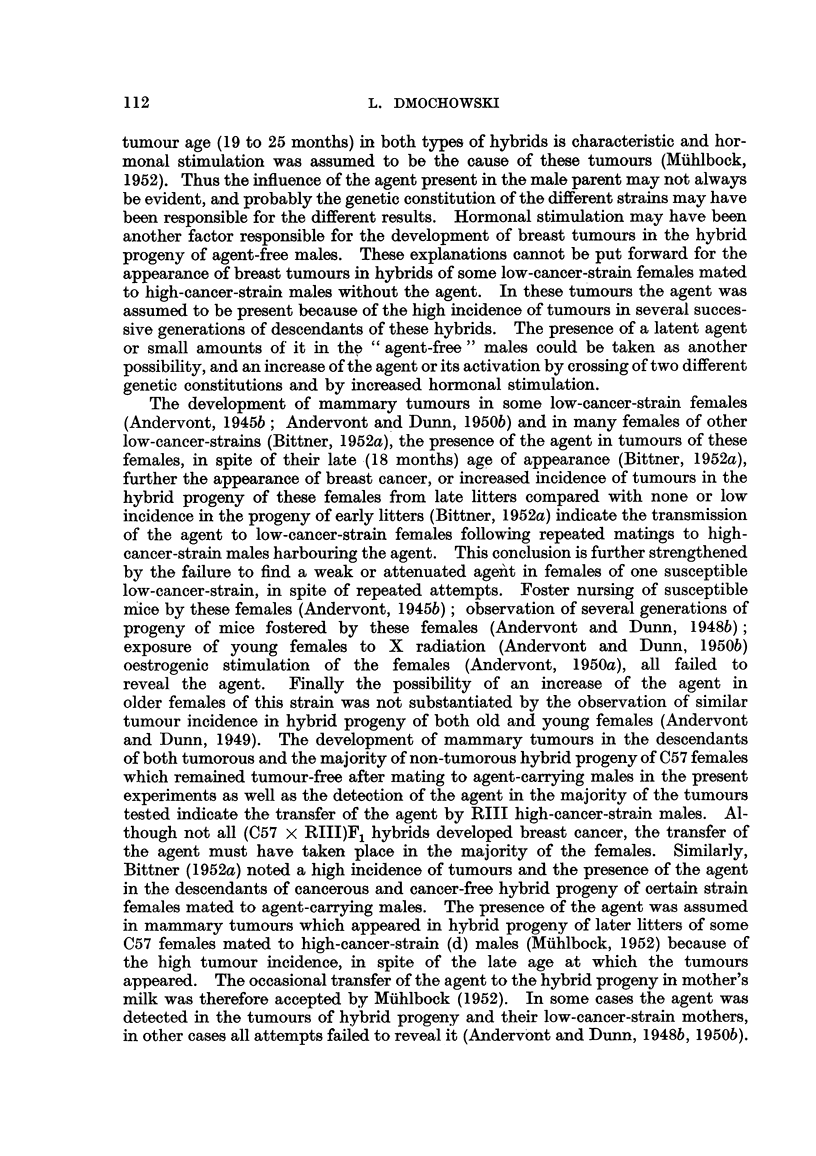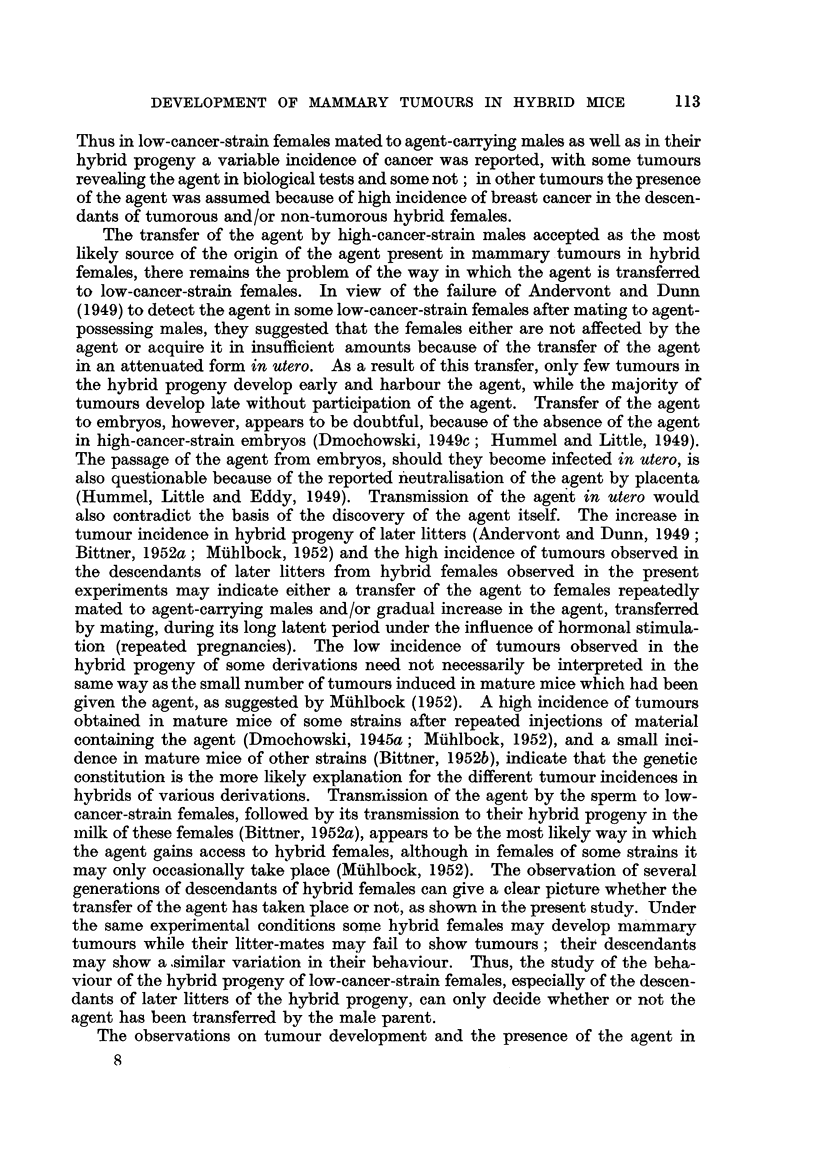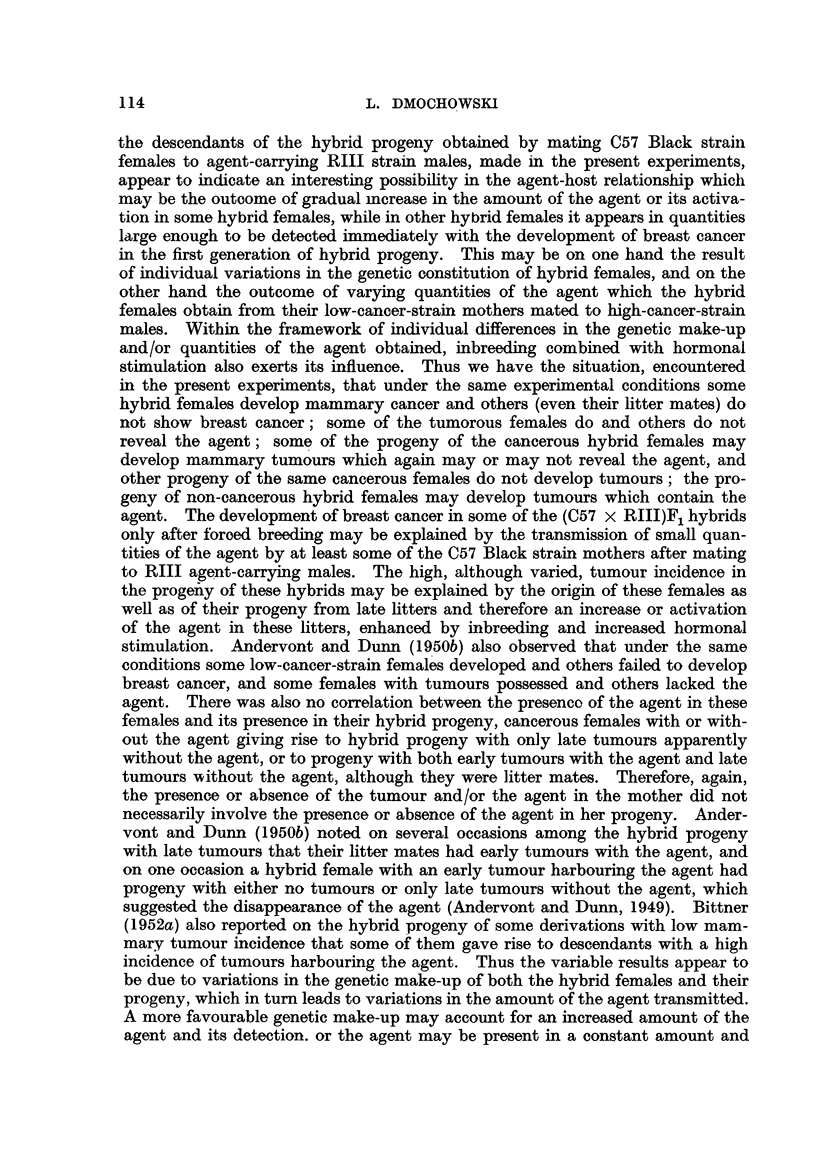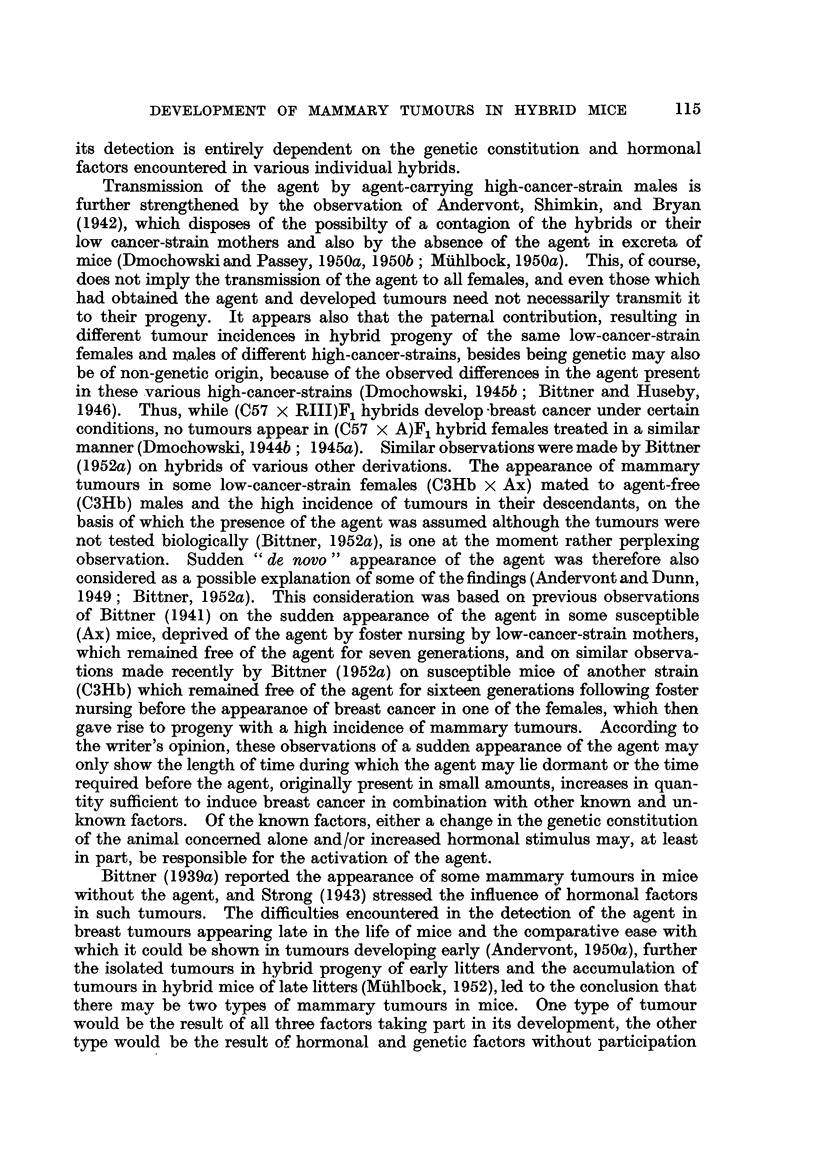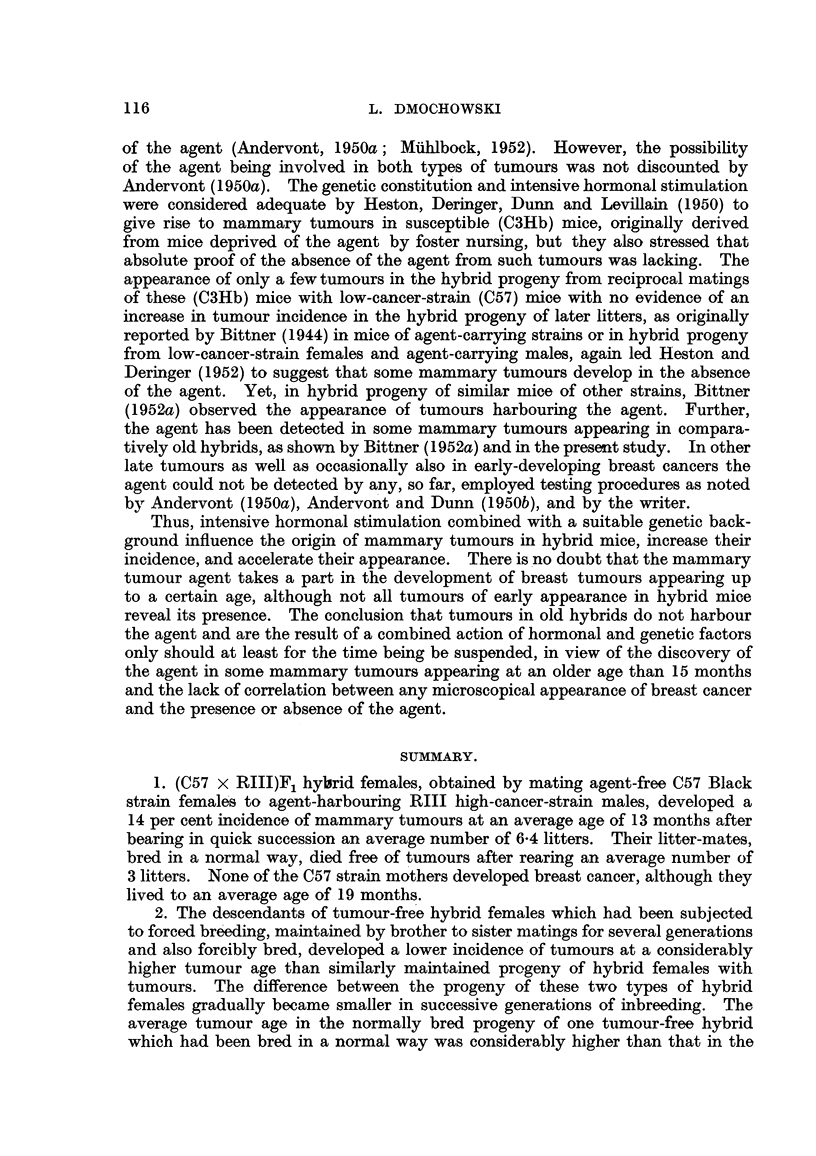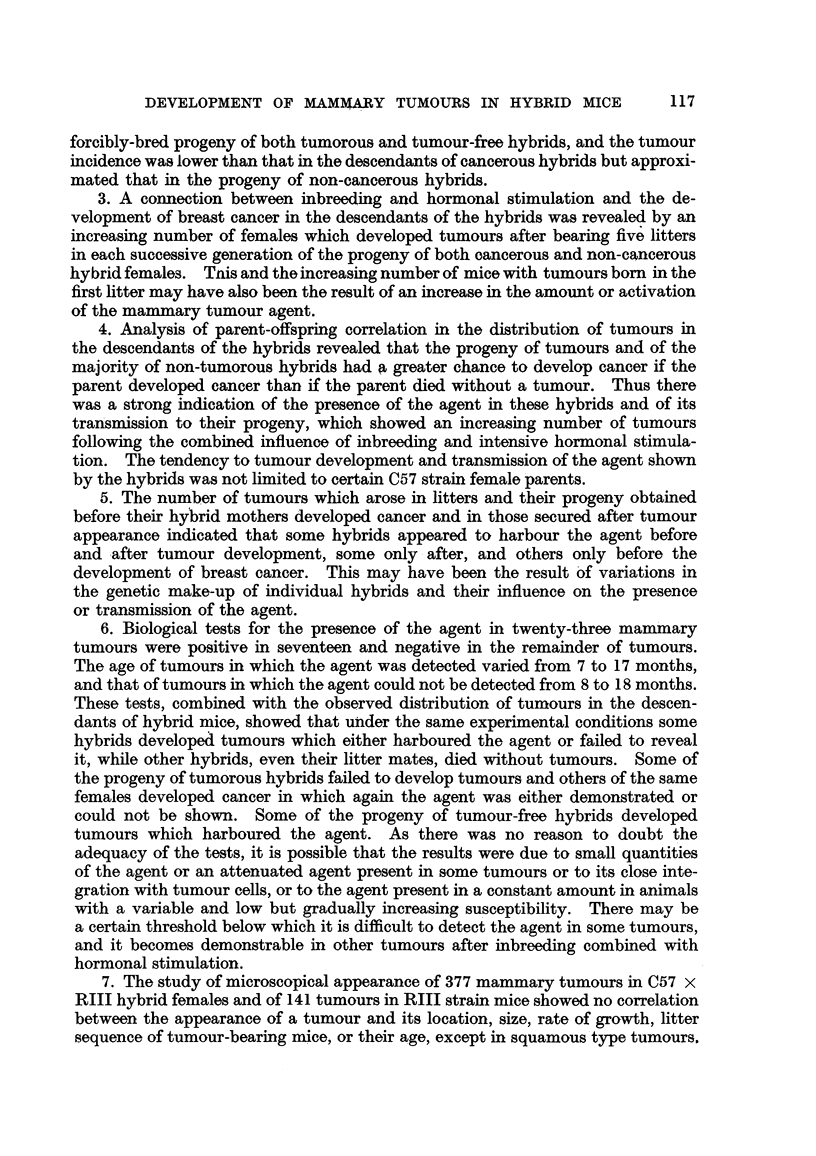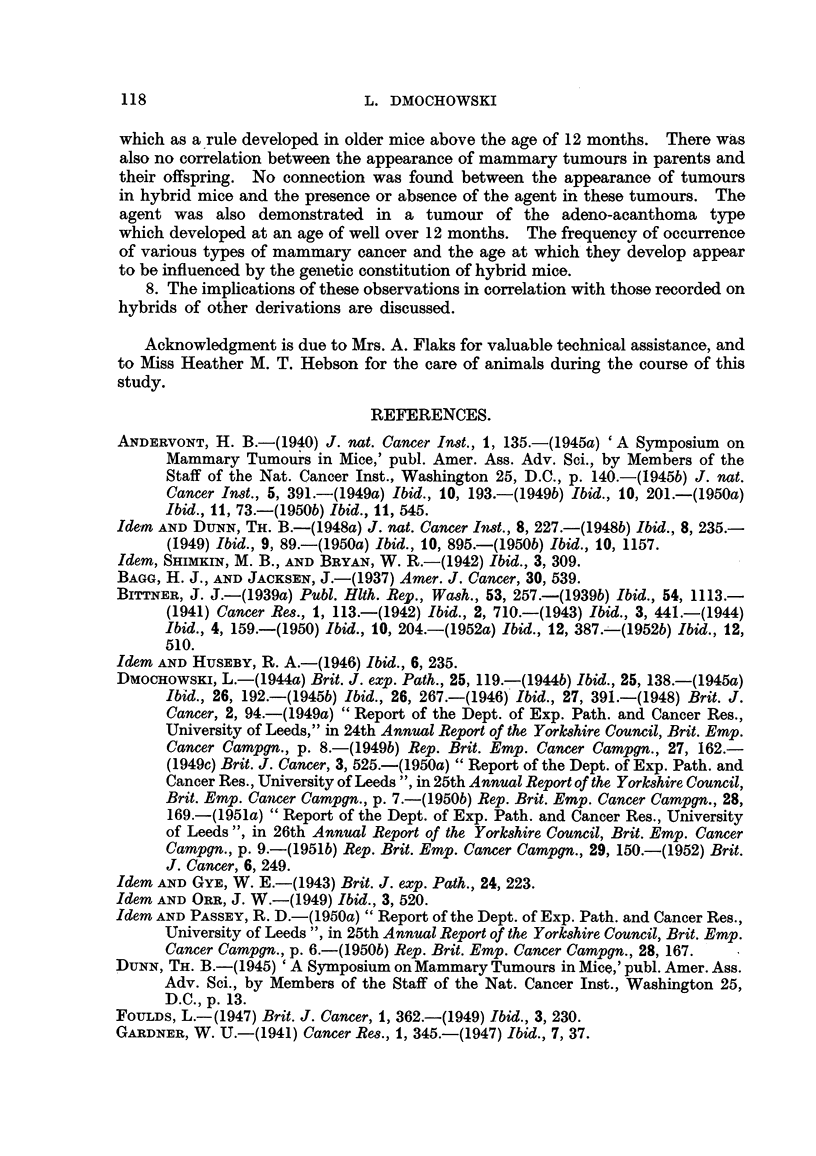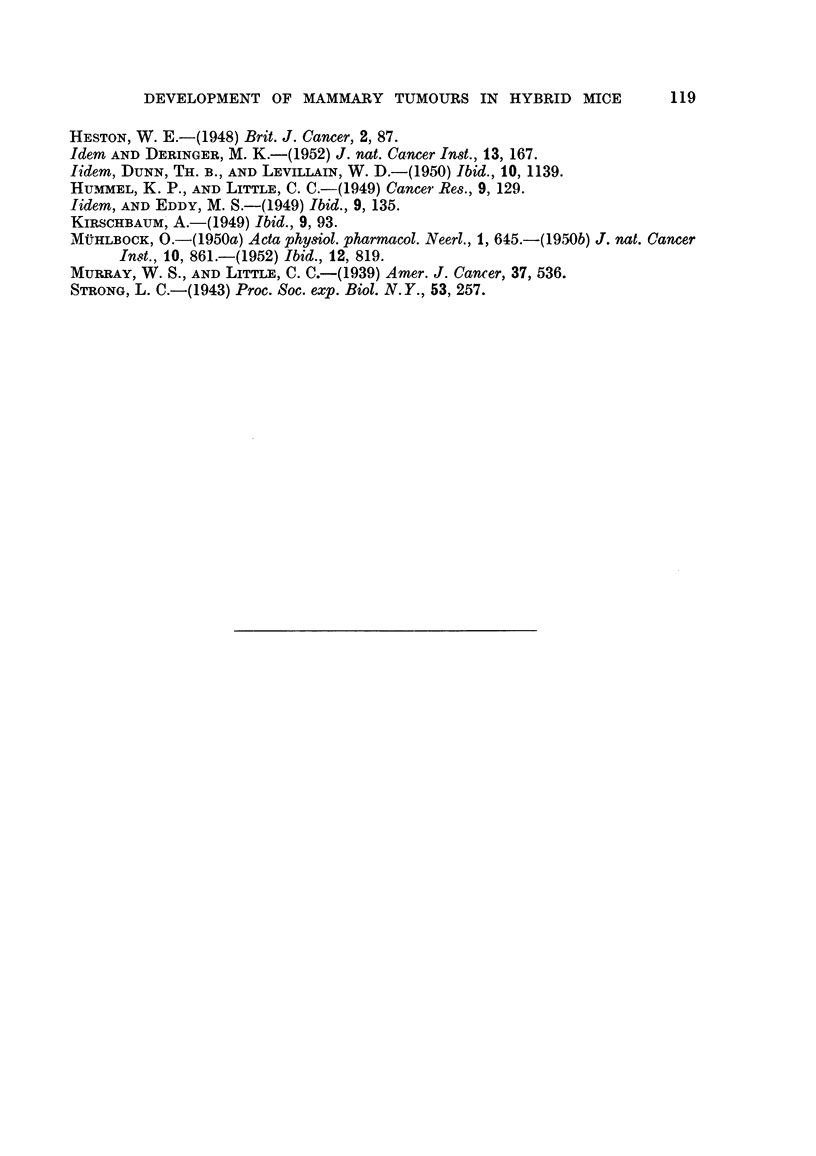# A Study of the Development of Mammary Tumours in Hybrid Mice

**DOI:** 10.1038/bjc.1953.9

**Published:** 1953-03

**Authors:** L. Dmochowski


### A STUDY OF THE DEVELOPMENT OF MAMMARY TUMOURS IN HYBRID MICE.

L. DMOCHOWSKI.

*From the Department of Experimental Pathology and Cancer Research, Medical School, University of Leeds.*

Received for publication December 15, 1952.

THE test mice most commonly used for ascertaining the presence of the mammary tumour agent are susceptible hybrid female mice obtained by mating low-breast-cancer-strain females to high-breast-cancer-strain males. In these test mice a low incidence of spontaneous mammary tumours was frequently recorded (Bittner, 1939*b* ; Murray and Little, 1939 ; Andervont, 1940 ; Gardner, 1941 ; Dmochowski, 1944*a*), and was frequently found to be higher than that of their maternal parents (Andervont, 1945*a*). Of even greater interest was the high tumour incidence reported in the progeny of susceptible low-cancer-strain females mated to high-cancer-strain males which had been subjected to forced breeding—that is, bearing a number of litters in quick succession (Andervont, 1945*b*). A similar high incidence of breast cancer was also observed in forcibly-bred hybrid progeny of low-cancer-strain females of low susceptibility to breast tumours mated to high-cancer-strain males (Bagg and Jacksen, 1937 ; Strong, 1943). Attempts to discover the mammary tumour agent in mammary tumours of some of these hybrids proved unsuccessful (Andervont, 1945*b*). A number of possibilities was suggested (Andervont and Dunn, 1949) to explain the appearance of breast cancer in hybrid females : presence of a weak agent or small quantities of it or even a different agent in low-cancer-strain female mice ; transmission of the agent from the male parent or influence of the more susceptible genetic constitution of this parent ; hormonal stimulation overcoming the absence or small quantities of the agent ; contamination ; even *de novo* origin of the agent.

In connection with the initially recorded observations on tumours in hybrid mice, an investigation was carried out in an attempt to obtain a high incidence of mammary tumours in hybrid mice bred in the laboratory, with a view to ascertaining the respective parts played by genetic constitution, hormonal stimulation, and the agent in the development of mammary cancer in these mice, obtained by mating C57 Black low-cancer-strain females of low susceptibility to the agent to RIII agent-harbouring high-cancer-strain males. It was also planned to study the inheritance of mammary tumours in the progeny of these hybrid females obtained by brother-to-sister matings.

Preliminary findings of this study have already been reported (Dmochowski, 1949*a*, 1949*b*, 1950*a*, 1950*b*, 1951*a*, 1951*b*). Biological tests for the presence of the agent in some of these tumours as well as a study of the microscopical appearance of breast cancer in these mice are now complete, and a full account of this investigation is now possible. During the intervening years, since the present experiments were started, the problem of the origin of breast tumours in hybrid mice of various derivations has also engaged the attention of several other investigators (Foulds, 1947, 1949; Andervont and Dunn, 1948*a*, 1948*b*, 1949, 1950*b*; Andervont, 1950*a*; Bittner, 1952*a*; Mühlbock, 1952). Therefore, a comparison of the observations in the present study with those of other investigators will also be presented.

### MATERIAL AND METHODS.

The experiments were started in October, 1947, by establishing a breeding colony of thirty C57 Black low-cancer-strain females and ten RIII high-cancer-strain males. The C57 Black strain mice originated from the Laboratories of the Imperial Cancer Research Fund and have been under the writer's care since 1946. Spontaneous mammary tumours have not been recorded in this strain since its establishment in the Imperial Cancer Research Fund Laboratories in 1941. The ancestors of the C57 Black strain females used in the experiments were all free of mammary tumours. A subline of C57 Black strain is now established in which a number of mammary tumours has been recorded by the author. This subline comes from one of the original three litters brought from the Laboratories of the Imperial Cancer Research Fund. The progeny of these litters has been kept separately, and no breast tumours have so far been recorded in the descendants of the other two litters. The description of the subline in which tumours have appeared will be the subject of a separate communication. The RIII strain mice were also obtained from the same source and maintained since 1946. The tumour incidence of 83 per cent at an average age of 8·5 months in breeding females (Dmochowski and Gye, 1943) has with small variations remained approximately the same. It was known that mice of high-cancer strains derived from the fourth or later litters develop a higher incidence of breast cancer than those from the first two litters (Bittner, 1942), and that the appearance of an active agent in mice deprived of it by foster nursing had been recorded in the progeny obtained from the third or later litters (Bittner, 1943). Hybrid progeny of the C57 Black strain females was therefore taken only from the fourth or later litters for the experiment, mice from earlier litters being used for various other purposes. The (C57 × RIII)$F_1$ hybrid females which were litter mates were divided into two groups. Only two hybrid females were taken from each of the C57 females mated to RIII strain males, if possible from the same or following litters. Whenever two females were not available from the same litter, the mice from later litters were included in the control group. In this way two groups, each comprising thirty (C57 × RIII)$F_1$ hybrid females, were obtained. Mice of the control group were allowed to breed in a normal way and mice of the experimental group were forcibly bred by removing their litters as soon as they were born. Mice of one of the later litters from fifteen hybrid females in this group and of one litter of a female from the control group were saved for brother to sister matings. These mice in turn, after saving one litter, were forcibly bred and this procedure was followed for several generations. The progeny of six (C57 × RIII)$F_1$ hybrid females, which had been subjected to forced breeding, was maintained for six generations and the progeny of nine additional (C57 × RIII)$F_1$ hybrids, treated in a similar manner, for only three generations. The descendants of a (C57 × RIII)$F_1$ hybrid female, which had been normally bred, were maintained by normal breeding for five generations.

In addition a number of litters from several C57 × RIII hybrid females was secured after the development of a mammary tumour, in an attempt to ascertain any difference in the tumour incidence and the presence of the agent in mice born before and after their mothers developed a tumour. The progeny of mice born before and after the development of breast cancer was also kept for observation of any difference in the incidence of mammary tumours.

Biological tests of twenty-three breast tumours which developed in (C57 × RIII)$F_1$ hybrid females and in their progeny were carried out for the presence of the mammary tumour agent. The tumours, after securing a part for histological examination, were frozen and desiccated as previously described (Dmochowski, 1946). After storage in the ice-chest for varying periods of time according to the availability of test mice, the desiccated tumour tissue was ground in a mortar and resuspended in distilled water in a proportion of 1 : 10, and then usually further diluted to a volume according to the number of test mice available, so as to give daily each animal 1 ml. of extract intraperitoneally. The number of injections and the amount of material given at each injection varied according to the total amount of tissue available. The intention always was to give as much material as was available. (C57 × A)$F_1$ hybrid females, 4 to 5 weeks old, were used as test mice. All test mice were forcibly bred by removing the first three litters within 24 hours of birth, and then allowed to breed in a normal way. Spontaneous mammary tumours have not been observed in (C57 × A)$F_1$ hybrid females after similar treatment (Dmochowski, 1944*b*, 1945*a*), and the total tumour incidence in all these mice, so far used, is below 3 per cent. The test mice as well as all other mice were maintained under similar conditions on a diet of " rat-cake " cubes, biscuits, and oats, and given an unlimited supply of tap-water. Intermittent outbreaks of enteritis during the later part of the experiment were successfully controlled by streptomycin supplied in a final concentration of 0·025 per cent in drinking water. The number of animals kept in one cage was six in the case of the test mice, and from two to four in the case of all other mice.

Histological examination of all tumours, whenever possible, was carried out. Tumours which were not examined microscopically were excluded from the present study. The tissues were fixed in Bouin's fluid and stained with haematoxylin and eosin.

### RESULTS.

No mammary tumours were observed in any of the (C57 × RIII)$F_1$ hybrid females which had been bred in a normal way, although they lived to an average age of approximately 19 months (Table I). One of the females developed a

TABLE I.—*Tumour Incidence in Normally-Bred (C57 × RIII)$F_1$ Hybrid Females.*

| Number of mice alive at earliest tumour appearance. | Earliest tumour appearance in forcibly bred hybrids (days). | Mice developing tumours. / Mice dying without tumours. Age in months. 12. | 14. | 16. | 17. | 18. | 19. | 20. | 22. | 23. | 26. | Number of tumours. | Number of mice dying without tumours. | Average age of mice dying without tumours (days). |
|---|---|---|---|---|---|---|---|---|---|---|---|---|---|---|
| | | 0 | 0 | 0 | 0 | 0 | 0 | 0 | 0 | 0 | 0 | | | |
| 29 | 290 | – | – | – | – | – | – | – | – | – | – | 0 | 29 | 559 |
| | | 1 | 1 | 8 | 1 | 5 | 3 | 2 | 4 | 2 | 2 | | | (372–794) |

tumour which on histological examination was found to be a lymphosarcoma. The average number of litters born to these hybrid females was three, and varied from two to seven. The number of breast tumours which developed in their litter mate (C57 × RIII)$F_1$ hybrid females, which had been forcibly bred, is shown in Table II. The number of litters born to these hybrids varied from four to ten and the average number was 6·4. Among these hybrids, the tumorous females had an average of 6·5 litters and the tumour-free females 6·4 litters. There was no difference therefore in the number of litters born to these two types of females which had been forcibly bred.

None of the C57 Black strain mothers, mated to RIII strain males and bred in a normal way, developed mammary cancer, although they lived to an average age of 19 mouths. Twelve C57 Black strain females, which were litter-mates of some of the C57 females mated to RIII strain males, were forcibly bred with their own litter-mate males, and none developed breast cancer, although they had an average of 5·2 litters and lived to an average age of 18 months. At the same time a number of other low-cancer-strain female mice, ten C strain, supplied by Dr. H. B. Andervont, ten JK strain, supplied by Dr. L. C. Strong, eleven P strain and five Y strain, supplied by Dr. C. C. Little, were also forcibly bred with their own strain males. In none of these females was mammary cancer observed. Their average life-span with the average number of litters born shown in parentheses was: 19 months (7·4), 20 months (8·2), 18 months (5·4), and 17 months (5·6) respectively.

The appearance of mammary tumours in some of the (C57 × RIII)$F_1$ hybrid females following forced breeding could be explained by the transmission of small amounts of the agent by their C57 Black strain mothers after mating to RIII strain males, as originally suggested by Foulds (1949), although none of the C57 females developed breast cancer, and further by activation of the agent in their susceptible hybrid progeny by increased hormonal stimulation. The observation that none of the C57 strain females developed mammary tumours after mating to C57 strain males and forced breeding points against the possibility of C57 Black strain females harbouring a weak or attenuated mammary tumour agent. It is now well known that various sublines of C57 Black strain differ considerably in their susceptibility to the agent. C57 strain mice of the subline of the present experiments, when foster nursed by RIII strain females, developed an incidence of mammary cancer of only 11 per cent (Dmochowski, 1948). This low susceptibility was probably responsible for the failure to induce mammary cancer in mature C57 mice of this subline even by a combined action of large doses of material containing the agent and forced breeding (Dmochowski, 1948). Again this low susceptibility may also have been responsible for C57 mice of this subline remaining tumour-free after mating to RIII high-cancer-strain males as well as for their hybrid progeny, bred in a normal way, not developing mammary cancer. A further indication against the possibility of C57 Black mice of this subline harbouring a weak agent or in small quantities was provided by the absence of the agent in mammary tumours induced in both virgin and breeding C57 females by treatment with methylcholanthrene (Dmochowski and Orr, 1949). At least it can be stated that even treatment with methylcholanthrene combined with breeding failed to reveal either a weak agent or a strong agent on a genetic background of low susceptibility in C57 Black mice of the subline used in the present study.

TABLE II.—*Tumour Incidence in Forcibly-Bred (C57 × RIII)$F_1$ Hybrid Females.*

| Number of mice alive at the earliest tumour appearance. | Earliest tumour appearance. | Mice developing tumours / Mice dying without tumours. Age in months: 10. | 11. | 13. | 15. | 16. | 17. | 18. | 19. | 20. | 21. | 22. | 25. | Number of tumours. | Tumour incidence (%). | Average tumour age (days). | Number of mice died without tumours. | Average age of mice dying without tumours (days). |
|---|---|---|---|---|---|---|---|---|---|---|---|---|---|---|---|---|---|---|
| 29 | 290 | 1/1 | 1/1 | 0/2 | 0/2 | 1/4 | 1/3 | 0/3 | 0/2 | 0/1 | 0/1 | 0/4 | 0/1 | 4 | 13·8 | 402 | 25 | 526 |

TABLE III.—*Appearance of Tumours in Forcibly-Bred Progeny of Individual Tumorous and Non-Tumorous (C57 × RIII)$F_1$ Hybrid Females.*

| F1. No. of female with tumour (age of tumour development, days). | F1. No. of female without tumour (age at death, days). | F2. a. | F2. b. | F2. c. | F2. d. | F2. e. | F3. a. | F3. b. | F3. c. | F3. d. | F3. e. | F4. a. | F4. b. | F4. c. | F4. d. | F4. e. | F5. a. | F5. b. | F5. c. | F5. d. | F5. e. | F6. a. | F6. b. | F6. c. | F6. d. | F6. e. | F7. a. | F7. b. | F7. c. | F7. d. | F7. e. |
|---|---|---|---|---|---|---|---|---|---|---|---|---|---|---|---|---|---|---|---|---|---|---|---|---|---|---|---|---|---|---|---|
| 34 (483) | — | — | 0/1 | — | — | 560 | 280 | 1/2 | 50 | 280 | 574 | 226 | 7/8 | 88 | 344 | 293 | 203 | 13/15 | 87 | 319 | 531 | 226 | 14/19 | 74 | 321 | 332 (5 alive) | 196 | 23/26 | 88 | 310 | 368 (2 alive) |
| 69 (502) | — | 284 | 2/2 | 100 | 285 | — | 246 | 3/4 | 75 | 282 | 396 | 248 | 4/5 | 80 | 328 | 619 | 326 | 8/11 | 73 | 398 | 333 | 239 | 12/14 | 86 | 330 | 504 | 125 | 23/27 | 85 | 279 | 265 |
| — | 21 (748) | — | 0/1 | — | — | 900 | 403 | 1/1 | 100 | 403 | — | 216 | 2/4 | 50 | 387 | 504 | 304 | 4/8 | 50 | 423 | 560 | 303 | 4/8 | 50 | 327 | 581 (3 alive) | 203 | 8/10 | 80 | 301 | 490 (7 alive) |
| — | 68 (376) | 308 | 1/1 | 100 | 308 | — | 265 | 4/4 | 100 | 314 | — | 301 | 6/10 | 60 | 331 | 412 | 225 | 12/20 | 60 | 348 | 457 | 184 | 20/29 | 69 | 289 | 358 (2 alive) | 143 | 41/50 | 82 | 278 | 348 (5 alive) |
| — | 20 (573) | 554 | 1/2 | 50 | 554 | 719 | 320 | 2/4 | 50 | 350 | 514 | 306 | 4/6 | 67 | 384 | 389 | 165 | 8/11 | 73 | 337 | 476 | 245 | 11/22 | 50 | 304 | 421 (1 alive) | 260 | 17/30 | 57 | 387 | 458 (5 alive) |
| | 38 (643) | 505 | 1/2 | 50 | 505 | 520 | — | 0/4 | — | — | 587 | 187 | 4/8 | 50 | 310 | 571 | 265 | 8/14 | 57 | 418 | 536 | 190 | 11/25 | 44 | 412 | 520 (4 alive) | 206 | 29/46 | 63 | 340 | 397 (9 alive) |

a = Earliest tumour appearance (days).

b = Number of tumours. / Number of mice alive at earliest tumour appearance.

c = Tumour incidence (per cent).

d = Average tumour age (days).

e = Average age at death without tumour (days).

In an attempt to increase, if possible, the mammary tumour incidence in the descendants of (C57 × RIII)$F_1$ hybrid females, the progeny of these mice was continued for several generations by brother to sister matings and subjected to having as many litters as possible, after saving at least one litter for the next generation. The progeny of two (C57 × RIIIF)$_1$ hybrid females which developed mammary cancer is shown in Fig. 1 and 2. The forcibly-bred progeny of four (C57 × RIII)$F_1$ hybrid females which had died free from tumours, also continued for six generations, is presented in Fig. 3 to 6. In these figures, the number of mammary tumours which appeared in each successive generation of both the tumorous and non-tumorous hybrids may be seen at a glance as well as the consecutive number of litter to which the mice with tumours belonged, the age at which they developed cancer or died without showing a tumour. It will also be noted that as a rule the hybrid females of the first three generations belonged to later litters than those of the following generations. This was done in an attempt to increase the activity of the mammary tumour agent, should one be present in the hybrid females. It may also be seen in Fig. 3 to 6 that descendants of tumorous females can remain tumour-free for two, three or four successive generations before developing mammary cancer, and that descendants of a non-tumorous female, in spite of reaching tumour age, may show no mammary tumours for as many as five generations before developing breast cancer, in spite of inbreeding and intensive hormonal stimulation. It may be of interest to point out that Andervont (1949*a*, 1949*b*) found the agent to be transmitted through three or four successive generations of susceptible mice without inducing mammary cancer. Further, the study of the distribution of mammary tumours in charts of the progeny of tumorous and non-tumorous hybrids, shown in Fig. 1 to 6, indicates the possibility of the agent being involved in the development of these tumours.

A comparison of the age of the earliest tumour appearance, tumour incidence, average tumour age and average age at death of the progeny of individual tumorous and four tumour-free (C57 × RIII)$F_1$ hybrid females is shown in Table III. As can be seen, there was little, if any, difference between the forcibly-bred progeny of the two (C57 × RIII)$F_1$ females which developed breast cancer and the forcibly-bred progeny of the four (C57 × RIII)$F_1$ hybrid females which died without tumours. Table IV demonstrates the tumour incidence and tumour age in the forcibly-bred progeny of additional nine non-tumorous (C57 × RIII)$F_1$ females which was continued for three generations. The tumour incidence, although varied in the progeny of the individual hybrids, was lower and the age of breast tumour appearance higher than those in the progeny of the two tumorous and also of the four tumour-free (C57 × RIII)$F_1$ hybrid females. The progeny of these nine non-tumorous hybrids showed considerable variations in tumour incidence and tumour age ; the descendants of two hybrids (No. 27 and 36) showed no tumours in the three generations observed ; those of four other hybrids (No. 31, 33, 21 and 72) did not develop tumours until the third generation of inbreeding ; the progeny of only one hybrid female (No. 40) developed mammary cancer in the second generation, although all of them had been subjected to considerable hormonal stimulation. In Table V is shown the tumour incidence, the age of appearance of the earliest tumour and the average tumour age in the six generations of the forcibly-bred progeny of the two cancerous (C57 × RIII)$F_1$ hybrid females. It can be seen that the tumour incidence in all six generations

TABLE IV.—*Appearance of Mammary Tumours in Forcibly-Bred Progeny of Individual Non-Tumorous (C57 × RIII)$F_1$ Females.*

| (C57 × RIII)$F_1$ No. of female (age at death in days). | Gen. F2. No. of female. | Gen. F2. Tumour age (days). | Gen. F2. Age of mice dying without tumours (days). | Gen. F3. No. of female. | Gen. F3. Tumour age (days). | Gen. F3. Age of mice dying without tumours (days). | Gen. F4. No. of female. | Gen. F4. Tumour age (days). | Gen. F4. Age of mice dying without tumours (days). |
|---|---|---|---|---|---|---|---|---|---|
| 26 (554) | 41 (VI) | — | 501 | 25 (I) | 409 | — | 54 (I) | — | 744 |
| | | | | | | | 55 (I) | — | 767 |
| | | | | 27 (I) | — | 420 | 14 (I) | — | 744 |
| | | | | | | | 15 (I) | — | 744 |
| | 42 (VI) | — | 501 | 51 (III) | — | 636 | 72 (II) | — | 618 |
| | | | | 52 (VI) | — | 101 | 30 (I) | 278 | — |
| | | | | | | | 31 (I) | 286 | — |
| | 43 (VI) | — | 500 | 65 (III) | — | 524 | 90 (I) | 592 | — |
| | | | | | | | 91 (I) | 512 | — |
| | | | | 66 (III) | — | 637 | 12 (II) | 571 | — |
| | | | | | | | 13 (II) | 311 | — |
| 31 (582) | 20 (IX) | — | 559 | 71 (IX) | — | 504 | 5 (I) | — | 556 |
| | | | | | | | 6 (I) | — | 591 |
| | | | | | | | 7 (I) | 446 | — |
| 33 (665) | 48 (II) | — | 535 | 79 (I) | — | 556 | 25 (I) | — | 464 |
| | | | | | | | 26 (II) | — | 480 |
| | | | | 80 (I) | — | 486 | 42 (I) | — | 634 |
| | | | | | | | 43 (II) | 526 | — |
| 21 (748) | 11 (V) | — | 832 | 33 (IV) | — | 602 | 64 (I) | — | 689 |
| | | | | 34 (IV) | — | 674 | 66 (I) | — | 568 |
| | | | | | | | 67 (I) | — | 227 |
| | 13 (V) | — | 664 | 29 (VI) | — | 499 | 60 (II) | — | 530 |
| | | | | 30 (VI) | — | 613 | 57 (I) | — | 571 |
| | | | | | | | 58 (II) | — | 650 |
| | | | | 31*(VI) | — | 609 | 62 (II) | 428 | — |

| | | | | | | | | | |
|---|---|---|---|---|---|---|---|---|---|
| 72 (315) . | 18 (VI) | — | 577 . | 38 (VII) | — | 250 . | 20 (I) | — | 392 |
| | | | | | | | 21 (II) | 311 | — |
| | | | | 39 (VII) | — | 726 . | 23 (VII) | — | 453 |
| | | | | | | | 24 (I) | — | 429 |
| | | | | 41 (VII) | — | 618 . | 26 (I) | — | 409 |
| | | | | | | | 27 (I) | — | 697 |
| | 19 (VI) | — | 532 . | 36 (VI) | — | 565 . | 17 (I) | 702 | — |
| | | | | | | | 18 (I) | — | 316 |
| 40 (397) . | 38 (V) | — | 724 . | 62 (II) | — | 659 . | 77 (I) | — | 597 |
| | | | | | | | 78 (I) | — | 515 |
| | | | | 63 (II) | — | 433 . | 1 (I) | 515 | — |
| | | | | | | | 2 (I) | — | 612 |
| | | | | | | | 3 (I) | — | 613 |
| | 39 (V) | 502 | — . | 59 (II) | — | 336 . | 87 (I) | — | 364 |
| | | | | | | | 88 (I) | — | 484 |
| | | | | 60 (II) | 484 | — . | 84 (I) | 276 | — |
| | | | | | | | 85 (I) | 432 | — |
| 39 (672) . | 15 (VI) | — | 420 . | 1 (VI) | — | 529 . | 1 (I) | — | 421 |
| | | | | | | | 2 (I) | — | 626 |
| | 16 (VI) | — | 545 . | 73 (XIII) | — | 244 . | 39 (II) | 391 | — |
| | | | | | | | 40 (II) | 644 | — |
| | | | | 74 (IX) | 452 | — . | 15 (II) | — | 446 |
| | | | | | | | 16 (II) | — | 511 |
| 36 (606) . | 8 (IV) | — | 582 . | 43 (IX) | — | 554 . | 29 (I) | — | 341 |
| | | | | 44 (IX) | — | 448 . | 33 (I) | — | 746 |
| | | | | | | | 34 (I) | — | 750 |
| | | | | 45 (IX) | — | 662 . | 31 (I) | — | 689 |
| 27 (478) . | 24 (V) | — | 491 . | 57 (V) | — | 365 . | 74 (I) | — | 517 |
| | | | | | | | 75 (I) | — | 475 |

Roman numbers in parentheses = number of litter in which the female was born.

* Tumour appeared and regressed.

TABLE V.—*Tumour Incidence in Forcibly-Bred Progeny of Tumorous* ($C57 \times RIII$)$F_1$ *Hybrid Females.*

| Generation. | Number of mice alive at the earliest tumour appearance. | Earliest tumour appearance (days). | Mice developing tumours / Mice dying without tumours. Age in months. 4. | 6. | 7. | 8. | 9. | 10. | 11. | 12. | 13. | 14. | 15. | 16. | 17. | 18. | 19. | 21. | Number of tumours. | Tumour incidence (%). | Average tumour age (days). | Number mice dead without tumours. | Average age of mice dying without tumours (days). | Number of mice alive. | Average age of mice alive (days). |
|---|---|---|---|---|---|---|---|---|---|---|---|---|---|---|---|---|---|---|---|---|---|---|---|---|
| 2 | 3 | 284 | – | – | – | – | 1/0 | 1/0 | – | – | – | – | – | – | – | – | 0/1 | – | 2 | 67 | 285 | 1 | 560 | – | – |
| 3 | 6 | 246 | – | – | – | 1/0 | 2/0 | – | 1/0 | – | 0/1 | – | – | – | – | – | 0/1 | – | 4 | 67 | 281 | 2 | 485 | – | – |
| 4 | 13 | 226 | – | – | – | 3/0 | 1/0 | 2/1 | 1/0 | – | 1/0 | – | 1/0 | 1/0 | 1/0 | – | – | 0/1 | 11 | 85 | 338 | 2 | 456 | – | – |
| 5 | 26 | 203 | – | – | 1/1 | 2/1 | 3/0 | 1/0 | 4/0 | 4/0 | – | 1/0 | 3/0 | 1/1 | – | 1/1 | 0/1 | – | 21 | 81 | 349 | 5 | 412 | – | – |
| 6 | 38 | 223 | – | – | – | 5/2 | 3/0 | 5/0 | 4/1 | 3/1 | – | 4/0 | 1/1 | 1/1 | 0/1 | – | – | – | 26 | 68 | 329 | 7 | 381 | 5 | 499 |
| 7 | 55 | 125 | 1/0 | 2/1 | 4/0 | 5/2 | 13/1 | 5/0 | 3/0 | 5/0 | 4/1 | 1/0 | 2/0 | 1/2 | – | – | – | – | 46 | 84 | 292 | 7 | 309 | 2 | 351 |

was high, with only slight variations from 67 to 88 per cent. Similarly the average tumour age showed comparatively small changes and was quite low. The majority of tumours in each generation developed during the first 12 months. The age of appearance of the earliest tumour showed gradual decrease from 284 days in the second to 125 days in the seventh generation. In Table VI is presented the age

FIG. 1.—Chart of forcibly-bred progeny of tumorous (C57 × RIII) $F_1$ No. 69 female.

Roman numbers in parentheses = consecutive number of litter ; T = tumour ; N = died ; L = alive. Number preceding letters T, N or L = number of female ; number following letters T, N or L = tumour age or life span in days.

of the earliest tumour appearance, the appearance of mammary tumours according to months, the tumour incidence, and the average tumour age in six generations of combined forcibly-bred progeny of the thirteen non-tumorous (C57 × RIII)$F_1$ hybrid females. The tumour incidence increased from 19 per cent in the second to nearly 60 per cent in the fifth and later generations. The average tumour age showed a gradual decrease from 16 to 11 months in the seventh generation, and similarly the age of appearance of the earliest mammary tumour

TABLE VI.—*Tumour Incidence in Forcibly-Bred Progeny of Non-Tumorous* ($C57 \times RIII$)$F_1$ *Hybrid Females.**

| Generation. | Number of mice alive at earliest tumour appearance. | Earliest tumour appearance (days). | Mice developing tumours. / Mice dying without tumours. Age in months. | | | | | | | | | | | | | | | | | | | | | | | | Number of tumours. | Tumour incidence (%). | Average tumour age (days). | Number of mice dead without tumours. | Average age of mice dying without tumours (days). | Number of mice alive. | Average age of mice alive (days). |
|---|---|---|---|---|---|---|---|---|---|---|---|---|---|---|---|---|---|---|---|---|---|---|---|---|---|---|---|---|---|---|---|---|---|
| | | | 5. | 6. | 7. | 8. | 9. | 10. | 11. | 12. | 13. | 14. | 15. | 16. | 17. | 18. | 19. | 20. | 21. | 22. | 23. | 24. | 25. | 26. | 28. | 30. | | | | | | | |
| 2 | 21 | 308 | – | – | – | – | – | 1/0 | – | – | – | 0/1 | – | 0/1 | 2/4 | 1/3 | 0/3 | – | – | 0/1 | – | 0/2 | – | – | 0/1 | 0/1 | 4 | 19 | 467 | 17 | 592 | — | — |
| 3 | 39 | 265 | – | – | – | – | 1/0 | 1/0 | 2/1 | 1/1 | 2/0 | 1/2 | 1/2 | 1/3 | 0/3 | 0/3 | 0/2 | 0/3 | 0/3 | 0/3 | 0/1 | 0/2 | – | – | – | – | 10 | 26 | 390 | 29 | 553 | — | — |
| 4 | 83 | 187 | – | 1/0 | 1/1 | – | 3/0 | 8/3 | 1/3 | 2/1 | 2/0 | 3/3 | 3/4 | 1/2 | 2/6 | 1/2 | 2/7 | 1/5 | 0/4 | 0/1 | 1/3 | – | 0/5 | 0/1 | – | – | 32 | 39 | 393 | 51 | 512 | — | — |
| 5 | 53 | 165 | – | 1/0 | 1/0 | 1/0 | 1/2 | 5/0 | 8/0 | 2/1 | 3/2 | 4/3 | 1/1 | 1/1 | 2/2 | – | 2/3 | 0/1 | 0/2 | 0/1 | 0/2 | – | – | – | – | – | 32 | 60 | 372 | 21 | 502 | — | — |
| 6 | 94 | 184 | – | 3/0 | 0/1 | 6/1 | 10/1 | 6/1 | 5/3 | 5/5 | 3/1 | 2/3 | 1/4 | 1/1 | 2/7 | 1/2 | 0/2 | 0/2 | 1/1 | 0/1 | 0/1 | – | 0/1 | – | – | – | 46 | 49 | 325 | 38 | 459 | 10 | 594 |
| 7 | 166 | 143 | 1/2 | 5/1 | 8/3 | 10/3 | 20/1 | 10/0 | 11/5 | 6/5 | 7/3 | 5/5 | 2/3 | 1/4 | 4/5 | 2/4 | 2/1 | – | 1/0 | – | – | – | – | – | – | – | 95 | 55 | 318 | 45 | 389 | 26 | 529 |

* Progeny of four females continued for six generations, and progeny of nine females continued for three generations.

decreased gradually from 10 months in the second to 5 months in the last generation. The distribution of breast tumours in the normally-bred progeny of (C57 × RIII)$F_1$ No. 22 female which had also been bred in a normal way is shown in Fig. 7. Again the number of breast tumours which appeared in each successive

FIG. 2.—Chart of forcibly-bred progeny of tumorous (C57 × RIII)$F_1$ No. 34 female.

Roman numbers in parentheses = consecutive number of litter ; T = tumour ; N = died ; L = alive. Number preceding letters T, N or L = number of female; number following letters T, N or L = tumour age or life span in days.

generation of the progeny of this non-cancerous hybrid female may be seen in this figure as well as the consecutive number of litter to which the mice belonged which developed tumours. It would have been of interest to obtain the first litter of this female and its progeny and compare any mammary tumour incidence in this progeny with that of the progeny obtained from the sixth litter, but this could not be done owing to limitation of space. Table VII shows the age of earliest tumour appearance, the tumour incidence, and the average tumour age in the normally-bred progeny of this non-tumorous (C57 × RIII)$F_1$ hybrid female. As can be seen, the age of the earliest tumour appearance decreased gradually from 11 to 4 months in the sixth generation of inbreeding ; mice of each generation,

FIG. 3.—Chart of forcibly-bred progeny of non-tumorous (C57 × RIII)$F_1$ No. 21 female.

Roman numbers in parentheses = consecutive number of litter ; T = tumour ; N = died ; L = alive. Number preceding letters T, N or L = number of female ; number following letters T, N or L = tumour age or life span in days.

except the second, developed a high incidence of breast cancer, and the average tumour age decreased from 16 to 11 months in the last generation.

A comparison of the mammary tumour incidence in the progeny of (C57 × RIII)$F_1$ hybrid females which died free of cancer and in the progeny of (C57 × RIII)$F_1$ hybrids which developed breast cancer is shown in Table VIII. The breast tumour incidence in the forcibly-bred progeny of the non-cancerous (C57 × RIII)$F_1$ hybrid females was lower and the average tumour age considerably higher than those in the forcibly-bred progeny of cancerous (C57 × RIII)$F_1$ hybrid females, but this difference in tumour incidence and average tumour age gradually became smaller in successive generations of the progeny. The difference in the age of the earliest tumour appearance in the descendants of these two types of

TABLE VII.—*Tumour Incidence in Normally-Bred Progeny of a Non-Tumorous* ($C57 \times RIII$)$F_1$ *Hybrid Female.*

| Generation. | Number of mice alive at earliest tumour appearance. | Earliest tumour appearance (days). | Number of mice developing tumours / Number of mice dying without tumours. Age in months. 4. | 9. | 10. | 11. | 12. | 13. | 14. | 15. | 16. | 17. | 19. | 20. | 21. | Number of tumours. | Tumour incidence (%). | Average tumour age (days). | Number of mice dead without tumour. | Average age of mice dying without tumour (days). | Number of mice alive. |
|---|---|---|---|---|---|---|---|---|---|---|---|---|---|---|---|---|---|---|---|---|---|
| 2 | 1 | – | – | – | – | – | – | – | – | 0/1 | – | – | – | – | – | – | – | – | 1 | 447 | – |
| 3 | 4 | 321 | – | – | – | 1/0 | – | – | – | 1/0 | – | 1/0 | – | – | 1/0 | 4 | 100 | 481 | – | – | – |
| 4 | 5 | 424 | – | – | – | – | – | – | 1/0 | 1/1 | – | 1/1 | – | – | – | 3 | 60 | 454 | 2 | 477 | – |
| 5 | 13 | 381 | – | – | – | – | – | 1/1 | 3/0 | 2/1 | – | – | 0/1 | 0/1 | 0/1 | 6 | 46 | 420 | 5 | 525 | 2 |
| 6 | 28 | 121 | 1/0 | 2/1 | 1/0 | 3/0 | 5/1 | – | 1/0 | 0/1 | 0/1 | 0/1 | – | – | – | 13 | 46 | 325 | 5 | 415 | 10 |

FIG. 4.—Chart of forcibly-bred progeny of non-tumorous (C57 × RIII)$F_1$ No. 68 female.

Roman numbers in parentheses = consecutive number of litter ; T = tumour ; N = died ; L = alive. Number preceding letters T, N or L = number of female ; number following letters T, N or L = tumour age or life span in days.

TABLE VIII.—*Comparison of Tumour Incidence in Progeny of Tumorous and Non-Tumorous (C57 × RIII)F$_1$ Hybrid Females.*

| Generation. | Tumorous females. Forcibly bred progeny.* | | | Non-tumorous females. Forcibly bred progeny.† | | | Non-tumorous females. Normally bred progeny.‡ | | |
|---|---|---|---|---|---|---|---|---|---|
| | Earliest tumour appearance (days). | Tumour incidence (%). | Average tumour age (days). | Earliest tumour appearance (days). | Tumour incidence (%). | Average tumour age (days). | Earliest tumour appearance (days). | Tumour incidence (%). | Average tumour age (days). |
| F2 | 284 | 67 | 285 | 308 | 19 | 467 | — | 0 | — |
| F3 | 246 | 67 | 281 | 265 | 26 | 390 | 321 | 100 | 481 |
| F4 | 226 | 85 | 338 | 187 | 39 | 393 | 424 | 60 | 454 |
| F5 | 203 | 81 | 349 | 165 | 60 | 372 | 381 | 46 | 420 |
| F6 | 223 | 68 | 329 | 184 | 49 | 325 | 121 | 46 | 325 |
| F7 | 125 | 84 | 292 | 143 | 55 | 318 | | | |

* Progeny of 2 females.
† Progeny of 13 females (of 9 females continued for 3 generations only, and of the remaining females for six generations).
‡ Progeny of 1 female.

hybrid females was less apparent, and in the later generations of the progeny of non-tumorous hybrid females it was even lower than that in the progeny of tumorous (C57 × RIII)F$_1$ hybrids. It may be of interest to point out that Foulds (1949) recorded a very low breast tumour incidence (1 to 3 per cent) at a late age (16 months) in forcibly-bred progeny of a non-tumorous (C57 × RIII)F$_1$ hybrid compared with a high (70 per cent) at 11 months in the progeny of three tumorous (C57 × RIII)F$_1$ hybrid females. The age of the earliest tumour appearance and the average tumour age in the progeny bred in a normal way of one cancer-free (C57 × RIII)F$_1$ hybrid female was distinctly higher than those in the forcibly-bred progeny of both tumorous and tumour-free (C57 × RIII)F$_1$ hybrids. The tumour incidence in the normally-bred progeny was, with one exception, lower than that in the forcibly-bred progeny of tumorous hybrid females and, again with one exception, approximated to that in the forcibly-bred progeny of non-tumorous hybrid females. It should be pointed out that the results of the comparison of the behaviour of normally-bred progeny of one non-tumorous female with that of the forcibly-bred progeny of thirteen non-tumorous hybrid females may not be the same as those which would have been obtained should this comparison have been extended to similarly maintained progeny of a number of other cancer-free hybrids which had been bred in a normal way. This point may be seen from the observations on the forcibly-bred progeny of four non-tumorous (C57 × RIII)F$_1$ hybrids compared with those on similarly treated progeny of two tumorous (C57 × RIII)F$_1$ hybrid females. The difference in mammary tumour incidence and average tumour age between these two groups of hybrid females became pronounced only after including the observations on the progeny of nine additional non-tumorous (C57 × RIII)F$_1$ hybrid females. It is therefore possible that the results of the comparison of normally with forcibly-bred progeny of non-cancerous hybrids would have been different as in the case of the comparison of forcibly-bred progeny of non-tumorous with that of tumorous hybrids. It is not known whether the results of Foulds' (1949) comparison of forcibly-bred progeny from tumorous and non-tumorous hybrid females would

FIG. 5.—Chart of forcibly-bred progeny of non-tumorous (C57 × RIII)$F_1$ No. 38 female.

Roman numbers in parentheses = consecutive number of litter ; T = tumour ; N = died ; L = alive. Number preceding letters T, N or L = number of female ; number following letters T, N or L = tumour age or life span in days.

**Fig. 6.**—Chart of forcibly-bred progeny of non-tumorous (C57 × RIII) $F_1$ No. 20 female.

Roman numbers in parentheses = consecutive number of litter ; T = tumour ; N = died ; L = alive. Number preceding letters T, N or L = number of female ; number following letters T, N or L = tumour age or life span in days.

not have been different had the progeny of a greater number of non-tumorous hybrid females been included in his study.

From the study of the distribution of cancerous females in the forcibly-bred progeny of tumorous and tumour-free (C57 × RIII)$F_1$ hybrid females and in the normally-bred progeny of a cancer-free hybrid, shown in Fig. 1 to 7 and in Table

FIG. 7.—Chart of normally-bred progeny of non-tumorous (C57 × RIII)$F_1$ No. 22 female.

Roman numbers in parentheses = consecutive number of litter ; T = tumour ; N = died ; L = alive. Number preceding letters T, N or L = number of female ; number following letters T, N or L = tumour age or life span in days.

IV, conclusions may be drawn about the parent-offspring correlation and the presence of the mammary tumour agent in these lines, especially in females with tumours. Although, perhaps at first sight, the mammary tumours may appear scattered throughout the pedigree-charts of some lines, yet even then in certain lines there appears to take place a segregation of high-cancer lines. Detailed analysis revealed a more positive parent-offspring correlation. In the progeny of the two (C57 × RIII)$F_1$ hybrid females with tumours, there were among the descendants of tumour-bearing mothers 82 daughters with tumours and 22 without mammary tumours, and among the progeny of mothers without breast cancer there were 23 daughters with tumours and 4 without breast tumours. In the forcibly-bred progeny of the thirteen cancer-free (C57 × RIII)$F_1$ hybrid females the tumorous mothers had 134 daughters which developed breast cancer and 67 which died cancer-free, and the non-tumorous mothers had 81 cancerous and 165 non-cancerous daughters. In the normally-bred progeny of one cancer-free (C57 × RIII)$F_1$ hybrid the segregation was not so evident : mothers with breast cancer had 16 tumorous and 12 non-tumorous daughters, and cancer-free mothers gave birth to 12 cancerous and 12 non-cancerous daughters. Thus in the majority of lines the chance that a female would develop mammary cancer was greater if her mother had a tumour than if her mother died without a breast tumour. In the progeny of the two (C57 × RIII)$F_1$ tumorous females even the progeny of tumour-free mothers showed a greater tendency towards tumour development than the progeny of tumour-free mothers derived from the thirteen (C57 × RIII)$F_1$ non-tumorous hybrid females. Therefore, there was a strong indication of the presence of the agent in these hybrids and of its transmission to their progeny which developed mammary cancer either after inbreeding alone or following inbreeding combined with intensive hormonal stimulation.

A search for the possible influence of age on the development of mammary tumours in the hybrid progeny (Tables I, II, V, VI and VII) showed that in all lines the age of tumour-bearing females was distinctly lower than that of tumour-free mice. Thus in the progeny of the two (C57 × RIII)$F_1$ females with tumours, the tumour-bearing mice lived to an average age of 11 months before the development of breast cancer and the tumour-free mice to an average age of 14 months. In the descendants of the thirteen non-tumorous hybrid females, the average age of mice with cancer was 13 months and of mice without breast tumours 17 months. The average tumour age of mice in the progeny of a non-tumorous (C57 × RIII)$F_1$ female, which had been bred in a normal way, was 14 months, and the average life-span of mice without breast cancer was 16 months. Thus the age, as such, had no influence on the development of breast cancer in these hybrid females.

As can be seen in Tables IX and X, the study of a possible influence of the number of litters born to each female on the development of breast cancer in the hybrid mice revealed that among the descendants of the two tumorous (C57 × RIII)$F_1$ hybrid females, the tumour-bearing mice had an average of 6·2 (from 1 to 13) litters and the tumour-free mice an average of 6·7 (2 to 13) litters. Among the forcibly-bred progeny of the four tumour-free (C57 × RIII)$F_1$ hybrids, tumorous mice had an average of 7·3 (1 to 13) and the mice without tumours an average of 8·3 (1 to 16) litters. A greater number of litters was therefore required before mice developed breast cancer in the progeny of tumour-free (C57 × RIII)$F_1$ hybrids than that required in the descendants of tumorous (C57 × RIII)$F_1$ before they showed breast cancer. This difference is even more pronounced in the progeny of nine tumour-free (C57 × RIII)$F_1$ hybrid females, in which mice with breast tumours had an average of 9·2 (5 to 13) litters and tumour-free mice an average of 9·5 (2 to 16) litters. In the combined descendants of the thirteen tumour-free (C57 × RIII)$F_1$ hybrids, the average number of litters for tumorous mice was 7·4 (1 to 13) and for tumour-free mice 8·7 (1 to 16) litters. Cancerous

TABLE IX.—*Number of Litters Born in the Progeny of Tumorous* ($C57 \times RIII)F_1$ *Hybrid Females Before either Tumour Appearance or Death.*

| Generation. | No. of litter. 0. | 1. | 2. | 3. | 4. | 5. | 6. | 7. | 8. | 9. | 10. | 11. | 12. | 13. | Number of mice alive at earliest tumour appearance. | Number of mice alive. |
|---|---|---|---|---|---|---|---|---|---|---|---|---|---|---|---|---|
| 1 | – | – | – | – | – | – | 2/0 | – | – | – | – | – | – | – | 2 | – |
| 2 | – | – | – | – | – | – | – | 1/0 | – | 0/1 | 1/0 | – | – | – | 3 | – |
| 3 | – | – | – | – | 1/0 | 1/0 | 1/0 | 0/1 | 0/1 | 1/0 | – | – | – | – | 6 | – |
| 4 | – | – | – | 1/0 | – | 1/1 | 3/0 | 2/1 | 1/0 | 1/0 | 1/0 | – | – | 1/0 | 13 | – |
| 5 | 1/1 | – | 1/0 | 2/2 | 2/0 | 4/0 | 2/0 | 3/0 | 1/1 | 4/0 | – | – | 1/0 | 0/1 | 26 | – |
| 6 | – | 2/0 | 1/0 | 0/1 | 2/0 | 5/1 | 3/1 | 4/1 | 4/1 | 2/2 | 2/0 | – | 1/0 | – | 38 | 5 |
| 7 | – | – | 7/1 | 4/1 | 4/1 | 5/0 | 6/1 | 6/0 | 7/1 | 4/0 | 1/2 | 1/0 | 1/0 | – | 55 | 2 |

$\frac{\text{Numerators}}{\text{Denominators}} = \frac{\text{Number of mice developing tumours.}}{\text{Number of mice dying without tumours.}}$

mice in the progeny of the one ($C57 \times RIII)F_1$ non-tumorous hybrid which had been bred in a normal way had an average of 6·2 (2 to 10) and non-cancerous mice an average of 6·1 (2 to 12) litters. Thus the mice which developed breast cancer in each of the lines did not have more litters than the mice which died without developing a tumour, but the progeny of the thirteen non-tumorous hybrid females had a greater number of litters before the appearance of a mammary tumour than the progeny of the two tumorous hybrids.

A connection between inbreeding and probably hormonal stimulation and the appearance of breast cancer in the progeny of these hybrids was found after an assessment of the number of litters born to females before they developed cancer. As can be seen in Tables IX and X, in mice of each generation of progeny of both cancerous and non-cancerous ($C57 \times RIII)F_1$ hybrids, the number of females which had only five litters before developing a tumour increased in each successive generation, although in all generations the majority of females had more than five litters before the appearance of breast cancer. As shown in Table XI, the number of tumour-bearing females born in the first litter also increased in each successive generation, in spite of the increasing number of tumorous females born in later litters. Further, an increase in the amount of the tumour agent as a result of securing later litters for obtaining progeny for each successive generation and its

TABLE X.—*Number of Litters Born in the Forcibly-Bred Progeny of Non-Tumorous* ($C57 \times RIII$)$F_1$ *Hybrid Females Before either Tumour Appearance or Death.**

| Generation. | No. of litter. 0. | 1. | 2. | 3. | 4. | 5. | 6. | 7. | 8. | 9. | 10. | 11. | 12. | 13. | 14. | 15. | 16. | Number of mice alive at earliest tumour appearance. | Number of mice alive. |
|---|---|---|---|---|---|---|---|---|---|---|---|---|---|---|---|---|---|---|---|
| 1 | – | – | 0/1 | – | – | 0/1 | 0/8 | 0/1 | – | 0/1 | 0/1 | – | – | – | – | – | – | 0 | – |
| 2 | – | – | – | – | – | – | – | 0/1 | 0/2 | 2/1 | 0/8 | 2/1 | 0/2 | 0/2 | – | – | – | 21 | – |
| 3 | – | – | 0/1 | 0/1 | 1/0 | – | 1/1 | 2/2 | 2/2 | 0/4 | 2/3 | 0/7 | 0/3 | 3/1 | – | – | 0/3 | 39 | – |
| 4 | – | – | – | 1/1 | 0/2 | 2/3 | 5/6 | 5/5 | 5/8 | 7/12 | 4/2 | 3/2 | 0/3 | 0/2 | 0/4 | 0/1 | – | 83 | – |
| 5 | – | – | – | – | 1/1 | 2/2 | 6/2 | 5/0 | 7/5 | 3/1 | 6/6 | 1/2 | – | 1/2 | – | – | – | 53 | – |
| 6 | 0/1 | 1/1 | 1/0 | 4/0 | 2/2 | 6/4 | 5/2 | 8/8 | 8/2 | 2/4 | 5/3 | 3/3 | 1/1 | 0/3 | 0/2 | – | 0/2 | 94 | 10 |
| 7 | 4/4 | 1/3 | 0/1 | 5/1 | 10/6 | 9/2 | 14/5 | 8/4 | 17/5 | 10/5 | 5/1 | 8/3 | 2/2 | 1/3 | – | – | – | 166 | 26 |

$\frac{\text{Numerators}}{\text{Denominators}} = \frac{\text{Number of mice developing tumours.}}{\text{Number of mice dying without tumours.}}$

* Progeny of four females continued for six generations, and progeny of nine females continued for three generations.

TABLE XI.—*Number of Tumorous Mice in the Descendants of* $(C57 \times RIII)F_1$ *Hybrid Females According to Number of Litter in which they were Born.*

Tumorous:

| Generation. | No. of litter: 1. | 2. | 3. | 4. | 5. | 6. | 7. | 9. | 10. | Total. |
|---|---|---|---|---|---|---|---|---|---|---|
| 2 | — | — | 2/0 | — | — | 0/1 | — | — | — | 2/1 |
| 3 | — | — | — | — | — | — | — | 1/1 | 3/1 | 4/2 |
| 4 | 2/2 | 3/0 | 1/0 | — | 5/0 | — | — | — | — | 11/2 |
| 5 | 15/3 | 4/2 | 2/0 | — | — | — | — | — | — | 21/5 |
| 6 | 19/6 | 4/0 | 3/0 | 0/1 | — | — | — | — | — | 26/7 |
| 7 | 26/4 | 11/0 | 2/0 | 2/1 | 1/1 | — | 2/0 | 2/1 | — | 46/7 |

Non-tumorous.*:

| Generation. | No. of litter: 1. | 2. | 3. | 4. | 5. | 6. | 7. | 8. | 9. | 10. | 11. | Total. |
|---|---|---|---|---|---|---|---|---|---|---|---|---|
| 2 | — | 0/1 | — | 1/2 | 1/4 | 1/8 | 1/1 | — | 0/1 | — | — | 4/17 |
| 3 | 1/3 | 1/3 | 0/3 | 0/2 | 0/1 | 0/7 | 1/1 | 0/2 | 3/5 | 4/0 | 0/2 | 10/29 |
| 4 | 16/37 | 11/10 | 0/1 | — | 1/1 | 1/1 | 1/1 | 2/0 | — | — | — | 32/51 |
| 5 | 17/9 | 10/7 | 3/4 | 1/1 | — | — | 1/0 | — | — | — | — | 32/21 |
| 6 | 24/21 | 15/12 | 3/1 | 0/2 | 2/1 | 0/1 | — | — | 2/0 | — | — | 46/38 |
| 7 | 56/30 | 14/14 | 11/0 | 4/0 | 5/1 | 2/0 | 1/0 | 2/0 | — | — | — | 95/45 |

$$\frac{\text{Numerators}}{\text{Denominators}} = \frac{\text{Number of mice developing tumours.}}{\text{Number of mice dying without tumours.}}$$

* Combined progeny of fourteen non-tumorous females.

activation by inbreeding and intensive hormonal stimulation (forced breeding) must also be taken into consideration as a possible cause of the increasing number of tumorous females born in the first litter in each successive generation.

Foulds (1947, 1949) reported an incidence of 15 per cent of mammary tumours at an average age of 10 months in forcibly-bred hybrids of similar derivation. This compares with an incidence of 14 per cent at 13·3 months in (C57 × RIII)$F_1$ hybrids of the present study. Foulds (1949) noted that the distribution of mammary tumours in the hybrid progeny of C57 Black strain mothers suggested a strong familial factor, the tumours appearing mostly in litters of certain C57 females. This limitation of tumorous hybrid females to certain C57 strain mothers could not be detected in the present study, because although only four hybrid females developed breast cancer, the majority of (C57 × RIII)$F_1$ hybrids transmitted the tendency to tumour development. Therefore, the progeny not only of tumorous but also the progeny of the majority of non-tumorous (C57 × RIII)$F_1$ hybrids, in spite of individual variations, developed a high incidence of breast cancer. It appears also that the majority of non-tumorous hybrid females transmitted the mammary tumour agent to their progeny, thus showing no limitation to certain C57 Black strain mothers. It is probable that the majority of mammary tumours are similar to those which developed in the minority of hybrid females or their back-cross progeny in the experiments of Andervont and Dunn (1948*a*, 1948*b*, 1949, 1950*b*). While the tendency to mammary tumour development in the hybrid progeny of susceptible low-cancer-strain females was not limited to certain of these females in the studies of Andervont and Dunn (1950*b*), the tendency to the transmission of the mammary tumour agent was limited to the hybrid progeny of a few females. Thus, the tendency to breast tumour development and/or to transmission of the agent may vary in the hybrid progeny according to the origin of their low-cancer-strain mothers and probably also male parents, and they may also vary in the hybrid progeny of low-cancer-strain mothers from different sublines of the same strain. Foulds (1949) reported data which suggested the presence and transmission of the agent in hybrids of similar derivation to that of hybrids in the present study, but pointed out that his (C57 × RIII)$F_1$ hybrid females were not bred for determination of mammary tumour incidence. Observations on hybrids of the present experiments strongly indicate the presence and transmission of the agent by at least the majority of hybrid females.

In addition, the incidence of mammary tumours was examined in litters of several hybrid females before and after they had developed tumours. This was done in an attempt at ascertaining any possible increase in the tumour incidence and in the amount of the agent after tumour development (Table XII). In a case of two hybrid females, there was no difference in the number of tumours in mice of litters and several generations of their progeny before and after the development of breast cancer in their hybrid mothers, both types of litters showing a high incidence of mammary tumours. In a case of two other females there were no tumours in litters before they had developed mammary cancer but tumours appeared in litters obtained after these females had shown breast tumours, and this may indicate an increase in the amount of the agent present or its appearance only after the development of a mammary tumour in these hybrid females. The number of tumours increased in the litter of one female obtained after she had developed mammary cancer, and in the case of another hybrid female a tumour developed in a littler obtained before but no tumours in litters secured

TABLE XII.—*Tumour Incidence in Litters Born Before and After the Appearance of a Tumour.*

| Generation. | Before tumour development. | | | | After tumour development. | | | |
|---|---|---|---|---|---|---|---|---|
| | Number of mice alive at earliest tumour appearance. | (Consecutive no. of litter) Number of tumours. | Average tumour age (days). | Average age of mice dying without tumours (days). | Number of mice alive at earliest tumour appearance. | (Consecutive no. of litter) Number of tumours. | Average tumour age (days). | Average age of mice dying without tumours (days). |
| | | | | *No.* 22. *F2 T* (308).* | | | | |
| | | (IX) | | | | (X) | | |
| F3 | 2 | 2 | 297 | — | 2 | 2 | 330 | — |
| F4 | 4 | 4 | 353 | — | 5 | 2 | 286 | 454 |
| F5 | 11 | 7 | 359 | 408 | 9 | 5 | 334 | 506 |
| F6 | 16 | 8 | 289 | 374 (2L) | 15 | 12 | 289 | 327 |
| F7 | 26 | 20 | 275 | 298 (3L) | 29 | 21 | 281 | 372 (2L) |
| | | | | *No.* 21. *F3 T* (265).* | | | | |
| | | (VI) | | | | (IX) | | |
| F4 | 1 | 1 | 287 | — | 2 | 2 | 377 | — |
| F5 | 66 | 4 | 366 | 470 | 3 | 3 | 346 | — |
| F6 | 7 | 3 | 262 | 364 | 7 | 3 | 257 | 395 (2L) |
| F7 | 11 | 9 | 284 | 262 | 13 | 9 | 275 | 372 (3L) |
| | | | | *No.* 37. *F5 T* (290).* | | | | |
| | | (II) | | | | (VI) | | |
| F6 | 2 | 1 | 477 | — (1L) | (3)† | — | — | 485 (2L) |
| F7 | (4)† | — | — | 399 | (6)† | — | — | — (6L) |
| | | | | *No.* 76. *F5 T* (299).* | | | | |
| | | (III) | | | | (IX) | | |
| F6 | (2)† | — | — | 321 | 2 | 1 | 363 | — (1L |
| F7 | 1 | 1 | 364 | — | 3 | 3 | 354 | — |
| | | | | *No.* 13. *F5 T* (165).* | | | | |
| | | (I) | | | | (IV) | | |
| F6 | (1)† | — | — | 452 | (2)† | — | — | 234 |
| F7 | (1)† | — | — | 327 | 2 | 1 | 417 | — (1L) |
| | | | | *No.* 10. *F5 T* (329).* | | | | |
| | | (I) | | | | (IX) | | |
| F6 | (2)† | — | — | 218 | 2 | 1 | 290 | 407 |
| F7 | — | — | — | — | — | — | — | — |

* Tumour age. † All mice included, as no tumours appeared.

after the development of breast cancer. Accordingly it could be deduced that some hybrid females possessed the agent before and after tumour development, which may be the case, as the litters which were compared were late litters; further, some females harboured the agent after tumour development and others showed an increase in its amount, and still others showed the presence of the agent before and a decrease or absence of it after mammary tumour development. Andervont and Dunn (1949) observed breast tumours in the progeny of a hybrid female before tumour development and no tumours in mice of the litter secured after the female had developed cancer. The behaviour of mice in the progeny obtained after the tumour appeared in their mother may greatly vary even in the case of hybrid females of the same origin, probably as a result of variations in the genetic make-up of individual females influencing the presence or transmission of the agent.

In conclusion it may be stated that both the genetic constitution and hormonal stimulation play an important part in the development of mammary cancer in the hybrid mice which were the subject of the present study, and that the mammary tumour agent also appears to take a part in the origin of these tumours.

*Biological Tests for the Presence of the Mammary Tumour Agent.*

As soon as the first mammary tumours appeared in the hybrid mice, biological tests for the presence of the agent were carried out. Altogether twenty-three mammary tumours were tested and the results are shown in Table XIII. Of the four mammary tumours which developed in (C57 × RIII)$F_1$ hybrid females, two showed the presence of the agent and two others failed to reveal the agent under the conditions of the test. The age at which the two negative mammary tumours developed was 10 and 17 months respectively. At first it appears surprising that the tumour which developed at the age of 10 months failed to show the agent, as the time of storage in desiccated state was equal to that of another breast tumour which appeared in the same generation and which, although injected in smaller quantity, induced tumours in the test mice but in distinctly smaller incidence compared with that induced by other dried agent-harbouring tissues in previous experiments (Dmochowski, 1945*a*, 1945*b*, 1946, 1948, 1949*c*). It must be stressed that the method of desiccation of these two as well as of all the other tumours was the same, and the age of the test mice used was in all cases approximately the same. The only varying factors were the amount of tissue injected and the time of storage. The longest period of storage in the ice-chest was that of a negative tumour in a (C57 × RIII)$F_1$ hybrid female and amounted to 8 months. This should not have made an appreciable difference in the test, as it was previously shown that agent-harbouring tissue induced breast cancer in test mice after two years of storage (Dmochowski, 1946). From the results of the tests of the four tumours in the first generation of hybrid females it may be concluded that two of them possessed the agent, and the two other negative tumours either did not contain the agent or harboured only small quantities or a weak agent which could not be shown under the conditions of the test. None of the tumours of the progeny of (C57 × RIII)$F_1$ No. 34 female whose tumour was found to harbour the agent was tested, but as can be seen in Fig. 2, from the study of parent-offspring correlation there was a definite tendency towards breast cancer development in daughters of tumorous as well as non-tumorous mothers. From this as well as from the high incidence of tumours it can be presumed that these tumours contained the agent. The results of the tests of breast tumours which developed in the progeny of the tumorous (C57 × RIII)$F_1$ No. 69 female (Fig. 1), whose tumour failed to reveal the agent, are of interest. Of the nine tumours tested in the progeny of this female, six were found to harbour the agent and three failed

TABLE XIII.—*Tumour Incidence in (C57 × A)$F_1$ Hybrid Females Injected with Extracts of Dried Tumour Tissue from Various (C57 × RIII) Hybrid Females.*

| No. and origin of tumour. | Age at which tumour appeared (days). | Time of storage of dried tumour tissue (days). | Number of injections. / Amount of fresh tissue injected (g.). | Number of mice injected. | Number of mice alive at earliest tumour appearance. | Mice developing tumours. / Mice dying without tumours. Age in months. 8. | 9. | 10. | 11. | 12. | 13. | 14. | 15. | 16. | 17. | 18. | 19. | 20. | 21. | 22. | 23. | Number of tumours. | Tumour incidence (per cent). | Average tumour age (months). | Average age of mice dying without tumours (months). |
|---|---|---|---|---|---|---|---|---|---|---|---|---|---|---|---|---|---|---|---|---|---|---|---|---|---|
| 22 F1 (A) (i) | 332 (ii) | 48 | 2/0·1 | 18 | 16 | 0/1 | 1/1 | — | 0/1 | 1/1 | 0/2 | 0/2 | 0/3 | 0/3 | — | — | — | — | — | — | — | 2 | 13 | 11 | 13 |
| 67 F1 (A) | 290 (ii) | 48 | 5/0·25 | 12 | 10 | — | — | — | — | 0/2 | 0/2 | 0/3 | 0/1 | — | — | 0/1 | 0/1 | — | — | — | — | — | — | — | 15 |
| 34 F1 (B) | 483 (iii) | 5 | 4/0·2 | 6 | 6 | — | — | — | — | 1/2 | 0/2 | — | — | 1/0 | — | — | — | — | — | — | — | 2 | 33 | 14 | 13 |
| 69 F1 (B) | 502 (iii) | 238 | 10/0·2 | 12 | 8 | — | — | — | — | — | — | — | — | — | — | 0/3 | 0/2 | — | — | 0/3 | — | — | — | — | 20 |
| 10 F2 (B) | 284 (T) (iv) | 2 | 4/0·2 | 12 | 10 | — | — | 0/1 | — | 0/1 | 0/2 | 0/1 | 0/1 | 0/4 | — | — | — | — | — | — | — | — | — | — | 14 |
| 9 F2 (B) | 285 (T) | 5 | 4/0·1 | 23 | 19 | — | — | — | 0/2 | 0/3 | 0/4 | 0/1 | 0/4 | — | — | 0/3 | — | — | 0/2 | — | — | — | — | — | 15 |
| 22 F2 (A) | 308 (N) (iv) | 159 | 8/0·2 | 18 | 13 | 0/4 | 0/2 | 1/0 | 0/1 | 1/1 | 0/1 | 0/1 | — | — | — | 0/1 | — | — | — | — | — | 2 | 15 | 11 | 11 |
| 37 F2 (A) | 529 (N) | 14 | 6/0·12 | 14 | 12 | — | — | — | — | — | — | 0/1 | 0/1 | — | — | 0/1 | — | — | — | 0/2 | — | — | — | — | 18 |
| 33 F2 (B) | 505 (N) | 58 | 7/0·175 | 18 | 14 | — | — | — | 0/1 | 0/5 | 0/1 | — | 0/1 | 0/1 | 1/0 | — | — | 0/1 | 1/1 | — | 0/1 | 2 | 14 | 19 | 15 |
| 39 F2 (D) | 502 (N) | 92 | 4/0·1 | 18 | 14 | — | — | 1/0 | — | 0/1 | 2/0 | 0/1 | 0/1 | 1/1 | 1/0 | 0/1 | 0/2 | 0/2 | — | — | — | 5 | 36 | 14 | 17 |
| 15 F3 (B) | 276 (T) | 14 | 5/0·1 | 12 | 10 | 1/1 | — | 0/1 | 1/3 | 0/1 | — | 0/1 | — | 1/0 | — | — | — | — | — | — | — | 3 | 30 | 12 | 11 |

| | | | | | | | | | | | | | | | | | | | | | | | | | |
|---|---|---|---|---|---|---|---|---|---|---|---|---|---|---|---|---|---|---|---|---|---|---|---|---|---|
| 20 F3 (B) | 242 (T) | 14 | 11/0·275 | 12 | 6 | – | – | 0/1 | – | 0/4 | – | 0/1 | – | – | – | – | – | – | – | – | – | — | — | — | 12 |
| 21 F3 (A) | 265 (N) | 45 | 7/0·28 | 15 | 10 | 1/2 | – | – | 0/2 | – | 0/1 | 1/0 | 0/2 | 1/0 | – | – | – | – | – | – | – | 3 | 30 | 13 | 12 |
| 47 F3 (B) | 360 (N) | 2 | 4/0·1 | 15 | 10 | 0/1 | – | – | 0/1 | – | 0/2 | 0/1 | 0/1 | – | 2/1 | 1/0 | – | – | – | – | – | 3 | 30 | 17 | 13 |
| 49 F3 (A) | 300 (N) | 155 | 3/0·12 | 18 | 15 | – | – | 1/0 | – | 1/1 | 3/1 | 1/0 | 2/0 | 1/0 | – | 1/2 | 0/1 | – | – | – | – | 10 | 67 | 14 | 16 |
| 69 F3 (A) | 403 (N) | 60 | 3/0·3 | 8 | 8 | – | 1/0 | 2/0 | – | 1/0 | 1/1 | 1/0 | 0/1 | – | – | – | – | – | – | – | – | 6 | 75 | 12 | 15 |
| 93 F4 (B) | 216 (N) | 2 | 5/0·125 | 18 | 12 | – | 0/1 | 1/0 | 1/0 | – | 1/1 | – | – | – | 1/0 | 0/1 | 0/2 | 0/2 | – | 0/1 | – | 4 | 33 | 13 | 18 |
| 48 F4 (B) | 249 (T) | 7 | 5/0·25 | 14 | 10 | – | 0/1 | – | 0/1 | – | 1/0 | 1/0 | 2/0 | 0/1 | 0/1 | – | 0/1 | 0/1 | – | – | – | 4 | 40 | 14 | 15 |
| 39 F4 (B) | 312 (N) | 82 | 5/0·125 | 21 | 16 | – | – | 2/0 | 1/1 | – | 2/0 | – | 1/1 | 2/0 | 1/0 | 1/0 | 0/1 | 0/2 | 0/1 | – | – | 10 | 63 | 14 | 18 |
| 50 F4 (B) | 498 (T) | 14 | 1/0·1 | 12 | 10 | – | – | – | 0/1 | 2/1 | 0/1 | 2/0 | 0/2 | – | – | 0/1 | – | – | – | – | – | 4 | 40 | 13 | 14 |
| 48 F5 (B) | 326 (T) | 20 | 3/0·12 | 10 | 10 | – | 1/0 | – | 1/1 | 1/0 | – | 1/0 | 1/2 | 0/1 | 0/1 | – | – | – | – | – | – | 5 | 50 | 13 | 15 |
| 89 F5 (A) | 378 (T) | 14 | 1/0·1 | 6 | 6 | – | – | 1/0 | – | 1/0 | 2/0 | 1/0 | – | 0/1 | – | – | – | – | – | – | – | 5 | 83 | 12 | 16 |
| 90 F5 (B) | 337 (T) | 14 | 1/0·1 | 11 | 7 | – | – | – | 0/1 | 0/1 | 1/0 | 0/1 | – | 1/1 | – | – | – | – | – | – | – | 3 | 42 | 15 | 14 |

(i) Letters A, B, or D in parentheses = type of mammary tumour found in histological examination.

(ii) Tumorous females with no progeny.

(iii) Tumorous females with progeny.

(iv) Letter T in parentheses = progeny of tumorous female; letter N in parentheses = progeny of non-tumorous female.

(v) Females from discontinued lines, not shown in fig. 1 to 7.

to show the agent. Mammary tumours of (C57 × RIII)$F_2$ No. 9 and No. 10, daughters of No. 69 (C57 × RIII)$F_1$ female, did not reveal the agent, although they developed comparatively early at about 10 months. The (C57 × RIII)$F_2$ No. 9 female had ten litters and her sister had seven litters. It may well be that the considerable hormonal stimulation was responsible for the appearance of breast cancer in these females, and more likely that the agent took part in the development of these tumours but in quantities too small to be detected by the test. (C57 × RIII)$F_3$ No. 15 and No. 20, were the two females in the progeny of (C57 × RIII)$F_2$ No. 9 hybrid whose tumours were tested. The test showed the presence of the agent in the tumour of No. 15 hybrid but not in that of No. 20 female, although the ages at which the two tumours developed were quite comparable. It may well be that had the test animals, in which the latter tumour was tested, lived longer, the agent would probably have been detected. In the fourth generation of the progeny of No. 69 (C57 × RIII)$F_1$ hybrid, the two breast tumours tested of No. 48 female, granddaughter of No. 9 (C57 × RIII)$F_2$ female, and of No. 50, daughter of No. 20 (C57 × RIII)$F_3$ hybrid, were found to harbour the agent. The first tumour developed at the age of 8 months after the female had six litters, and the second at approximately 17 months after the female had thirteen litters. It is of interest that both tumours showed the agent, although the second developed comparatively late in life. In the fifth generation of the same line three tumours were tested, those of No. 89 and 90 females, granddaughters of No. 15 (C57 × RIII)$F_3$ agent-harbouring female and of No. 48 female, daughter of No. 50 (C57 × RIII)$F_4$ agent-carrying hybrid. The agent was detected in all three tumours. From the biological tests of mammary cancer in the descendants of the original No. 69 (C57 × RIII)$F_1$ tumorous hybrid female in whose tumour the agent was not detected, it may be seen how necessary it is to adopt a cautious attitude towards the results of biological tests. It appears probable that the agent may have increased gradually in quantity in successive generations of the descendants of this hybrid female, so that it eventually could be detected by the method applied in the present biological tests. It is possible that the treatment, such as desiccation, may have in part been responsible for the negative results when tumours with small quantities of the agent were tested. Had the mammary tumour of No. 69 (C57 × RIII)$F_1$ hybrid only been tested and the progeny of this female not raised, the conclusion would have been that increased hormonal stimulation of a susceptible genetic substrate was responsible for the appearance of this breast tumour. It may well be stated that hormonal and genetic factors combined with the mammary tumour agent were responsible for the development of the majority of the tested breast tumours in this line, and probably also a weak or attenuated agent in the tumours negative in the biological test.

In the progeny of non-tumorous (C57 × RIII)$F_1$ No. 21 (Fig. 3), two tumours, one of (C57 × RIII)$F_3$ No. 69 and one of (C57 × RIII)$F_4$ No. 93 hybrids were tested. The agent was found in both tumours. Among the descendants of tumour-free (C57 × RIII)$F_1$ No. 38 hybrid (Fig. 5) two mammary tumours were tested, one which developed in (C57 × RIII)$F_2$ No. 33 female at the age of 17 months after she had eleven litters and one which arose in (C57 × RIII)$F_4$ No. 39 hybrid at the age of 10·5 months after she had given birth to six litters. The agent was detected in both mammary tumours. It is interesting to note the difference in tumour incidence induced in the test mice by these two tumours.

The first, which developed at the age of 17 months, gave only 14 per cent incidence compared with 63 per cent incidence induced by the tumour which developed at the age of 10·5 months. It appears that these two tumours differed in the amounts of the agent present. Among the descendants of the non-tumorous (C57 × RIII)$F_1$ No. 68 hybrid (Fig. 4) four tumours were examined. The tumour in No. 22 (C57 × RIII)$F_2$ female developed at the age of 10 months after she had nine litters, and three breast tumours of No. 21, 47, and 49 (C57 × RIII)$F_3$ hybrids, all of which developed after the eighth litter at the age of 9, 12, and 10 months respectively. All four tumours harboured the agent, yet, although they all arose comparatively early in life, the incidence of tumours induced in the test mice by the tumour of No. 22 $F_2$ female was only 15 per cent compared with that of 30 to 67 per cent by tumours of the third generation females, and that in spite of the time one of these tumours (No. 49, $F_3$) was stored, which was three times longer than that of No. 22 $F_2$ tumour.

An attempt to correlate the appearance of tumours and the presence of the agent with any particular coat colour of the hybrid mice showed that while all $F_1$ females were of " wild " colour, their progeny in general could be divided into " wild ", " white " and " black " coat colours. Tumours developed in hybrid mice of all three coat colours without particular connection with any coat colour. Foulds (1949) recorded a similar observation in his C57 × RIII hybrids. Neither the presence or absence of the agent, as shown in biological tests, was connected with any particular coat colour.

Foulds (1949) detected the agent in the tumour of a (C57 × RIII)$F_1$ hybrid which had been tested. Mühlbock (1952) failed to discover the agent in tumours of five (C57 × d) hybrids which developed at ages varying from 20 to 30 months. These hybrids came from early litters. Mammary tumours in hybrids from later litters of C57 females were not tested, but the presence of the agent in these tumours was assumed by Mühlbock (1952) on the basis of a high tumour incidence in the litters, although the average tumour age was 18 months. A search for the agent in mammary tumours of hybrid progeny from another (C) low-cancer-strain but susceptible females and high-cancer-strain (C3H) males led to the detection of the agent in tumours which developed at an early age up to 12 months, although on two occasions even such tumours were negative (Andervont, 1945*b* ; Andervont and Dunn, 1950*b*), while mammary tumours arising at a late age, in spite of their high incidence, failed to reveal the agent (Andervont and Dunn, 1949, 1950*b* ; Andervont, 1950*a*). Yet, on one occasion a C strain female was found to harbour the agent, although she developed breast cancer at the age of 21 months (Andervont and Dunn, 1950*b*). Thus there may be exceptions in both early and late developing tumours, as shown in the present study with dried tumour tissue and in the experiments of Andervont and Dunn with fresh tumour tissues. Biological tests of some tumours appearing at a late age, as shown in the present experiments, revealed the agent, although they failed in other tumours of similar late age. It is not known how far the recent observation of Andervont (1950*b*) that small quantities of the agent may only be ascertained by observing at least one or even two generations of the descendants of the inoculated test mice would be helpful, but it is certainly worth trying in any future tests of mammary tumours appearing at a late age. In some mammary cancers which developed up to the age of 12 months, the agent could not be detected on several occasions in the present study. Mammary tumour of No. 67 $F_1$ female at the age of 10 months, two tumours of No. 9 and 10 $F_2$ females at the age of 9·5 months and one tumour of No. 20 $F_3$ hybrid at the age of 8 months all failed to show the presence of the agent. The last three females were descendants of No. 69 $F_1$ hybrid, whose tumour appeared at 17 months and failed to reveal the agent. All tumours which developed in later generations of No. 69 $F_1$ hybrid were found to possess the agent. Andervont (1945*b*, 1950*a*) also failed to detect the agent in mice with tumours at 8, 12, 14, 15 and 16 months. On the basis of the study of parent-offspring correlation shown in pedigree charts, it appears probable that the negative tumours also harboured the agent possibly in quantities too small to be detected by the tests applied.

In conclusion it may be stated that the results of the biological tests for the presence of the agent in desiccated mammary tumours which developed in the hybrid mice indicate a gradual accumulation of factors responsible for the appearance of mammary cancer. It appears that there may be a certain threshold below which it is difficult or not possible to show the agent in some mammary tumours, at least by the methods so far employed, and only after combined influence of inbreeding and intensive hormonal stimulation the presence of the agent may become detectable. There is no reason to consider the biological tests as inadequate, in view of the previously reported constant positive results with dried agent-containing tissue, and also in view of the negative results occasionally recorded by other workers with fresh material even from tumours which developed early in life. Further, negative tests for the agent in dried tissue of an 86th transplant of a mammary tumour which originally harboured the agent (Dmochowski, 1952) have been confirmed with fresh tissue of a 101st serial passage of the same tumour. The negative findings may have been due either to small quantities of the agent present in some tumours or to the kind of relationship of the agent to other cell constituents in these tumours, such as close integration with these constituents, different from that in other tumours which were found positive in the biological tests.

### *Microscopical Appearance of Mammary Tumours.*

In view of the availability of a large number of tumours of a comparatively uniform origin a microscopical study of these tumours was made, and also an attempt to correlate the morphological appearance of these tumours with the presence or absence of the agent. Dunn (1945) and Andervont and Dunn (1950*a*) pointed out the difficulties encountered in any classification of mammary tumours, but having a large number of mammary tumours available they made an attempt to classify these tumours into four main types : Type A—with acinar structure predominating ; Type B—showing a variable pattern, composed either of solid groups of epithelial cells, or single layers of cuboidal cells having cystic spaces or growing as capillary projections into cysts or cords of cells separated by an abundant connective tissue ; Type C—with many epithelial-lined cysts, their epithelial lining invested by a layer of spindle cells ; Type D—adeno-acanthoma, with large areas composed of squamous elements (Andervont and Dunn, 1950*b*). Types A and B corresponded with Type I and Types C and D corresponded with Type II of a previous classification (Andervont and Dunn, 1948*a*). The latest classification was adopted in the present study, as it served as a useful basis for a comparison with the findings of the extensive studies of Andervont and Dunn (1950*b*) on mice maintained in the United States.

For comparison with the mammary tumours of C57 × RIII hybrids of the present experiments, a group of 141 mammary tumours which developed in 128 RIII high-cancer-strain mice was available. Of these mice, seven had two tumours and three had three tumours. Seventy-one tumours or 50·4 per cent were of Type A and arose at an average age of 232 days (126 to 430 days); sixty-eight or 48·2 per cent belonged to Type B and developed at an average age of 269 days (145 to 471 days); one tumour (0·7 per cent) was of Type D and was observed at the age of 409 days; one tumour was a carcino-sarcoma and arose at the age of 407 days. As can be seen the majority of tumours in this agent-harbouring strain could be divided into roughly equal numbers of Type A and Type B tumours.

The results of the study of 377 mammary tumours in 327 C57 × RIII hybrid mice are shown in Table XIV. Among the 327 tumorous mice, 32 had two tumours and 9 had three tumours. As shown in Table XIV, the majority of tumours were of Type B. Most of the tumours (70·6 per cent) developed during the first 12 months, and among them 53 were of Type A (19·7 per cent) and 215 (79·9 per cent) were Type B tumours; one unclassified mammary carcinoma also belonged to this group of early tumours. After the first 12 months, 21 tumours were of Type A (19·4 per cent), 77 tumours belonged to Type B (71·4 per cent), 9 tumours were of Type D (8·3 per cent), and 1 tumour was unclassified. An analysis of the distribution of the different types of tumours in the progeny of tumorous and non-tumorous (C57 × RIII)$F_1$ hybrids revealed that the progeny of both types of females had a number of each type of tumour similar to that found in the total number of all tumours. Among the 120 mammary tumours of the descendants of tumorous hybrid females, there were 19 (15·8 per cent) of Type A, 99 (82·5 per cent) of Type B, and 2 (1·7 per cent) of Type D. Among the 225 tumours in the forcibly-bred progeny of non-tumorous hybrid females, 50 (22·2 per cent) belonged to Type A, 166 (73·8 per cent) to Type B, 7 (3·1 per cent) to Type D, and 2 (0·9 per cent) were unclassified. There were 32 mammary tumours examined in the progeny of a non-tumorous hybrid female which had been bred in a normal way; five (15·6 per cent) were of Type A and 27 (84·4 per cent) belonged to Type B. Among the multiple tumours, two or three types of tumours were frequently encountered in the same female. There was no difference in the distribution of types of tumours in the progeny of hybrids which had been subjected to hormonal stimulation of varying intensity.

No correlation was observed between the type of tumour and its location or size or rate of growth or litter sequence of the tumour-bearing mice or age of the animal at which the tumour developed, except in the case of Type D tumours, all of which developed in mice older than 12 months. Further, there was no correlation between the types of tumours in litter mates or between tumours of mothers and offspring.

Similar observations were reported by Andervont and Dunn (1950*b*) on (C × C3H) susceptible hybrids, in which they found 38 per cent of Type A tumours, 44 per cent of Type B, 9 per cent of Type C, and 8 per cent of Type D. They observed a correlation between the age at which mammary tumours appeared and the type encountered, as in mice of up to 19 months of age 63 per cent were Type A tumours and 33 per cent Type B tumours, and in older mice 31 per cent belonged to Type A and 48 per cent to Type B. In another study (Andervont, 1950*a*) 38 per cent of tumours were of Type A and 46 per cent Type B, and in

TABLE XIV.—*Incidence of Mammary Tumours in C57 × RIII Hybrids According to Histological Appearance.*

| Type of tumour. | Age in months. | | | | | | | | | | | | | | | | | | | Total. | Percentage. |
|---|---|---|---|---|---|---|---|---|---|---|---|---|---|---|---|---|---|---|---|---|---|
| | 4. | 5. | 6. | 7. | 8. | 9. | 10. | 11. | 12. | 13. | 14. | 15. | 16. | 17. | 18. | 19. | 20. | 21. | 23. | | |
| A | 1 | – | 1 | 1 | 5 | 14 | 12 | 11 | 8 | 8 | 4 | 3 | 2 | 1 | 2 | 1 | – | – | – | 74 | 19·6 |
| B | 1 | 1 | 12 | 13 | 26 | 51 | 38 | 39 | 34 | 20 | 16 | 14 | 8 | 8 | 4 | 4 | 1 | 1 | 1 | 292 | 77·5 |
| D | – | – | – | – | – | – | – | – | – | – | 2 | – | 1 | 5 | 1 | – | – | – | – | 9 | 2·4 |
| Unclassified | – | – | – | – | – | – | – | 1 | – | – | – | – | 1 | – | – | – | – | – | – | 2 | 0·5 |
| Total | 2 | 1 | 13 | 14 | 31 | 65 | 50 | 51 | 42 | 28 | 22 | 17 | 12 | 14 | 7 | 5 | 1 | 1 | 1 | 377 | 100 |

mice older than 19 months 12 per cent of tumours were Type A and 63 per cent Type B, thus showing a predominance of Type B at all ages. Andervont and Dunn (1950*b*) found Type A tumour more frequently in young mice with the agent and a greater variety of types in older mice apparently without the agent or with a weak agent. They did not detect the agent in a C strain female with a squamous type mammary tumour at a late age, yet in her progeny there appeared a Type A tumour with the agent and one squamous type cancer without the agent. In another two females of the same strain, two tumours of Type A and B respectively both harboured the agent; in their progeny all tumours with the agent were of Type A. The majority of tumours in old mice were similar to those of mice with the agent (Andervont, 1945*b*, Andervont and Dunn, 1949). In hybrid mice obtained from crossings of agent-free low-cancer-strains the majority of mammary tumours were of Types C and D, and in those obtained from one of the parents belonging to agent-free but high-cancer strain the majority of breast tumours were of Types A and B (Andervont and Dunn, 1948*a*). It appears that the genetic constitution of strains used for breeding hybrids influences at least the distribution of the various types of breast cancer.

There was no correlation in C57 × RIII hybrid females between the presence of the agent, as shown in biological tests, and the type of tumour encountered. Of the seventeen tumours in which the agent was found, ten were of Type B, six of Type A, and one of Type D, and among the mammary tumours in which the test failed to reveal the agent, four were Type B and two Type A, cancer (Table XIII). The finding of the agent in Type D mammary tumour which developed at 17 months is of interest, as it appears to indicate that not all squamous type tumours which appear comparatively late need be agent-free tumours, or possibly tumours in which for some as yet unknown reason it is difficult to detect the agent. Two agent-harbouring tumours of (C57 × RIII)$F_1$ hybrids were of Type A (11 months) and Type B (16 months), and the two other tumours in which the agent was not detected were also of Type A (10 months) and Type B (11 months). Among the fifteen agent-harbouring tumours of C57 × RIII hybrid progeny, five were Type A and arose at the age of 9 to 13 months, nine were Type B and developed at 7 to 17 months, and one Type D at the age of 17 months. Two of Type A, two of Type B, and one Type D tumour developed after the first 12 months. Among the tumours in the hybrid progeny in which the agent was not detected, one was of Type A (18 months) and three of Type B (8 to 10 months). In Andervont and Dunn's experience (1950*b*) the majority of tumours (84 per cent) in hybrids developed at the age of 18 to 29 months and the rest during the first 17 months. They also found no correlation between the microscopical appearance of a tumour and the presence of the agent. Some of the early appearing tumours in which the agent could not be detected were of Type A or B (Andervont, 1945*b*, 1950*a*). Foulds (1949) described the microscopical appearance of breast tumours in his C57 × RIII hybrids as "unremarkable". Mühlbock (1952) found the morphology of tumours in C57 × d hybrids of little assistance in the study of the part played by the agent in the development of these tumours. He also noted more unusual features in tumours presumably free of the agent compared with those harbouring the agent, yet the tumours accumulated in later litters of C57 mice following mating to high-cancer-strain males, in which the agent was presumed to be present, had an appearance like that of agent-harbouring breast tumours, in spite of their late average age of development (20 months). There appears to be a greater variety in appearance of mammary tumours in mice of strains in which the agent could not be detected (Andervont and Dunn, 1950*a*; 1950*b*; Heston, Deringer, Dunn and Levillain, 1950; Mühlbock, 1952). In the present study all tumours of Type D developed after 12 months of age. Similarly, Andervont and Dunn (1950*b*) found this type of tumour to be very rare in younger hybrid mice in which the agent could be detected, although, as shown in this study, this type of tumour even at a late age may harbour the agent. Therefore the greater frequency of squamous metaplasia in tumours of old hybrid mice, if the age of 17 months could be considered as a comparatively late age, need not indicate at least in every case their development without participation of the agent, contrary to the previous suggestion of Kirschbaum (1949). There is no doubt, however, about the greater frequency of squamous type tumours in agent-free high-cancer-strain or susceptible mice (Gardner, 1947; Heston, 1948; Heston, Deringer, Dunn and Levillain, 1950) than in similar mice with the agent, but they do appear occasionally in agent-carrying strains of mice as observed by Dunn (1945), Andervont and Dunn (1950*b*), and in the present study.

From these observations it may be concluded that no particular microscopical appearance of mammary cancer in hybrid mice can be correlated with the presence or absence of the agent, in spite of the difference in distribution of the particular types in various strains of mice. The distribution of the various types of mammary tumours appears to depend on their genetic constitution derived from the strains used for obtaining the hybrid progeny. This genetic background probably also influences the age at which the different types of tumours develop.

### GENERAL DISCUSSION.

Andervont (1945*a*) was first to propose several explanations of the appearance of mammary cancer in hybrid mice, presumably free of the agent. Gardner (1947) observed that mammary tumours in mice deficient of the agent develop late in life, grow slowly and are frequently of the squamous type. Since then a considerable body of data has accumulated, which will be discussed in an attempt to collate all the known observations on breast cancer in hybrid mice with the findings of the present study in order to reveal the respective parts played by the genetic, hormonal factors and the agent in the development of mammary tumours in these mice.

### *Genetic factors.*

The influence of the genetic constitution on the development of breast tumours in hybrid mice was revealed by the variations in the tumour incidence in hybrids obtained from crossings of female and male mice of various low-cancer strains differing in their susceptibility to tumour development in the presence of agent, and also in hybrids from females of these strains mated to agent-carrying or agent-free males of various high-cancer strains. Thus a variable but low tumour incidence was reported by Andervont and Dunn (1948*a*) in hybrids from reciprocal crosses of low-cancer-strain (C, I, C57) mice and a higher but still comparatively low tumour incidence in the hybrid progeny of low-cancer-strain (C) females and agent-free high-cancer-strain (dba-) males, in spite of increased hormonal stimulation. An even higher incidence of tumours developing at a late age (22 months or even later) was observed in hybrid progeny of susceptible low-cancer-strain (C) females and high-cancer-strain (C3H) males with the agent (Andervont, 1945*b* ; Andervont and Dunn, 1948*b*, 1949, 1950*b*). Hybrid progeny of another susceptible agent-free strain (Ax) females and agent-carrying high-cancer-strain (A) males developed no breast cancer, while the progeny of yet another agent-free susceptible strain (C3Hb) females and high-cancer-strain (C3H) males with the agent showed a high incidence of mammary rumours (Bittner, 1952*a*). Hybrid females from low-cancer-strain female mice of low susceptibility to the agent (C57 Black) and agent-harbouring high-cancer-strain (RIII) males developed a comparatively low (15 per cent) incidence of breast cancer, but at a comparatively young (10 and 14 nonths) age (Foulds, 1947, 1949). A similar incidence of tumours in hybrid mice of similar derivation has been observed by the writer, but while in Foulds' experience the distribution of tumours suggested a strong familial factor in their incidence, it was not discernible in the present study. The tumorous and also the majority of non-tumorous hybrids gave rise to progeny with tumours. Although the tumour incidence in the descendants of tumour-free (C57 × RIII)$F_1$ hybrids varied, it was comparatively high in the majority of these mice. It appears that the hybrids and their progeny in the present experiments showed both a strong tendency to tumour development and to the transmission of the agent, and neither of the tendencies seemed to be limited to certain C57 Black strain females, in spite of their low susceptibility to the agent (Dmochowski, 1948). Andervont and Dunn (1948*a*) noted a parallelism between the tendency to tumour development and susceptibility to the agent, as most of the tumours which developed in hybrids from various crosses appeared in the hybrid progeny of susceptible, even agent-free, male parents. While the tendency to breast tumour development in the hybrids of Andervont and Dunn (1950*b*) was not confined to some of their low-cancer-strain (C) mothers, the tendency to transmission of the agent was limited to certain of these females.

The influence of genetic factors may not always lead to an increase in the number of tumours, in spite of increased hormonal stimulation. Attempts to increase the tumour incidence in the progeny of hybrids, obtained by back-crossing to agent-carrying males or by brother to sister matings, and subjected to forced breeding, resulted only in a decrease in the tumour incidence (Andervont and Dunn, 1949). Contrary to these findings, in the present experiments, brother to sister matings of descendants of non-tumorous hybrid mice, also subjected to forced breedings, led to an increase in tumour incidence. It appears that the main cause of the difference between these two sets of observations was in the presence of the agent in the progeny of hybrids in the present experiments.

Genetic factors appear also to play a part in the development of breast cancer in low-cancer-strain females following mating to high-cancer-strain males, even in sublines of the same strain. While Andervont and Dunn (1950*b*) reported an incidence of 8 per cent of tumours at a late age in their susceptible low-cancer-strain (C) females, Bittner (1952*a*) observed a high incidence of 59 per cent at 18 months in the same strain females following mating to similar high-cancer-strain (C3H) males. Susceptible females of another strain (Ax) were observed by Bittner (1952*a*) to remain free of tumours after mating to agent-carrying males of two high-cancer-strains (C3H and A), and of another susceptible strain (C3Hb) to develop only a low incidence of breast cancer (3 per cent) when mated to high-cancer-strain (C3H) males. Further the same strain (C) females which showed a high incidence after mating to one (C3H) high-cancer-strain male developed a much lower incidence (30 per cent) when mated to other (A) high-cancer-strain males. Females of strains with low susceptibility may show similar differences. No tumours developed in C57 Black strain females after mating to males of different agent-carrying strains, A and C3H strains (Bittner, 1952*a*), RIII strain (Foulds, 1949) and in the present study, or d strain (Mühlbock, 1952). In other low-cancer-strain (020 and dz) females an incidence of 8 to 23 per cent was reported by Mühlbock (1952) as a result of mating to either high (d) or low-cancer-strain (020) males. However, at least in some of the observations, differences in the genetic constitution of the males must also be considered as well as possible differences in the agent they harboured.

Thus the genetic constitution plays an important part in the development of mammary tumours in hybrid mice derived from matings of various strains and even in hybrids obtained from different sublines of the same strains.

*Hormonal factors.*

The influence of intensive hormonal stimulation may also vary in hybrids of different derivations and in their progeny. Forcibly-bred (C57 × RIII)$F_1$ hybrids developed a 14 per cent incidence of tumours in the present study, while no tumours appeared in their litter mates bred in a normal way. A similar incidence of cancer was also noted by Foulds (1949) in forcibly-bred hybrids of similar origin. Andervont (1950*a*) also reported an increased tumour incidence in hybrid progeny of low-cancer-strain (C) females and agent-harbouring or agent-free high-cancer-strain (C3H) males. In both types of hybrids those with seven or eight litters showed a higher tumour incidence than those with three to five litters. The importance of hormonal stimulation is particularly evident in the origin of breast tumours in hybrid females from mice genetically predisposed to tumour development but lacking the agent (Andervont, 1950*a*). This influence of hormonal factors is not so evident in hybrids from strains with low susceptibility to the agent (Andervont and Dunn, 1948*a*), and also may not always be so apparent in hybrid progeny of agent-free high-cancer-strain males compared with that of progeny from the same but agent-carrying males (Andervont and Dunn, 1949). Although forcibly-bred progeny of both cancerous and non-cancerous hybrids in the present study developed a high, but variable, incidence of tumours, this incidence was not significantly different from that in the progeny of a normally-bred hybrid, except for the age of appearance of breast cancer, possibly because of the limited observation on progeny of only one female.

It is not known how far the consecutive number of litter from which the original hybrids and their progeny originated may have been responsible for the difference between the present observations on high breast-tumour incidence in the descendants maintained by brother to sister matings and the decreasing tumour incidence in similarly maintained progeny hybrids of different derivation (C × C3H) reported by Andervont and Dunn (1949). The hybrids and their descendants came from later litters than those in Andervont's and Dunn's experiments, which originated from the first three litters. Hybrids of similar origin (C × C3H) as well as of other origin (C3Hb × C3H) and their progeny developed a low incidence of breast tumours when derived from the first five litters, and a high incidence, both virgin and breeding, if secured from seventh to tenth litter (Bittner, 1952*a*). An increase of breast tumours in hybrid progeny from later litters of

C57 Black strain females mated to high-cancer-strain (d) males was also noted by Mühlbock (1952). While on one hand these observations may be interpreted as a result of the influence of hormonal factors, on the other hand they have been interpreted (Bittner, 1952*a* ; Mühlbock, 1952) as the result of a gradual increase of the agent during its long latent period, or as an outcome of repeated matings to agent-carrying males leading to the transmission of the agent to females.

Thus increased hormonal stimulation may be responsible for an increase in tumour incidence in hybrids, especially those from agent-free male parents, but is not always separable from that of the tumour agent or of genetic constitution.

*The mammary tumour agent.*

A consistently lower tumour incidence at higher tumour age was noted in hybrids and their progeny born to males of the same but agent-free strain (Andervont and Dunn, 1948*a*, 1949 ; Foulds, 1949 ; Andervont, 1950*a* ; Bittner, 1952*a*). The incidence varied from 25 to 100 per cent in hybrids from agent-carrying males and between 9 and 50 per cent in those from agent-free males according to the number of litters born to these hybrids (Andervont, 1950*a*), about 4 per cent of the former hybrids developing tumours at an early age, and all of the latter showing only tumours at a late age. It is not known, however, whether the genetic constitution of the agent-free males was not sufficiently different from that of agent-carrying males of the same strain to influence the observed difference in the mammary tumour incidence. Hybrid progeny from females of some (Ax) strains mated to agent-harbouring high-cancer-strain (A) males remained free of breast tumours (Bittner, 1952*a*), as well as that of females of other strains (C57) mated to high-cancer-strain (A) males with the agent (Dmochowski, 1944*b*, 1945*a*). Further, a high incidence of tumours was reported in the progeny of some hybrids derived from matings of susceptible females (C3Hb × Ax) to agent-free high-cancer-strain (C3Hb) males. The breast tumours of these hybrids may at least in part have been produced by genetic and hormonal factors, in spite of the apparent absence of the agent in the male parent. However, the presence of the agent was assumed in these tumours, because of the high incidence of breast cancer in the progeny of the tumorous hybrids (Bittner, 1952*a*). Foulds (1949), Andervont (1950*a*) and Bittner (1950) interpreted the difference in tumour incidence between hybrid progeny of agent-harbouring and agent-free high-cancer-strain males as a result of the transfer of the agent by the male, especially in view of the presence of the agent in various organs of the males (Andervont and Dunn, 1948*a* ; Dmochowski, 1949*c*, Mühlbock, 1950*b*). This does not explain, however, the appearance of tumours in the hybrid progeny of agent-free males and the assumed appearance of the agent in these animals. Further, the difference in the tumour incidence may not always be apparent. Mühlbock, (1952) noted in hybrids from some low-cancer-strain (020, dz) females mated to high-cancer-strain (d) males a similar tumour incidence to that in the hybrids from their litter-mates mated to the same but agent-free (dz) males. These two types of hybrids also induced a similar tumour incidence in susceptible test mice when used as foster mothers. Thus the transfer of the agent by the male could not always be demonstrated. Even after the 10th pregnancy of low-cancer-strain females mated to males with and without the agent, these two types of females when used as foster mothers induced a similar tumour incidence in the test mice. The late tumour age (19 to 25 months) in both types of hybrids is characteristic and hormonal stimulation was assumed to be the cause of these tumours (Mühlbock, 1952). Thus the influence of the agent present in the male parent may not always be evident, and probably the genetic constitution of the different strains may have been responsible for the different results. Hormonal stimulation may have been another factor responsible for the development of breast tumours in the hybrid progeny of agent-free males. These explanations cannot be put forward for the appearance of breast tumours in hybrids of some low-cancer-strain females mated to high-cancer-strain males without the agent. In these tumours the agent was assumed to be present because of the high incidence of tumours in several successive generations of descendants of these hybrids. The presence of a latent agent or small amounts of it in the " agent-free " males could be taken as another possibility, and an increase of the agent or its activation by crossing of two different genetic constitutions and by increased hormonal stimulation.

The development of mammary tumours in some low-cancer-strain females (Andervont, 1945*b* ; Andervont and Dunn, 1950*b*) and in many females of other low-cancer-strains (Bittner, 1952*a*), the presence of the agent in tumours of these females, in spite of their late (18 months) age of appearance (Bittner, 1952*a*), further the appearance of breast cancer, or increased incidence of tumours in the hybrid progeny of these females from late litters compared with none or low incidence in the progeny of early litters (Bittner, 1952*a*) indicate the transmission of the agent to low-cancer-strain females following repeated matings to high-cancer-strain males harbouring the agent. This conclusion is further strengthened by the failure to find a weak or attenuated agent in females of one susceptible low-cancer-strain, in spite of repeated attempts. Foster nursing of susceptible mice by these females (Andervont, 1945*b*) ; observation of several generations of progeny of mice fostered by these females (Andervont and Dunn, 1948*b*) ; exposure of young females to X radiation (Andervont and Dunn, 1950*b*) oestrogenic stimulation of the females (Andervont, 1950*a*), all failed to reveal the agent. Finally the possibility of an increase of the agent in older females of this strain was not substantiated by the observation of similar tumour incidence in hybrid progeny of both old and young females (Andervont and Dunn, 1949). The development of mammary tumours in the descendants of both tumorous and the majority of non-tumorous hybrid progeny of C57 females which remained tumour-free after mating to agent-carrying males in the present experiments as well as the detection of the agent in the majority of the tumours tested indicate the transfer of the agent by RIII high-cancer-strain males. Although not all (C57 $\times$ RIII)$F_1$ hybrids developed breast cancer, the transfer of the agent must have taken place in the majority of the females. Similarly, Bittner (1952*a*) noted a high incidence of tumours and the presence of the agent in the descendants of cancerous and cancer-free hybrid progeny of certain strain females mated to agent-carrying males. The presence of the agent was assumed in mammary tumours which appeared in hybrid progeny of later litters of some C57 females mated to high-cancer-strain (d) males (Mühlbock, 1952) because of the high tumour incidence, in spite of the late age at which the tumours appeared. The occasional transfer of the agent to the hybrid progeny in mother's milk was therefore accepted by Mühlbock (1952). In some cases the agent was detected in the tumours of hybrid progeny and their low-cancer-strain mothers, in other cases all attempts failed to reveal it (Andervont and Dunn, 1948*b*, 1950*b*).

Thus in low-cancer-strain females mated to agent-carrying males as well as in their hybrid progeny a variable incidence of cancer was reported, with some tumours revealing the agent in biological tests and some not; in other tumours the presence of the agent was assumed because of high incidence of breast cancer in the descendants of tumorous and/or non-tumorous hybrid females.

The transfer of the agent by high-cancer-strain males accepted as the most likely source of the origin of the agent present in mammary tumours in hybrid females, there remains the problem of the way in which the agent is transferred to low-cancer-strain females. In view of the failure of Andervont and Dunn (1949) to detect the agent in some low-cancer-strain females after mating to agent-possessing males, they suggested that the females either are not affected by the agent or acquire it in insufficient amounts because of the transfer of the agent in an attenuated form *in utero*. As a result of this transfer, only few tumours in the hybrid progeny develop early and harbour the agent, while the majority of tumours develop late without participation of the agent. Transfer of the agent to embryos, however, appears to be doubtful, because of the absence of the agent in high-cancer-strain embryos (Dmochowski, 1949*c*; Hummel and Little, 1949). The passage of the agent from embryos, should they become infected *in utero*, is also questionable because of the reported neutralisation of the agent by placenta (Hummel, Little and Eddy, 1949). Transmission of the agent *in utero* would also contradict the basis of the discovery of the agent itself. The increase in tumour incidence in hybrid progeny of later litters (Andervont and Dunn, 1949; Bittner, 1952*a*; Mühlbock, 1952) and the high incidence of tumours observed in the descendants of later litters from hybrid females observed in the present experiments may indicate either a transfer of the agent to females repeatedly mated to agent-carrying males and/or gradual increase in the agent, transferred by mating, during its long latent period under the influence of hormonal stimulation (repeated pregnancies). The low incidence of tumours observed in the hybrid progeny of some derivations need not necessarily be interpreted in the same way as the small number of tumours induced in mature mice which had been given the agent, as suggested by Mühlbock (1952). A high incidence of tumours obtained in mature mice of some strains after repeated injections of material containing the agent (Dmochowski, 1945*a*; Mühlbock, 1952), and a small incidence in mature mice of other strains (Bittner, 1952*b*), indicate that the genetic constitution is the more likely explanation for the different tumour incidences in hybrids of various derivations. Transmission of the agent by the sperm to low-cancer-strain females, followed by its transmission to their hybrid progeny in the milk of these females (Bittner, 1952*a*), appears to be the most likely way in which the agent gains access to hybrid females, although in females of some strains it may only occasionally take place (Mühlbock, 1952). The observation of several generations of descendants of hybrid females can give a clear picture whether the transfer of the agent has taken place or not, as shown in the present study. Under the same experimental conditions some hybrid females may develop mammary tumours while their litter-mates may fail to show tumours; their descendants may show a similar variation in their behaviour. Thus, the study of the behaviour of the hybrid progeny of low-cancer-strain females, especially of the descendants of later litters of the hybrid progeny, can only decide whether or not the agent has been transferred by the male parent.

The observations on tumour development and the presence of the agent in the descendants of the hybrid progeny obtained by mating C57 Black strain females to agent-carrying RIII strain males, made in the present experiments, appear to indicate an interesting possibility in the agent-host relationship which may be the outcome of gradual increase in the amount of the agent or its activation in some hybrid females, while in other hybrid females it appears in quantities large enough to be detected immediately with the development of breast cancer in the first generation of hybrid progeny. This may be on one hand the result of individual variations in the genetic constitution of hybrid females, and on the other hand the outcome of varying quantities of the agent which the hybrid females obtain from their low-cancer-strain mothers mated to high-cancer-strain males. Within the framework of individual differences in the genetic make-up and/or quantities of the agent obtained, inbreeding combined with hormonal stimulation also exerts its influence. Thus we have the situation, encountered in the present experiments, that under the same experimental conditions some hybrid females develop mammary cancer and others (even their litter mates) do not show breast cancer; some of the tumorous females do and others do not reveal the agent; some of the progeny of the cancerous hybrid females may develop mammary tumours which again may or may not reveal the agent, and other progeny of the same cancerous females do not develop tumours; the progeny of non-cancerous hybrid females may develop tumours which contain the agent. The development of breast cancer in some of the (C57 $\times$ RIII)$F_1$ hybrids only after forced breeding may be explained by the transmission of small quantities of the agent by at least some of the C57 Black strain mothers after mating to RIII agent-carrying males. The high, although varied, tumour incidence in the progeny of these hybrids may be explained by the origin of these females as well as of their progeny from late litters and therefore an increase or activation of the agent in these litters, enhanced by inbreeding and increased hormonal stimulation. Andervont and Dunn (1950*b*) also observed that under the same conditions some low-cancer-strain females developed and others failed to develop breast cancer, and some females with tumours possessed and others lacked the agent. There was also no correlation between the presence of the agent in these females and its presence in their hybrid progeny, cancerous females with or without the agent giving rise to hybrid progeny with only late tumours apparently without the agent, or to progeny with both early tumours with the agent and late tumours without the agent, although they were litter mates. Therefore, again, the presence or absence of the tumour and/or the agent in the mother did not necessarily involve the presence or absence of the agent in her progeny. Andervont and Dunn (1950*b*) noted on several occasions among the hybrid progeny with late tumours that their litter mates had early tumours with the agent, and on one occasion a hybrid female with an early tumour harbouring the agent had progeny with either no tumours or only late tumours without the agent, which suggested the disappearance of the agent (Andervont and Dunn, 1949). Bittner (1952*a*) also reported on the hybrid progeny of some derivations with low mammary tumour incidence that some of them gave rise to descendants with a high incidence of tumours harbouring the agent. Thus the variable results appear to be due to variations in the genetic make-up of both the hybrid females and their progeny, which in turn leads to variations in the amount of the agent transmitted. A more favourable genetic make-up may account for an increased amount of the agent and its detection. or the agent may be present in a constant amount and its detection is entirely dependent on the genetic constitution and hormonal factors encountered in various individual hybrids.

Transmission of the agent by agent-carrying high-cancer-strain males is further strengthened by the observation of Andervont, Shimkin, and Bryan (1942), which disposes of the possibilty of a contagion of the hybrids or their low cancer-strain mothers and also by the absence of the agent in excreta of mice (Dmochowski and Passey, 1950*a*, 1950*b* ; Mühlbock, 1950*a*). This, of course, does not imply the transmission of the agent to all females, and even those which had obtained the agent and developed tumours need not necessarily transmit it to their progeny. It appears also that the paternal contribution, resulting in different tumour incidences in hybrid progeny of the same low-cancer-strain females and males of different high-cancer-strains, besides being genetic may also be of non-genetic origin, because of the observed differences in the agent present in these various high-cancer-strains (Dmochowski, 1945*b* ; Bittner and Huseby, 1946). Thus, while (C57 × RIII)$F_1$ hybrids develop breast cancer under certain conditions, no tumours appear in (C57 × A)$F_1$ hybrid females treated in a similar manner (Dmochowski, 1944*b* ; 1945*a*). Similar observations were made by Bittner (1952*a*) on hybrids of various other derivations. The appearance of mammary tumours in some low-cancer-strain females (C3Hb × Ax) mated to agent-free (C3Hb) males and the high incidence of tumours in their descendants, on the basis of which the presence of the agent was assumed although the tumours were not tested biologically (Bittner, 1952*a*), is one at the moment rather perplexing observation. Sudden " *de novo* " appearance of the agent was therefore also considered as a possible explanation of some of the findings (Andervont and Dunn, 1949 ; Bittner, 1952*a*). This consideration was based on previous observations of Bittner (1941) on the sudden appearance of the agent in some susceptible (Ax) mice, deprived of the agent by foster nursing by low-cancer-strain mothers, which remained free of the agent for seven generations, and on similar observations made recently by Bittner (1952*a*) on susceptible mice of another strain (C3Hb) which remained free of the agent for sixteen generations following foster nursing before the appearance of breast cancer in one of the females, which then gave rise to progeny with a high incidence of mammary tumours. According to the writer's opinion, these observations of a sudden appearance of the agent may only show the length of time during which the agent may lie dormant or the time required before the agent, originally present in small amounts, increases in quantity sufficient to induce breast cancer in combination with other known and unknown factors. Of the known factors, either a change in the genetic constitution of the animal concerned alone and/or increased hormonal stimulus may, at least in part, be responsible for the activation of the agent.

Bittner (1939*a*) reported the appearance of some mammary tumours in mice without the agent, and Strong (1943) stressed the influence of hormonal factors in such tumours. The difficulties encountered in the detection of the agent in breast tumours appearing late in the life of mice and the comparative ease with which it could be shown in tumours developing early (Andervont, 1950*a*), further the isolated tumours in hybrid progeny of early litters and the accumulation of tumours in hybrid mice of late litters (Mühlbock, 1952), led to the conclusion that there may be two types of mammary tumours in mice. One type of tumour would be the result of all three factors taking part in its development, the other type would be the result of hormonal and genetic factors without participation of the agent (Andervont, 1950*a*; Mühlbock, 1952). However, the possibility of the agent being involved in both types of tumours was not discounted by Andervont (1950*a*). The genetic constitution and intensive hormonal stimulation were considered adequate by Heston, Deringer, Dunn and Levillain (1950) to give rise to mammary tumours in susceptible (C3Hb) mice, originally derived from mice deprived of the agent by foster nursing, but they also stressed that absolute proof of the absence of the agent from such tumours was lacking. The appearance of only a few tumours in the hybrid progeny from reciprocal matings of these (C3Hb) mice with low-cancer-strain (C57) mice with no evidence of an increase in tumour incidence in the hybrid progeny of later litters, as originally reported by Bittner (1944) in mice of agent-carrying strains or in hybrid progeny from low-cancer-strain females and agent-carrying males, again led Heston and Deringer (1952) to suggest that some mammary tumours develop in the absence of the agent. Yet, in hybrid progeny of similar mice of other strains, Bittner (1952*a*) observed the appearance of tumours harbouring the agent. Further, the agent has been detected in some mammary tumours appearing in comparatively old hybrids, as shown by Bittner (1952*a*) and in the present study. In other late tumours as well as occasionally also in early-developing breast cancers the agent could not be detected by any, so far, employed testing procedures as noted by Andervont (1950*a*), Andervont and Dunn (1950*b*), and by the writer.

Thus, intensive hormonal stimulation combined with a suitable genetic background influence the origin of mammary tumours in hybrid mice, increase their incidence, and accelerate their appearance. There is no doubt that the mammary tumour agent takes a part in the development of breast tumours appearing up to a certain age, although not all tumours of early appearance in hybrid mice reveal its presence. The conclusion that tumours in old hybrids do not harbour the agent and are the result of a combined action of hormonal and genetic factors only should at least for the time being be suspended, in view of the discovery of the agent in some mammary tumours appearing at an older age than 15 months and the lack of correlation between any microscopical appearance of breast cancer and the presence or absence of the agent.

### SUMMARY.

1. (C57 × RIII)$F_1$ hybrid females, obtained by mating agent-free C57 Black strain females to agent-harbouring RIII high-cancer-strain males, developed a 14 per cent incidence of mammary tumours at an average age of 13 months after bearing in quick succession an average number of 6·4 litters. Their litter-mates, bred in a normal way, died free of tumours after rearing an average number of 3 litters. None of the C57 strain mothers developed breast cancer, although they lived to an average age of 19 months.

2. The descendants of tumour-free hybrid females which had been subjected to forced breeding, maintained by brother to sister matings for several generations and also forcibly bred, developed a lower incidence of tumours at a considerably higher tumour age than similarly maintained progeny of hybrid females with tumours. The difference between the progeny of these two types of hybrid females gradually became smaller in successive generations of inbreeding. The average tumour age in the normally bred progeny of one tumour-free hybrid which had been bred in a normal way was considerably higher than that in the forcibly-bred progeny of both tumorous and tumour-free hybrids, and the tumour incidence was lower than that in the descendants of cancerous hybrids but approximated that in the progeny of non-cancerous hybrids.

3. A connection between inbreeding and hormonal stimulation and the development of breast cancer in the descendants of the hybrids was revealed by an increasing number of females which developed tumours after bearing five litters in each successive generation of the progeny of both cancerous and non-cancerous hybrid females. This and the increasing number of mice with tumours born in the first litter may have also been the result of an increase in the amount or activation of the mammary tumour agent.

4. Analysis of parent-offspring correlation in the distribution of tumours in the descendants of the hybrids revealed that the progeny of tumours and of the majority of non-tumorous hybrids had a greater chance to develop cancer if the parent developed cancer than if the parent died without a tumour. Thus there was a strong indication of the presence of the agent in these hybrids and of its transmission to their progeny, which showed an increasing number of tumours following the combined influence of inbreeding and intensive hormonal stimulation. The tendency to tumour development and transmission of the agent shown by the hybrids was not limited to certain C57 strain female parents.

5. The number of tumours which arose in litters and their progeny obtained before their hybrid mothers developed cancer and in those secured after tumour appearance indicated that some hybrids appeared to harbour the agent before and after tumour development, some only after, and others only before the development of breast cancer. This may have been the result of variations in the genetic make-up of individual hybrids and their influence on the presence or transmission of the agent.

6. Biological tests for the presence of the agent in twenty-three mammary tumours were positive in seventeen and negative in the remainder of tumours. The age of tumours in which the agent was detected varied from 7 to 17 months, and that of tumours in which the agent could not be detected from 8 to 18 months. These tests, combined with the observed distribution of tumours in the descendants of hybrid mice, showed that under the same experimental conditions some hybrids developed tumours which either harboured the agent or failed to reveal it, while other hybrids, even their litter mates, died without tumours. Some of the progeny of tumorous hybrids failed to develop tumours and others of the same females developed cancer in which again the agent was either demonstrated or could not be shown. Some of the progeny of tumour-free hybrids developed tumours which harboured the agent. As there was no reason to doubt the adequacy of the tests, it is possible that the results were due to small quantities of the agent or an attenuated agent present in some tumours or to its close integration with tumour cells, or to the agent present in a constant amount in animals with a variable and low but gradually increasing susceptibility. There may be a certain threshold below which it is difficult to detect the agent in some tumours, and it becomes demonstrable in other tumours after inbreeding combined with hormonal stimulation.

7. The study of microscopical appearance of 377 mammary tumours in C57 × RIII hybrid females and of 141 tumours in RIII strain mice showed no correlation between the appearance of a tumour and its location, size, rate of growth, litter sequence of tumour-bearing mice, or their age, except in squamous type tumours.

which as a rule developed in older mice above the age of 12 months. There was also no correlation between the appearance of mammary tumours in parents and their offspring. No connection was found between the appearance of tumours in hybrid mice and the presence or absence of the agent in these tumours. The agent was also demonstrated in a tumour of the adeno-acanthoma type which developed at an age of well over 12 months. The frequency of occurrence of various types of mammary cancer and the age at which they develop appear to be influenced by the genetic constitution of hybrid mice.

8. The implications of these observations in correlation with those recorded on hybrids of other derivations are discussed.

Acknowledgment is due to Mrs. A. Flaks for valuable technical assistance, and to Miss Heather M. T. Hebson for the care of animals during the course of this study.

### REFERENCES.

ANDERVONT, H. B.—(1940) *J. nat. Cancer Inst.*, **1**, 135.—(1945*a*) ' A Symposium on Mammary Tumours in Mice,' publ. Amer. Ass. Adv. Sci., by Members of the Staff of the Nat. Cancer Inst., Washington 25, D.C., p. 140.—(1945*b*) *J. nat. Cancer Inst.*, **5**, 391.—(1949*a*) *Ibid.*, **10**, 193.—(1949*b*) *Ibid.*, **10**, 201.—(1950*a*) *Ibid.*, **11**, 73.—(1950*b*) *Ibid.*, **11**, 545.

*Idem* AND DUNN, TH. B.—(1948*a*) *J. nat. Cancer Inst.*, **8**, 227.—(1948*b*) *Ibid.*, **8**, 235.—(1949) *Ibid.*, **9**, 89.—(1950*a*) *Ibid.*, **10**, 895.—(1950*b*) *Ibid.*, **10**, 1157.

*Idem*, SHIMKIN, M. B., AND BRYAN, W. R.—(1942) *Ibid.*, **3**, 309.

BAGG, H. J., AND JACKSEN, J.—(1937) *Amer. J. Cancer*, **30**, 539.

BITTNER, J. J.—(1939*a*) *Publ. Hlth. Rep., Wash.*, **53**, 257.—(1939*b*) *Ibid.*, **54**, 1113.—(1941) *Cancer Res.*, **1**, 113.—(1942) *Ibid.*, **2**, 710.—(1943) *Ibid.*, **3**, 441.—(1944) *Ibid.*, **4**, 159.—(1950) *Ibid.*, **10**, 204.—(1952*a*) *Ibid.*, **12**, 387.—(1952*b*) *Ibid.*, **12**, 510.

*Idem* AND HUSEBY, R. A.—(1946) *Ibid.*, **6**, 235.

DMOCHOWSKI, L.—(1944*a*) *Brit. J. exp. Path.*, **25**, 119.—(1944*b*) *Ibid.*, **25**, 138.—(1945*a*) *Ibid.*, **26**, 192.—(1945*b*) *Ibid.*, **26**, 267.—(1946) *Ibid.*, **27**, 391.—(1948) *Brit. J. Cancer*, **2**, 94.—(1949*a*) " Report of the Dept. of Exp. Path. and Cancer Res., University of Leeds," in 24th *Annual Report of the Yorkshire Council, Brit. Emp. Cancer Campgn.*, p. 8.—(1949*b*) *Rep. Brit. Emp. Cancer Campgn.*, **27**, 162.—(1949*c*) *Brit. J. Cancer*, **3**, 525.—(1950*a*) " Report of the Dept. of Exp. Path. and Cancer Res., University of Leeds ", in 25th *Annual Report of the Yorkshire Council, Brit. Emp. Cancer Campgn.*, p. 7.—(1950*b*) *Rep. Brit. Emp. Cancer Campgn.*, **28**, 169.—(1951*a*) " Report of the Dept. of Exp. Path. and Cancer Res., University of Leeds ", in 26th *Annual Report of the Yorkshire Council, Brit. Emp. Cancer Campgn.*, p. 9.—(1951*b*) *Rep. Brit. Emp. Cancer Campgn.*, **29**, 150.—(1952) *Brit. J. Cancer*, **6**, 249.

*Idem* AND GYE, W. E.—(1943) *Brit. J. exp. Path.*, **24**, 223.

*Idem* AND ORR, J. W.—(1949) *Ibid.*, **3**, 520.

*Idem* AND PASSEY, R. D.—(1950*a*) " Report of the Dept. of Exp. Path. and Cancer Res., University of Leeds ", in 25th *Annual Report of the Yorkshire Council, Brit. Emp. Cancer Campgn.*, p. 6.—(1950*b*) *Rep. Brit. Emp. Cancer Campgn.*, **28**, 167.

DUNN, TH. B.—(1945) ' A Symposium on Mammary Tumours in Mice,' publ. Amer. Ass. Adv. Sci., by Members of the Staff of the Nat. Cancer Inst., Washington 25, D.C., p. 13.

FOULDS, L.—(1947) *Brit. J. Cancer*, **1**, 362.—(1949) *Ibid.*, **3**, 230.

GARDNER, W. U.—(1941) *Cancer Res.*, **1**, 345.—(1947) *Ibid.*, **7**, 37.

HESTON, W. E.—(1948) *Brit. J. Cancer*, **2**, 87.
*Idem* AND DERINGER, M. K.—(1952) *J. nat. Cancer Inst.*, **13**, 167.
*Iidem*, DUNN, TH. B., AND LEVILLAIN, W. D.—(1950) *Ibid.*, **10**, 1139.
HUMMEL, K. P., AND LITTLE, C. C.—(1949) *Cancer Res.*, **9**, 129.
*Iidem*, AND EDDY, M. S.—(1949) *Ibid.*, **9**, 135.
KIRSCHBAUM, A.—(1949) *Ibid.*, **9**, 93.
MÜHLBOCK, O.—(1950*a*) *Acta physiol. pharmacol. Neerl.*, **1**, 645.—(1950*b*) *J. nat. Cancer Inst.*, **10**, 861.—(1952) *Ibid.*, **12**, 819.
MURRAY, W. S., AND LITTLE, C. C.—(1939) *Amer. J. Cancer*, **37**, 536.
STRONG, L. C.—(1943) *Proc. Soc. exp. Biol. N.Y.*, **53**, 257.